# Cylindrospermopsin- and Deoxycylindrospermopsin-Producing *Raphidiopsis raciborskii* and Microcystin-Producing *Microcystis* spp. in Meiktila Lake, Myanmar

**DOI:** 10.3390/toxins12040232

**Published:** 2020-04-07

**Authors:** Andreas Ballot, Thida Swe, Marit Mjelde, Leonardo Cerasino, Vladyslava Hostyeva, Christopher O. Miles

**Affiliations:** 1 Norwegian Institute for Water Research, Gaustadalléen 21, N-0349 Oslo, Norway; thida.swe@niva.no (T.S.); marit.mjelde@niva.no (M.M.); vladyslava.hostyeva@niva.no (V.H.); 2 Forest Research Institute, 15013 Yezin, Myanmar; 3 Department of Natural Sciences and Environmental Health, University of South- Eastern Norway, Gullbringvegen 36, N-3800 Bø, Norway; 4 Department of Sustainable Agro-ecosystem and Bioresources, Research and Innovation Centre, Fondazione Edmund Mach, Via E. Mach 1, 38010 San Michele all’Adige, Italy; leonardo.cerasino@fmach.it; 5 National Research Council, 1411 Oxford Street, Halifax, NS B3H 3Z1, Canada; christopher.miles@nrc-cnrc.gc.ca

**Keywords:** Meiktila Lake, *Raphidiopsis*, *Microcystis*, cylindrospermopsin, deoxycylindrospermopsin, microcystin

## Abstract

Meiktila Lake is a shallow reservoir located close to Meiktila city in central Myanmar. Its water is used for irrigation, domestic purposes and drinking water. No detailed study of the presence of cyanobacteria and their potential toxin production has been conducted so far. To ascertain the cyanobacterial composition and presence of cyanobacterial toxins in Meiktila Lake, water samples were collected in March and November 2017 and investigated for physico-chemical and biological parameters. Phytoplankton composition and biomass determination revealed that most of the samples were dominated by the cyanobacterium *Raphidiopsis raciborskii*. In a polyphasic approach, seven isolated cyanobacterial strains were classified morphologically and phylogenetically as *R. raciborskii*, and *Microcystis* spp. and tested for microcystins (MCs), cylindrospermopsins (CYNs), saxitoxins and anatoxins by enzyme-linked immunosorbent assay (ELISA) and liquid chromatography–mass spectrometry (LC–MS). ELISA and LC–MS analyses confirmed CYNs in three of the five *Raphidiopsis* strains between 1.8 and 9.8 μg mg^−1^ fresh weight. Both *Microcystis* strains produced MCs, one strain 52 congeners and the other strain 20 congeners, including 22 previously unreported variants. Due to the presence of CYN- and MC-producing cyanobacteria, harmful effects on humans, domestic and wild animals cannot be excluded in Meiktila Lake.

## 1. Introduction

Many lakes and reservoirs worldwide are affected by periodic cyanobacterial dominance or even cyanobacterial blooms. Such mass developments of cyanobacteria are typical for eutrophic conditions and are often induced by nutrient enrichment caused by increased agricultural, urban and industrial activities and are also expected to increase due to regional and global climate change [1]. Various cyanobacterial species forming such blooms are potential producers of hepatotoxic or neurotoxic compounds and their presence is often associated with animal poisonings and a threat to human health [2].

**Figure 1:**
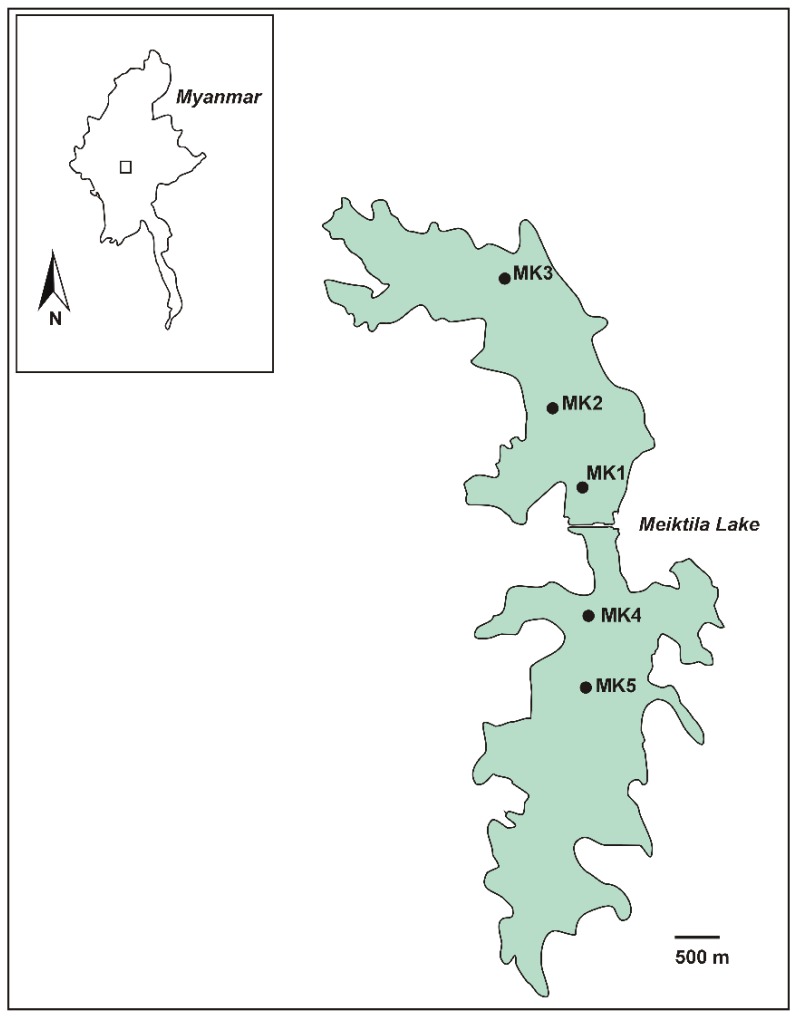
Map of Meiktila Lake. The map shows the locations of water sampling (Stations MK1-MK5). The location of Meiktila Lake in Myanmar is shown in the inset.

Myanmar is characterized by the presence of several natural lakes and numerous man-made reservoirs. Meiktila Lake is one of the numerous reservoirs in Myanmar and was built in ancient times, dating from an unknown period [3] but most likely in the reign of King Narapathisithu (1173–1210) [4]. Today the lake is divided by a dam into a northern and a southern part (Figure 1) [5]. Meiktila Lake is exposed to sedimentation due to deforestation in the catchment and especially the northern part has been partially filled with sediment over a period of more than 100 years [6]. The priority use of water from Meiktila Lake is drinking water, water for domestic purposes and for irrigation, although the lake is also polluted with domestic waste water, street runoff and solid waste [6,7].

Only limited information is available about the limnological characteristics of Meiktila Lake and other freshwater habitats in Myanmar. In 1995, a study of the algal flora of Meiktila Lake was reported [4]. A recent study described the investigation of physical parameters, macrophyte and phytoplankton composition in the period 2011–2014 in Meiktila Lake [5]. Twenty taxa of aquatic macrophytes including helophytes have been documented in Meiktila Lake [5]. Several heterocytous cyanobacterial taxa, e.g., *Anabaena*, *Anabaenopsis* and *Calothrix* and a few nonheterocytous cyanobacterial taxa e.g., *Aphanocapsa*, *Chroococcus*, *Microcystis*, *Arthrospira* and *Oscillatoria* have been reported but not further investigated [4,5]. Neither study mentioned the presence of the cyanobacterium *Raphidiopsis raciborskii* (formerly *Cylindrospermopsis raciborskii*) (Woloszynska) Aguilera, Berrendero Gómez, Kastovsky, Echenique & Salerno nor the presence of the microcystin (MC) and cylindropsermopsin (CYN) groups of cyanobacterial toxins, which are documented from many lakes in Asia [8,9,10].

We suspect that the number of cyanobacterial species documented to date in Meiktila Lake was underestimated and that various toxin-producing cyanobacteria were present in the cyanobacterial community. There is clearly a lack of information about cyanobacteria and the production of cyanobacterial toxins in Meiktila Lake and the recently described potentially toxic cyanobacterium *Microcystis* is most likely not the only potential toxin-producing cyanobacterium. The ongoing pollution of the lake suggests the potential for occurrence of more frequent severe cyanobacterial blooms, which would have a negative impact on the use of the lake for drinking water and domestic purposes by the residents. This study aimed therefore to investigate the presence of cyanobacteria and their potential toxins in Meiktila Lake, applying modern analytical methods in a polyphasic approach to elucidate in detail the cyanobacterial composition, phylogeny and toxin production and toxin profiles.

## 2. Results

### 2.1. Physico-Chemical Parameters

At both sampling dates in March and November 2017, all sampling points in Meiktila Lake were characterized by water temperatures of 26.2–28.0 °C, pH of 8.5–9.3 and conductivities of 580–729 μS cm^−1^. Secchi depth was between 0.8 m (at sampling point MK1) and 1.8 m (at MK3). Total phosphorus and total nitrogen concentrations were 12–23 and 360–570 μg L^−1^, respectively.

### 2.2. Phytoplankton Community

At all sampling stations, and at both sampling dates, cyanobacteria were the dominant group in the phytoplankton in both parts of Meiktila lake together with diatoms (Bacillariophyceae) Cryptophyceae, Chlorophyceae and Euglenophyceae (Table 1). The most dominant cyanobacterium was *R. raciborskii*, which comprised biomasses between 0.2 and 1.9 mg L^−1^ fresh weight (FW), or 27%–91% of the cyanobacterial biomass at the sampling points MK1, MK2, MK4 and MK5. Other cyanobacteria present in the samples belonged to the genera *Aphanocapsa*, *Aphanothece*, *Chroococcus*, *Merismopedia*, *Limnothrix*, *Microcystis*, *Planktolyngbya*, *Planktothrix*, *Sphaerospermopsis* and *Synechococcus*. They together comprised biomasses between 0.06 and 1.2 mg L^−1^ cyanobacterial wet weight. At MK3, the biomass of *R. raciborskii* at both sampling dates (0.02–0.08 mg L^−1^) was lower than at the other sampling points MK1, MK2, MK4 and MK5 (0.20–1.94 mg L^−1^) (data not shown). The *Microcystis* biomass was lower than the *Raphidiopsis* biomass at all sampling points and sampling dates and ranged from 0.003 to 0.16 mg L^−1^ (data not shown).

### 2.3. Morphological and Phylogenetic Characterization

**Figure 2:**
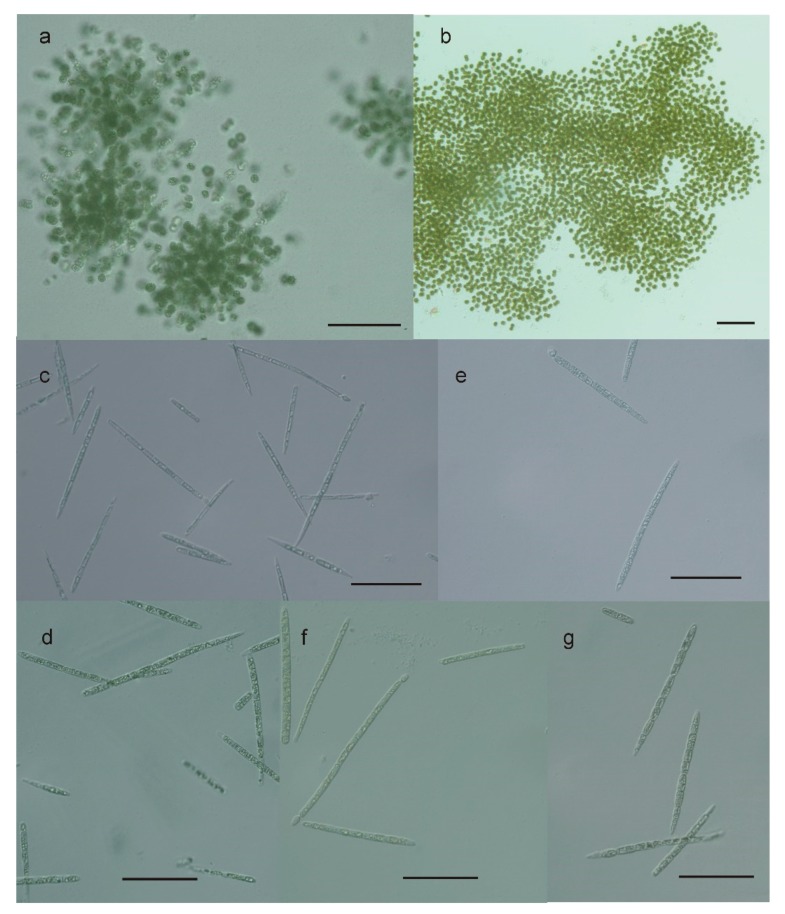
Micrographs of cyanobacteria investigated in this study. (**a**) *Microcystis novacekii* (AB2017/14); (**b**) *Microcystis aeruginosa* (AB2017/15); (**c**) *Raphidiopsis raciborskii* (AB2017/05); (**d**) *Raphidiopsis raciborskii* (AB2017/09); (**e**) *Raphidiopsis raciborskii* (AB2017/12); (**f**) *Raphidiopsis raciborskii* (AB2017/13); (**g**) *Raphidiopsis raciborskii* (AB2017/16). Scale bars indicate 50 µm.

Seven potentially toxin-producing cyanobacterial strains were isolated from Meiktila Lake (Table 2). Based on morphological features, e.g., presence and form of colonies or filaments, vegetative cells and heterocytes, five of the isolated cyanobacterial strains were identified as *R. raciborskii* and two strains as *M. aeruginosa* and *M. novacekii*, respectively (Figure 2). However, Harke et al. [11] suggested all *Microcystis* warrant placement into the same species complex. Therefore, we use “*Microcystis*” instead of species names in the following parts of the manuscript.

The *Raphidiopsis* strains were mostly characterized by straight tapered filaments. The filament length and width varied between 8.8–90 × 1.9–5.8 µm. Heterocytes were observed in some filaments of all isolated strains. Akinetes were not observed in any of the investigated strains. As in the cultured strains, only a few of the filaments possessed heterocytes in the environmental samples. The two *Microcystis* strains were characterized by cell diameters ranging from 4.2 to 6.6 µm (strain AB2017/15) and from 3.7 to 5.8 µm (strain AB2017/14) (data not shown).

**Figure 3:**
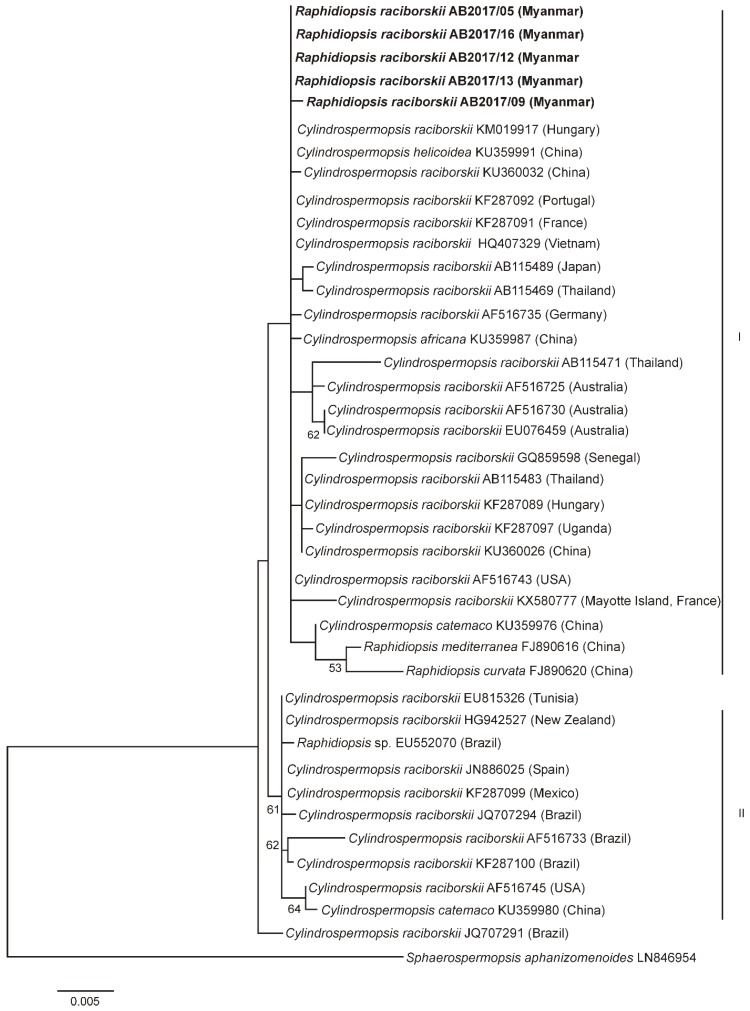
ML tree based on partial 16S rRNA gene sequences of 40 *Raphidiopsis*/*Cylindrospermospis* strains. Outgroup = *Sphaerospermopsis aphanizomenoides* (LN846954). Cluster I includes *Cylindrospermopsis* and *Raphidiopsis* strains from Asia, Europe, Africa, Australia and North America, cluster II includes strains from North and South America (USA, Mexico, Brazil), North Africa (Tunisia), Southwest Europe (Spain) and New Zealand. Strains from this study are marked in bold. Bootstrap values above 50 are included. The scale bar indicates 0.5% sequence divergence.

**Figure 4:**
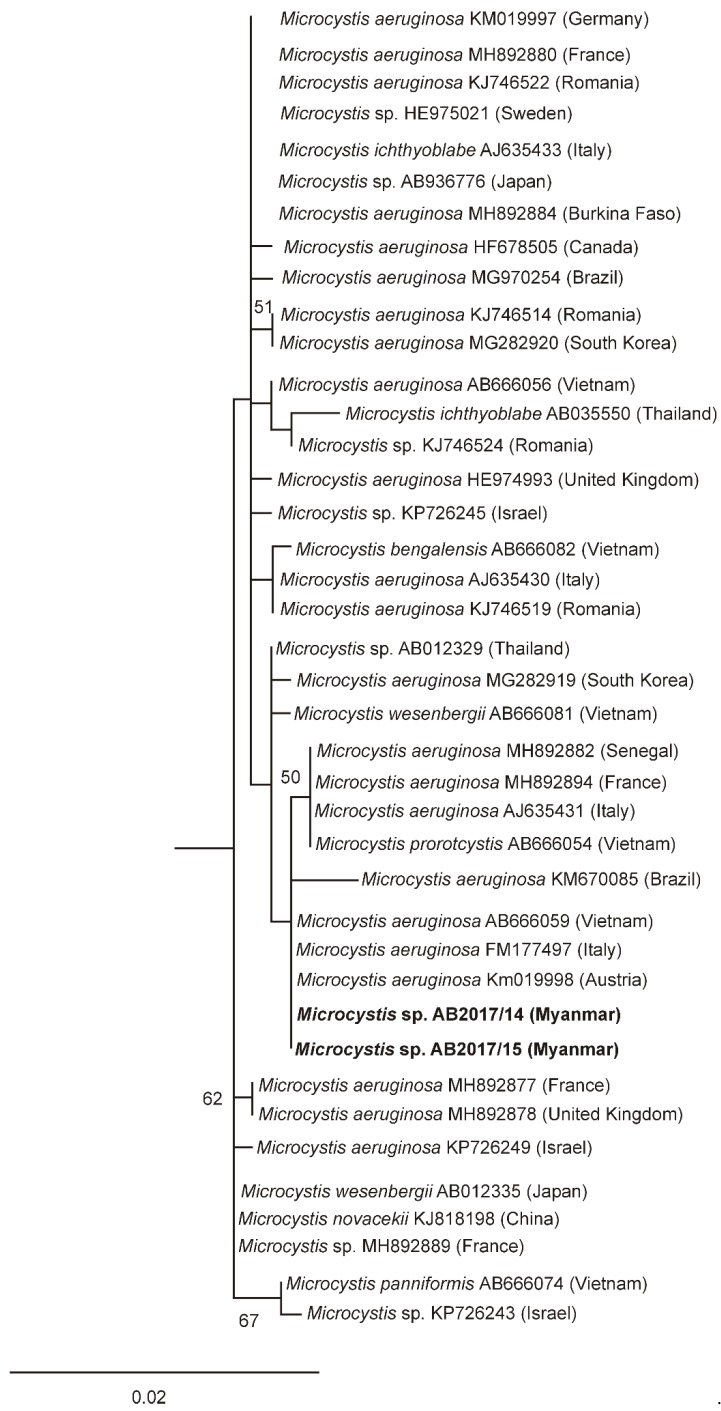
ML tree based on partial 16S rRNA gene sequences of 40 *Microcystis* strains. Outgroup = *Chroococcus subviolaceus* (MF072353). Strains from this study are marked in bold. Bootstrap values above 50 are included. The scale bar indicates 2% sequence divergence.

The morphological determination of the isolated strains was supported by phylogenetic analyses (Figure 3; Figure 4). Phylogenetic relationships of the investigated strains are presented in the maximum-likelihood (ML) tree of the 16S rRNA gene of *Cylindrospermopsis*/*Raphidiopsis* (Figure 3) and a separate ML tree of the *Microcystis* 16S rRNA gene (Figure 4). In the ML tree in Figure 3, the *Raphidiopsis* strains from Meiktila Lake grouped together with 16S rRNA gene sequences derived from *Cylindrospermopsis* and *Raphidiopsis* strains from Asia, Europe, Africa, Australia and North America (cluster I). The CYN-producing and nonCYN-producing *Raphidiopsis* strains from Meiktila Lake could not be distinguished phylogenetically using 16S rRNA gene and had similar 16 rRNA gene sequences (Figure 3). In cluster II, strains from North and South America, (USA, Mexico, Brazil), North Africa (Tunisia), Southwest Europe (Spain) and New Zealand, grouped together. Both *Microcystis* strains from Meiktila Lake possessed similar 16S rRNA gene sequences and clustered together with 16S rRNA gene sequences of *Microcystis* from Europe, Asia, Africa and South America (Figure 4).

### 2.4. Identification of Cyanobacterial Toxins and Toxin-Producing Strains

Three of the five investigated *Raphidiopsis* strains produced CYNs in variable amounts by either enzyme-linked immunosorbent assay (ELISA) or liquid chromatography with tandem mass spectrometry (LC-MS/MS) (Table 3). Concentrations of CYNs were 1.8–4.3 µg mg^−1^ FW by ELISA. Using LC–MS/MS, CYN concentrations of 1.7–2.5 µg mg^−1^ FW and deoxyCYN from 1.3 to 7.3 µg mg^−1^ FW were detected. In the three CYN-producing strains, deoxyCYN comprised 43%–75% of the total CYNs.

All investigated *Raphidiopsis* strains tested negative for saxitoxins (STXs), anatoxins (ATXs) and MCs by ELISA. Both *Microcystis* strains tested negative for CYNs, STXs and ATXs by ELISA but were identified as MC-producers by ELISA and their MC profiles were therefore investigated by high resolution LC–MS/MS (LC–HRMS/MS).

**Figure 5:**
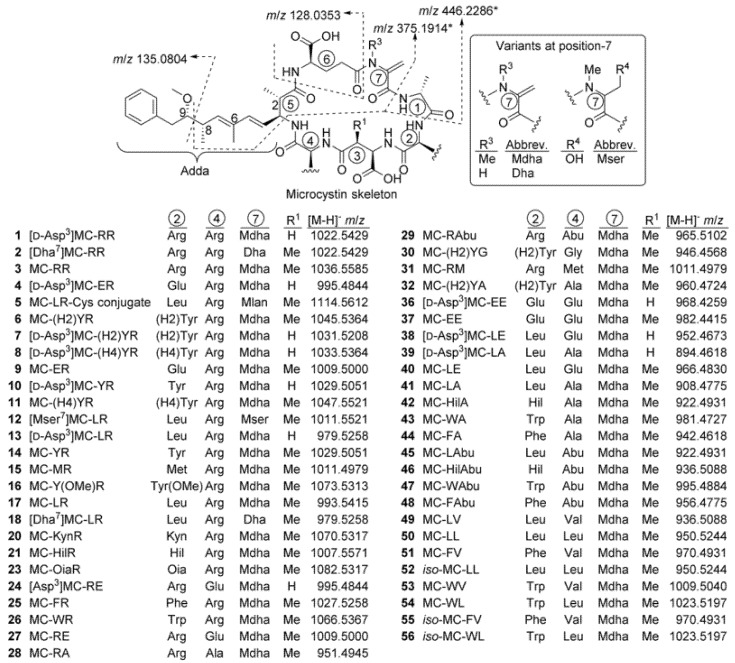
Structures and exact negative ionisation *m*/*z* of microcystins (MCs) identified in cultures AB2017/14 and AB2017/15 in this study, showing characteristic product ions at *m*/*z* 135.0804 (positive) and 128.0353 (negative) LC–MS/MS spectra (see Table 4 and Table S1). The origins of additional positive mode product ions containing Mdha^7^ (R^3^ = Me) at *m*/*z* 375.1914 and 446.2286 are also shown. Note that the corresponding product ions containing Dha^7^ (R^3^ = H) have *m*/*z* 361.1758 and 432.2129, and *m*/*z* 393.2020 and 464.2391 for Mser^7^ (R^4^ = OH). A full version of this table including positive ionisation data is shown in the Supporting Information (Table S1). Abbreviations: Abu, aminobutyric acid; Dha, dehydroalanine; (H2)Tyr, dihydrotyrosine; (H4)Tyr, 4,5,6,7-tetrahydrotyrosine; Kyn, kynurenine; Mdha, *N*-methyldehydroalanine; Mlan, *N*-methyllanthionine; Mser, *N*-methylserine; Oia, oxindolyalanine; Tyr(OMe), methoxytyrosine.

Underivatized samples were analysed by LC-HRMS/MS in positive and negative ionisation modes as previously described [12,13] and then after reaction with mercaptoethanol (targeting Mdha^7^/Dha^7^ moieties in MCs) [14] and Oxone/DMSO (targets sulfide groups in methionine and Cys/GSH conjugates of MCs) [15]. Results of these analyses are summarised in Figure 5, Table 4 and Table S1. Peaks from putative MCs were identified by their reaction with mercaptoethanol, production of characteristic product ions in data-dependent and/or data-independent acquisition (DDA and/or DIA) LC-MS/MS screens, and possessing plausible potential elemental formulae based on both positive and negative mode full scan HRMS. These peaks were then targeted by LC-HRMS/MS at suitable collision energies to obtain structurally informative HRMS/MS spectra to assist with identification, compared by LC–HRMS with samples containing some of the putative MCs, and subjected to selective oxidation to detect the presence of sulfide moieties that could be present in some of the MCs.

Peaks were only considered to be MCs if they: 1, showed apparent pseudo-molecular ions appropriate for a MC in both positive and negative ionisation modes; 2, showed one or more of the characteristic MC fragments shown in Figure 5; 3, displayed appropriate chemical reactivity for the putative structure, and; 4, displayed retention times (*t*_R_) and charge states (*z*) appropriate to the putative structure (e.g., based on the apparent number of polar and charged residues, such as Arg). MCs were considered “confirmed” (**1**, **3**, **13**, **14**, **17**, **18**, **21**, **25**, **26** and **41**) if they behaved identically in all respects to the standards (Table 4). Structures were considered “probable” if they behaved identically in all respects to a compound already identified with high probability in an available sample (**2**, **4**, **8**, **11**, **12**, **20**, **28**, **29**, **36**, **45** and **50**). For compounds for which standards or appropriate samples were not available, these were regarded as “probable” if, in addition to displaying the appropriate physical and chemical characteristics (Table 4 and Table S1), they also displayed interpretable MS/MS spectra that were clearly consistent with the proposed structure by comparison with related compounds (**5**–**7**, **9**, **10**, **15**, **16**, **24**, **27**, **30**–**32**, **37**–**40**, **42**–**44**, **46**–**49**, **51**, **53** and **54**). Compounds were considered tentative if there was limited MS/MS spectral evidence (**23**) or if the evidence was ambiguous (e.g., several isomers were present that showed indistinguishable MS/MS spectra, i.e., **52**, **55** and **56**). Compounds designated “unidentified” were definitively identified as MCs, but the spectral data was insufficient to identify them (**19**, **22** and **33**–**35**). All compounds listed in Table 4 as containing Mdha^7^ and which gave adequate signal-to-noise in their MS/MS spectra in positive mode, showed product ions at *m*/*z* 135.0804, 375.1914 and 446.2286, indicative of the presence of Adda^5^–D-Glu^6^–Mdha–D-Ala^1^, while in those listed as containing Mser^7^ or Dha^7^ the latter two product ions were heavier, or lighter, by a mass corresponding to H_2_O or CH_2_, respectively (all with Δ*m* < 5 ppm), and the presence of these units is implicit in the discussion of the structural elucidation in Section 3.

In culture AB2017/14, 52 microcystin congeners were detected by LC–HRMS, with a total concentration of 1100 µg g^−1^ FW. Twenty-one of these were unidentified or previously unreported variants. In culture AB2017/15, 20 microcystin variants (of which six were previously unreported) were detected, with a total concentration of 14000 µg g^−1^ FW. The microcystin variants and the concentrations found in each strain are shown in Table 4 and Table S1.

## 3. Discussion

This study clearly demonstrates for the first time the presence of CYN- and deoxyCYN-producing *R. raciborskii* and MC-producing *Microcystis* in the phytoplankton community of Meiktila Lake in Myanmar. The relatively high biomass of *R. raciborskii*, up to 1.9 mg L^−1^ in the phytoplankton community of Meiktila Lake, is expected to cause elevated concentrations of CYNs in the lake water. The results suggest a higher risk for humans and animals to be affected by CYNs than by MCs, although this could be affected by variations in the biomass of CYN-producing *R. raciborskii* versus MC-producing *Microcystis*. Variations in cyanobacterial bloom composition and toxin production are influenced by abiotic factors such as nutrients, temperature and light and by biotic factors such as grazing, parasitism and predation [16,17]. The distribution of CYN/deoxyCYN-producing and nonproducing *Raphidiopsis* strains in Meiktila Lake is likely to vary over time, and dominance by a *Raphidiopsis* strain such as AB2017/13 would lead to CYN and deoxyCYN concentrations up to 20 µg L^−1^ for the highest *Raphidiopsis* biomasses measured in this study. It is therefore expected that CYN/ deoxyCYN concentrations in the lake water will at times exceed the guideline value for CYN in drinking water of 1 µg L^−1^ [18]. The tolerable daily intake value of 0.03 µg kg^−1^ for a person of 70 kg body weight would be exceeded after the intake of slightly more than 100 mL of lake water if CYN and deoxyCYN are similarly toxic. However, the toxicity of deoxyCYN to humans is not yet clear. According to Norris et al. [19], deoxyCYN does not contribute significantly to the toxicity of *R. raciborskii*. In contrast, cell viability assays showed that deoxyCYN was only slightly less toxic than CYN and most likely operates by similar toxicological mechanisms [20]. The potential risk of deoxyCYN for humans needs therefore to be clarified [20]. The use of Meiktila Lake water for drinking water, irrigation, domestic purposes or animal consumption is complicated by the fact that an unknown proportion of CYNs can be extracellular and is therefore not eliminated by filtration. Lake water contaminated by CYN and other toxins like MC-LR can lead to morphological and physiological changes and potential loss of productivity by agricultural plants, and bioaccumulation of cyanotoxins in the tissues of edible terrestrial plants in a concentration-dependent manner has been reported [21].

Griffith and Saker [22] have shown that in stationary phase of cultures, more than 50% of CYN can be extracellular. In environmental samples, the same authors found that extracellular CYN could exceed 90%. Boiling in water does not significantly degrade CYN within 15 min [23]. The removal of extracellular CYN/deoxyCYN therefore needs other methods, like the use of activated carbon, membrane filtration or chemical inactivation (Ultraviolet (UV), or oxidants) [24]. The presence of *Raphidiopsis* strains in Meiktila Lake that do not produce CYN makes it likely that CYN concentrations in the lake will vary considerably depending on the ratio of the two chemotypes in the phytoplankton community. As Meiktila Lake water is used for domestic purposes (drinking water, irrigation, washing of clothes, and personal hygiene), regular monitoring of cyanobacterial biomass and CYNs is recommended.

*R. raciborskii* has not been described from Myanmar water bodies and was not observed in a phytoplankton community study conducted in Meiktila Lake from 2011 to 2012 [5]. *Raphidiopsis* (and *Cylindrospermopsis*) spp., however, have been described from various other Southeast Asian freshwater habitats [25,26,27] and other water habitats worldwide [8]. *R. raciborskii* is only known to produce CYNs in Australia and the Asian countries China, Japan, Vietnam and Thailand and to produce STXs in Brazil [26,28,29,30,31,32]. The prime radiation centre of *R. raciborskii* is thought to be in Africa, with a second radiation centre in Australia [8]. Our 16S rRNA gene analysis confirms the close relationship of the *Raphidiopsis* strains from Meiktila Lake to other *Raphidiopsis* strains from Asia, Europe and Australia. Our 16S rRNA gene tree also clearly supports the suggested movement of *Raphidiopsis* from the American continents to Southwest Europe and North Africa and probably further to Greece and China, as has been described [33,34]. The close relationship of *Raphidiopsis* strains from Australia, Asia and Europe does not, however, explain why CYN- and deoxyCYN-producing strains have only been found in Asia and Australia but not in Europe, Africa or the Americas. Parts of, or the whole, CYN gene cluster could have been lost during the spread from Asia westwards, or only nonCYN-producing strains may have spread to Europe. Our finding of nontoxic *Raphidiopsis* strains in Meiktila Lake supports the latter hypothesis.

**Figure 6:**
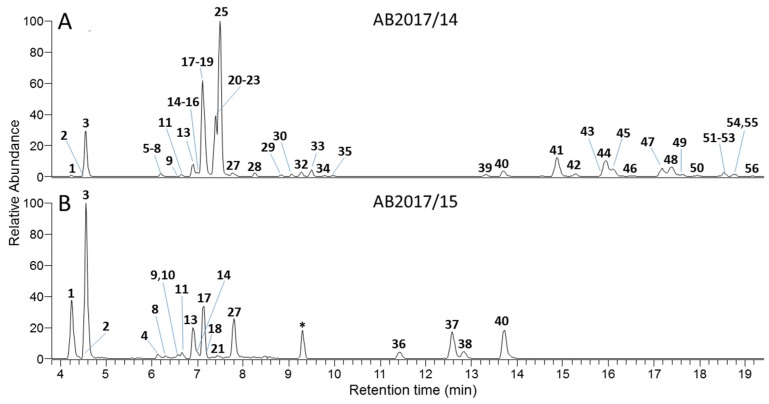
LC–HRMS full scan extracted ion chromatograms (3.8–19.4 min, positive ionization mode) of extracts from (**A**) *Microcystis* culture AB2017/14 and (**B**) *Microcystis* culture AB2017/15. Chromatograms were produced by extracting at *m*/*z* (± 5 ppm) for all MCs listed in Table 4 (see Table S1 for positive ionisation *m*/*z* values). Note that some of the smaller peaks are not labelled on the chromatograms, and the peak marked with an asterisk is not from a MC.

Both *Microcystis* strains AB 2017/14 and AB2017/15 isolated from Meiktila Lake are confirmed microcystin producers and are closely related to *Microcystis* strains from Africa, Europe and Asia based on 16S rRNA gene phylogeny. Both strains had identical 16S rRNA gene sequences but were clearly distinguished chemically by their MC congener profiles. Fifty-six microcystin variants were found in *Microcystis* strains AB2017/14 and AB2017/15 isolated from Meiktila Lake (Figure 6).

In order to reliably estimate the quantities of the MCs in the extracts by LC-HRMS, it was necessary to characterize, and if possible, identify all of them. The reason for this is that the response in LCMS can be expected to vary from congener-to-congener, primarily due to variations in the number of easily ionisable amino acid residues, especially Arg, present in the MC’s structure. Only the identification of previously unreported MCs (see Bouaïcha et al. [35]) in the cultures is discussed further (i.e., **7**, **9**, **19**, **22**, **24**, **27**, **30**–**35**, **37**, **38**, **40**, **42**, **46**, **51**–**53**, **55** and **56**) but spectra of all compounds for which adequate MS/MS spectra were obtained are available in the Supporting Information.

Three of the compounds were sulfide-containing variants (**5**, **15**, and **31**) which reacted when the extract was oxidised with Oxone/DMSO (Table 4). The first two have been reported in cultures and blooms [15,36], and their characteristics were fully consistent with those reported for **5** and **15** here, and in the case of **15** its oxidation product (MC-M(O)R) showed characteristic product ions including neutral loss of CH_4_OS and displayed an MS/MS spectrum (Figures S8, S11 and S14) identical to that reported previously for MC-M(O)R [15]. The third of sulfide-containing MC was identified as MC-RM (**31**) based on its physical and chemical properties (Table 4), which were essentially identical to those of **15** except for its longer *t*_R_ and that its MS/MS spectrum closely paralleled that of MC-RA (**28**) and displayed product ions characteristic of an MC with one Arg at position-2 rather than at the more common position-4 (Figures S8, S14 and S15). For example, fragments at *m*/*z* 440.2263 (C_18_H_30_O_6_N_7_^+^, Δ*m* = 2.2 ppm, from Mdha^7^–D-Ala^1^–Arg^2^–D-Masp^3^) and 731.3716 (C_34_H_51_O_10_N_8_^+^, Δ*m* = −0.9 ppm, from Adda^5^–D-Glu^6^–Mdha^7^–D-Ala^1^–Arg^2^–D-Masp^3^), together with the complete absence of a product ion at *m*/*z* 599.3552 (from Arg^4^–Adda^5^–D-Glu^6^), confirmed Arg at position-2 and Met at position-4 (see Okello et al. [37] for assigned product ions from MC-YR) of **31**.

Eight of the compounds (**4**, **9**, **24**, **27**, **36**–**38** and **40**) showed characteristics of MCs containing one or more Glu residues at position-2 or -4. Two of these (**9** and **27**) had formulae consistent with MC-RE or MC-ER (Table 4). Compound **9** gave product ions (Figures S2 and S5–S7) typical of a MC with Arg at position-4, including *m*/*z* 599.3536 (C_31_H_47_O_6_N_6_^+^, Δ*m* = −2.6 ppm, from Arg^4^–Adda^5^–D-Glu^6^), 284.1238 (C_12_H_18_O_5_N_3_^+^, Δ*m* = −1.1 ppm, from Mdha^7^–D-Ala^1^–Glu^2^), and 286.1497 (C_11_H_20_O_4_N_5_^+^, Δ*m* = −4.3 ppm, from D-Masp^3^–Arg^4^), indicating **9** to be MC-ER. The MS/MS spectrum of **27** (Figures S2 and S15) included product ions at *m*/*z* 440.2248 (C_18_H_30_O_6_N_7_^+^, Δ*m* = −0.9 ppm, from Mdha^7^–D-Ala^1^–Arg^2^–D-Masp^3^), and 731.3678 (C_34_H_51_O_10_N_8_^+^, Δ*m* = −6.1 ppm, from Adda^5^–D-Glu^6^–Mdha^7^–D-Ala^1^–Arg^2^–D-Masp^3^) which, together with the complete absence of a product ion at *m*/*z* 599.3552, confirmed Arg at position-2 and Glu at position-4 (see Okello et al. [37]) for assigned product ions from MC-RY), showing **27** to be MC-RE. The characteristics of **37** (Table 4) were consistent with MC-EE. In addition, **37** gave product ions (Figures S27–S33) including *m*/*z* 276.1189 (C_10_H_18_O_6_N_3_^+^, Δ*m* = −0.4 ppm, from D-Masp^3^–Glu^4^), 405.1605 (C_15_H_25_O_9_N_4_^+^, Δ*m* = −2.9. ppm, from Glu^2^–D-Masp^3^–Glu^4^) and 575.2703 (C_28_H_39_O_9_N_4_^+^, Δ*m* = −1.5 ppm, from Adda^5^–D-Glu^6^–Mdha^7^–D-Ala^1^–Glu^2^) that confirmed **37** as MC-EE. Compound **40** was identified as MC-LE based on the characteristics presented in Table 4, as well as product ions (Figures S27–S33) observed in its MS/MS spectra, including *m*/*z* 460.2397 (C_19_H_34_N_5_O_8_^+^, Δ*m* = −1.2, from D-Ala^1^–Leu^2^-D-Masp^3^–Glu^4^) and 397.2073 (C_18_H_29_N_4_O_6_^+^, Δ*m* = −2.2, from Mdha^7^–D-Ala^1^–Leu^2^-D-Masp^3^), confirmed its identity as MC-LE (**40**). Earlier-eluting desmethylated D-variants of **9**, **27**, **37** and **40** were similarly identified as the corresponding D-Asp^3^-congeners [D-Asp^3^]MC-ER (**4**), [D-Asp^3^]MC-RE (**24**), [D-Asp^3^]MC-EE (**36**) and [D-Asp^3^]MC-LE (**38**) based on analysis of their MS/MS spectra (Figures S21–S27) and characteristics presented in Table 4. Furthermore, the **4** and **36** in this sample coeluted with, and gave identical product ion spectra to, [D-Asp^3^]MC-ER (**4**) and [D-Asp^3^]MC-EE (**36**) identified [12] in an extract of a culture of *Planktothrix prolifica* NIVA-CYA544.

Twenty-one conventional late-eluting nonArg-containing MCs (**30**, **32** and **36**–**56**) were detected. Of these, the identities of four that contained Glu^2^ or Glu^4^ (**36**–**38** and **40**) were discussed above. The remaining previously unreported nonArg MCs were **30**, **32**, **42**, **46**, **51**–**53**, **55** and **56**. Compound **42** had the same characteristics as MC-LAbu (**45**) (Table 4), however, its MS/MS spectrum (Figures S34–S36) was consistent with MC-HilA. In particular, product ions at *m*/*z* 573.3270 (C_30_H_45_N_4_O_7_^+^, Δ*m* = −2.3 ppm, from Adda^5^–D-Glu^6^–Mdha^7^–D-Ala^1^–Hil^2^ minus C_9_H_10_O (cf *m*/*z* 559.3126 for **41** and **45**)) and 411.2231 (C_19_H_31_N_4_O_6_^+^, Δ*m* = −1.6 ppm, from Mdha^7^–D-Ala^1^–Hil^2^-D-Masp^3^ (cf *m*/*z* 397.2082 for **41** and **45**)) as well as a range of other ions indicated the identity as MC-HilA (**42**), although the actual connectivity of the carbons in the amino acid side-chain at position-2 cannot be determined by mass spectrometry. A related compound (**46**) had characteristics (Table 4) and gave product ions (Figures S40–S42) that were consistent with MC-HilAbu. Product ions included *m*/*z* 573.3260 (C_30_H_45_N_4_O_7_^+^, Δ*m* = −3.9, from Adda^5^–D-Glu^6^–Mdha^7^–D-Ala^1^–Hil^2^ minus C_9_H_10_O), 232.1291 (C_9_H_18_N_3_O_4_^+^, Δ*m* = −0.4, from D-Masp^3^–Abu^4^) and 430.2651 (C_19_H_36_N_5_O_6_^+^, Δ*m* = −2.1, from D-Ala^1^–Hil^2^-D-Masp^3^–Abu^4^ (cf *m*/*z* 402.2347 for **41** and 416.2504 **42**). This data unambiguously shows the presence of an extra CH_2_ group in both amino acid-2 and -4 in **46**, relative to MC-LA (**41**) and is consistent with MC-HilAbu (**46**). Compound **51** displayed characteristics consistent with MC-FV (Table 4), as well as product ions (Figures S49–S51) at *m*/*z* 246.1456 (C_10_H_20_N_3_O_4_^+^, Δ*m* = −0.4, from D-Masp^3^–Val^4^), 593.2960 (C_32_H_41_N_4_O_7_^+^, Δ*m* = −1.6, from Adda^5^–D-Glu^6^–Mdha^7^–D-Ala^1^–Phe^2^ minus C_9_H_10_O (cf. 559.3126 for **41**)) and 464.2494 (C_22_H_34_N_5_O_6_^+^, Δ*m* = −3.1, from D-Ala^1^–Phe^2^ –D-Masp^3^–Val^4^ (cf. 402.2347 for **41**)). This establishes an extra C_5_H_2_ and 4 RDBE in amino acid-2 and C_2_H_4_ in amino acid-4, relative to MC-LA (**41**), consistent with MC-FV (**51**). Compound **53** had characteristics consistent with MC-WV (Table 4). This was supported by its MS/MS spectra (Figures S49–S51), which included product ions at *m*/*z* 246.1456 (C_10_H_20_N_3_O_4_^+^, Δ*m* = 2.9, from D-Masp^3^–Val^4^), 632.3063 (C_34_H_42_N_5_O_7_^+^, Δ*m* = −2.5, from Adda^5^–D-Glu^6^–Mdha^7^–D-Ala^1^–Trp^2^ minus C_9_H_10_O (cf. 559.3126 for **41**)) and 503.2605 (C_24_H_35_N_6_O_6_^+^, Δ*m* = −2.6, from D-Ala^1^–Trp^2^–D-Masp^3^–Val^4^ (cf. 402.2347 for **41**)). This indicates the presence of an extra C_5_HN and 6 RDBE in amino acid-2 and C_2_H_4_ in amino acid-4, relative to MC-LA (**41**), consistent with MC-WV (53). Later-eluting isomers of **50**, **51** and **54** were also present (i.e., **52**, **55** and **56**), with identical characteristics (Table 5) and product ion spectra (Figures S52–S56). These compounds all contain branching amino acids at the variable position-2 (nominally Leu for **52** and **56**) or -4 (Val for **55**), and most likely the isomers present result from changes to this branching (e.g., Ile or 2-aminohexanoic acid at position-2, and 2-aminopentanoic acid or isovaline at position-4).

In addition, **30** and **32** differed from each other by CH_2_ and had characteristics consistent with MC-(H2)YA (**32**) and MC-(H2)YG (**30**), respectively (Table 4). These fragmented somewhat differently from typical Arg-free MCs such as MC-LA (**41**) (Figure S17). Both compounds showed weak product ions at *m*/*z* 155.0815 and 580.3017, indicating that **30** and **32** both contained Adda^5^–D-Glu^6^–Mdha^7^–D-Ala^1^. However, both **30** and **32** also gave product ions at *m*/*z* 320.1605, 611.3075 and 745.3807 (cf. 268.1650, 559.3117 and 693.3854 in MC-LA (**41**)), indicating the presence of an extra C_3_O and 3 RDBE at amino acid-2 relative to **41**, consistent with the presence of the unusual amino acid L-dihydrotyrosine ((H2)Y) at position-2. Compounds **30** and **32** gave product ions as *m*/*z* 449.2033 and 449.2015 (C_21_H_29_N_4_O_7_^+^, Δ 0.5 and −3.6 ppm, respectively, from Mdha^7^–D-Ala^1^–(H2)Tyr^2^–D-Masp^3^; cf. *m*/*z* 397.2082 for **41**, from Mdha^7^–D-Ala^1^–Leu^2^–D-Masp^3^). Thus, the difference in mass (14.0157, i.e., CH_2_) between **30** and **32** lies not in residue-3 (D-Masp^3^ vs D-Asp^3^) as might be expected but in residue-4. Thus, **32** is identified as MC-(H2)YA, and **30** as MC-(H2)YG, which appears to be the first MC so far reported [35] with Gly at position-4.

Compounds **6** and **7** coeluted but differed by a mass corresponding to CH_2_, with **6** having the same accurate mass, MS/MS spectrum (Figure S3) and retention time as MC-(H2)YR (**6**) identified in a sample from a recent study [13]. The MS of **7** was consistent with [D-Asp^3^]MC-(H2)YR, and the MS/MS spectra of **6** and **7** were very similar. The MS/MS spectrum of **7** included product ions (Figure S4) at *m*/*z* 155.0813 (Δ*m* = −1.6 ppm, from Mdha^7^–D-Ala^1^) and 599.3525 (C_31_H_47_O_6_N_6_^+^, Δ*m* = −4.5 ppm, from Arg^4^–Adda^5^–D-Glu^6^), 120.0806 (C_8_H_10_N^+^, Δ*m* = −1.6 ppm, from (H2)Tyr), and 320.1611 (C_16_H_22_O_4_N_3_^+^, Δ*m* = 2.0 ppm, from Mdha^7^–D-Ala^1^–(H2)Tyr^2^), 272.1353 (C_10_H_18_O_4_N_5_^+^, Δ*m* = 2.0 ppm, from D-Asp^3^–Arg^4^) and 714.3802 (C_35_H_52_O_9_N_7_^+^, Δ*m* = −2.7 ppm, from D-Asp^3^–Arg^4^–Adda^5^–D-Glu^6^), showing that **7** is [D-Asp^3^]MC-(H2)YR.

Five MCs were present (**19**, **22** and **33**–**35**) whose structures could not be identified from their characteristics (Table 4 and Table S1) or product ion spectra (Figures S12 and S18–S20).Compounds **33** and **34** gave apparent *m*/*z* values that did not correspond to known or plausible MC variants and differed from each other by a mass corresponding to CH_2_. They did not contain Arg but contained one extra nitrogen atom, 5 extra RDBE and, more surprisingly, one less oxygen atom than MC-LA (**41**). Compound **33** gave product ions at *m*/*z* 375.1911 (C_20_H_27_O_5_N_2_^+^, Δ*m* = −0.9 ppm, from Adda^5^–D-Glu^6^–Mdha^7^ minus C_9_H_10_O), 446.2284 (C_23_H_32_N_3_O_6_^+^, Δ*m* = −0.4 ppm, from Adda^5^–D-Glu^6^–Mdha^7^–D-Ala^1^ minus C_9_H_10_O) and 580.2986 (C_32_H_42_N_3_O_7_^+^, Δ*m* = −5.4 ppm, from Adda^5^–D-Glu^6^–Mdha^7^–D-Ala^1^), however, fragments containing amino acids 2–4 were either shifted or absent. Although the data appear to be consistent with analogues containing Orn and Phe at positions 2 and 4 with an amide linkage to the neighbouring D-Asp^3^/Masp^3^ residue, further data are required for even a tentative structural assignment. The apparent pseudomolecular ion isotope envelopes of **33** and **34** (Figure S60) displayed unusual patterns that suggest that these compounds may be reacting during ionisation, possibly including dehydration, further complicating mass spectral analysis.

As with **30** and **32**, compound **35** showed product ions at *m*/*z* 213.0866, 320.1583, 375.1909, 446.2279, 449.2013, 509.2680 and 611.3040, consistent with the presence of Adda^5^–D-Glu^6^–Mdha^7^–D-Ala^1^–(H2)Tyr^2^–D-Masp^3^, which would require an MC with an amino acid side-chain at position-4 possessing an unprecedented C_3_H_3_ and 2 RDBE. This could possibly be due to the presence of a larger fragile amino acid at this position that undergoes ready elimination during MS, and the full structure of **35** remains undetermined.

Compounds **19** and **22** had identical product ion spectra including ions at *m/z* 135.0803, 213.0866, 269.1235, 375.1907, 446.2281, 599.3533, 640.2807 and 710.3886, identical to those from MC-LR, indicating the presence of D-Masp^3^–Arg^4^–Adda^5^–D-Glu^6^–Mdha^7^–D-Ala^1^ and that these compounds differed from MC-LR (**17**) and MC-WR (**26**) only in the amino acid at position-2. Product ions at 469.1815 and 486.2111 (both from X^2^–D-Masp^3^–Arg^4^) and 173.0709 and 144.0455 (from X^2^ and 173.0709−CH_3_N) were consistent with this and indicated the presence of a side chain at position-2 containing C_9_H_6_NO and 7 RDBE (cf. C_9_H_8_N and 6 RDBE for the side chain of Trp in **26**), suggesting the presence of an unidentified oxidised variant of Trp present in the two isomeric MCs, **19** and **22**.

Between them, the two cultures contained 34 known and 22 previously unreported MCs of which 14 were assigned probable structures based on their chemical and mass spectrometric properties. In all cases, the number of Arg residues present in the MCs were reliably determined from their elemental compositions and charge-state, even for congeners for which definitive structures could not be established. Quantitation of the individual congeners was then performed from negative (Table 4) and positive mode full scan chromatograms, which gave essentially identical results, relative to 3-point calibration curves of the appropriate MC reference materials (RMs) containing no, one or two Arg residues ([D-Leu^1^]MC-LY [38], MC-LR (**17**) and MC-RR (**3**), respectively). AB2017/14 contained 52 MCs, of which only 31 had been previously reported, and the newly reported MCs constituted nearly 20% of the total identified MC content. AB2017/15 contained fewer MCs (20) of which only six had not been reported previously, but these constituted nearly 34% of the total MC content in this culture. Notable amongst the newly reported MCs are those containing Glu at positions 2 and 4 (**4**, **9**, **24**, **27**, **36**–**38**, and **40**), which constituted 1.7% of the MCs in AB2017/14 and 37.5% of the MCs in AB2017/15. MCs of this type have only been reported previously from two sources [12,39]. AB2017/14 also contained a number of congeners that appeared to be derived from MC-WR (**19**, **20**, **22**, **23** and **26**) that together constituted 20.2% of its MC content, and an unusually high number (25) of nonArg-containing MCs (**30**, **32**–**35** and **37**–**56**) together constituting 20.5% of the total MC content. These results underscore both the diversity of MCs that may be present in a single sample and the potential difficulty of reliably quantitating the total MCs using traditional targeted LC–MS/MS methods. These factors may contribute to the reported apparent overestimation of MC levels when using less-targeted methods such as ELISA and PP2A inhibition, relative to highly congener-targeted LC–MS/MS approaches [40].

Although both strains produce a variety of MC variants, the risk of harmful effects caused by microcystins is likely to be low due to the low *Microcystis* biomasses observed in Meiktila Lake during this study. An increase in *Microcystis* biomass cannot, however, be excluded due to the pollution from various sources, e.g., waste water and street runoff [6]. This will most likely lead to an increase in microcystin concentrations in the lake, with potential harmful effects on humans, domestic and wild animals using the untreated lake water. The toxicity of microcystins found in the strains isolated from Meiktila Lake varies from highly toxic variants like MC-LR or MC-LA to less toxic variants such as MC-RR [41]. The toxicity of most of the MC variants found in this study has not yet been described, which makes a full risk assessment difficult. The production of a high number of MC variants (up to 47) has also been shown for two other *Microcystis* strains isolated from South African Haartbeestpoort Dam and Japanese Lake Kasumigaura [42,43].

Shallow lakes like Meiktila Lake are often characterized by competition between macrophytes and phytoplankton. High nutrient loading and phytoplankton growth lead to turbid water conditions and prevent the growth of macrophytes [44]. In Meiktila Lake the present turbid conditions are explained by deforestation and erosion in the catchment area, pollution with domestic waste water, street runoff and solid waste [6]. The relatively strong growth of *R. raciborskii* most likely is an additional reason for the observed turbidity at sampling points MK1, MK2, MK4 and MK5 in Meiktila Lake. The *Potamogeton* belt which separates MK2 and MK3 seems to act as a kind of filter or barrier because the Secchi depth at MK3 (1.8 m) was considerably higher than at MK1 (0.8 m). According to Van Donk and Van de Bund [45], macrophytes significantly modify the composition of the phytoplankton community and lead to a decrease in its abundance and biomass. It has been shown that certain macrophyte species exhibit allelopathic activity against certain phytoplankton species [46]. The obvious decrease of *Raphidiopsis* biomass from MK2 to MK3 may be therefore attributable to allelopathic effects of the macrophyte species in the belt. Allelopathy has been suggested by several authors to be responsible for observed phytoplankton patterns in whole-lake studies of vegetated, shallow lakes, but evidence for or against allelopathy has not been provided [47,48,49].

## 4. Conclusions

In conclusion, this is the first report of *R. raciborskii* and *Microcystis* in Meiktila Lake, Myanmar. Three of five *Raphidiopsis* isolates produced CYN and deoxyCYN, like other *Raphidiopsis*/*Cylindrospermopsis* isolates described from other Asian countries and Australia. Both *Microcystis* strains isolated from Meiktila Lake produced at least 56 MC variants (52 for AB2017/14 and 20 for AB2017/15), including 22 previously undescribed congeners. Harmful effects on humans and animals using Meiktila Lake as a water source cannot therefore be excluded.

## 5. Materials and Methods

### 5.1. Study Area, Measurements and Sampling

Meiktila Lake is a shallow reservoir, located close to Meiktila city in central Myanmar in the Mandalay region at an altitude of 230 m (Figure 1). During the rainy season, from April/May to October/November, it receives water from Mondaing Dam, located ca. 15 km west of Meiktila at an altitude of 245 m. Depending on the season, Meiktila Lake covers an area of around 54 km^2^ with a maximum water depth of 10 m. It is divided by a dam into a northern and a southern lake [5]. Five sampling points were selected, three in the northern part of Meiktila Lake and two in the southern part (Figure 1, Table 5).

Sampling points 2 and 3 in the Northern Part of Meiktila Lake were separated by a broad *Potamogeton* belt. At all five sampling points in March and November 2017, in situ measurements of water temperature, pH, conductivity and dissolved oxygen were conducted, and integrated water samples were taken (1 m steps up to max. 3 m water depth) for the analysis of chemical parameters (ammonium, nitrate, total nitrogen, soluble reactive phosphorous, total phosphorous, Ca, phytoplankton composition and biomass and for the isolation of cyanobacterial strains. For quantitative phytoplankton analysis, a 50 mL subsample was removed from a sample taken from integrated samples and preserved with Lugol’s solution and a concentrated net sample (mesh size 20 µm) was taken and preserved by addition of formaldehyde (4% final concentration) for qualitative analysis. A 50 mL water sample for isolation of cyanobacteria was taken at each sampling point and kept in a cool shady place and gently shaken twice per day before further treatment in Norway.

### 5.2. Phytoplankton Analysis

The Lugol-fixed phytoplankton samples were counted in sedimentation chambers (Hydro-Bios Apparatebau GmbH Kiel, Germany) using an inverted microscope (Leica DMi8; Ortomedic, Oslo, Norway) according to Utermöhl [50]. Phytoplankton biomass was calculated by geometrical approximations using the computerized counting programme Opticount (SequentiX - Digital DNA Processing, Klein Raden, Germany). The specific density of phytoplankton cells was calculated as 1 g cm^−3^.

### 5.3. Isolation of Strains and Morphological Characterization

Using a microcapillary, single colonies of *Microcystis* and filaments of *Raphidiopsis* were isolated. They were washed five times and placed in wells on microtiter plates containing 300 µL Z8 medium [51]. After successful growth, the samples were placed in 50 mL Erlenmeyer flasks containing 20 mL Z8 medium and maintained at 22 °C. Strains were classified based on morphological traits [52,53]. Morphological examination was conducted using a Leica DM2500 light microscope, Leica DFC450 camera and Leica Application Suite software (LAS) (Leica, Oslo, Norway). The morphological identification was based on the following criteria: (i) size of vegetative cells and heterocytes and (ii) nature and shape of filaments or colonies. Length and width of 50–250 vegetative cells or filaments and of 20–50 heterocytes were measured. Akinetes were not detected in the samples. All strains used in this study (Table 2) are maintained at the Norwegian Institute for Water Research, Oslo, Norway.

### 5.4. Genomic DNA Extraction, PCR Amplification and Sequencing

Genomic DNA was extracted according to Ballot et al. [54]. All PCRs were performed on a Bio-Rad CFX96 Real-Time PCR Detection System (Bio-Rad Laboratories, Oslo, Norway) using the iProof High-Fidelity PCR Kit (Bio-Rad Laboratories, Oslo, Norway). The 16S rRNA gene of the isolated strains from Meiktila Lake was amplified using the primers as described by Ballot et al. [54]. PCR products were visualized by 1% agarose gel electrophoresis with GelRed staining (GelRed Nucleic Acid Gel Stain, Biotium, Fremont, CA, USA) and UV illumination.

Amplified 16S rRNA gene products were purified through Qiaquick PCR purification columns (Qiagen, Hilden, Germany). Sequencing of the purified 16S rRNA gene products was performed using the same primers as for PCR and intermediate primers as described in Ballot et al. [54]. For each PCR product, both strands were sequenced on an ABI 3730 Avant genetic analyser using the BigDye terminator V.3.1 cycle sequencing kit (Applied Biosystems, Thermo Fisher Scientific Oslo, Norway) according to the manufacturer’s instructions.

### 5.5. Phylogenetic Analysis

Sequences of the 16S rRNA gene of the cyanobacterial strains were analysed using the Seqassem software package (version 07/2008) and the Align MS Windows-based manual sequence alignment editor (version 03/2007) (SequentiX - Digital DNA Processing, Klein Raden, Germany). Segments with highly variable and ambiguous regions and gaps making proper alignment impossible were excluded from the analyses.

A 16S rRNA gene set containing 1135 positions was used in the phylogenetic tree for *Cylindrospermopsis*/*Raphidiopsis*. *Sphaerospermopsis aphanizomenoides* (LN846954) was employed as the outgroup, five *Raphidiopsis* strains from Meiktila Lake and 35 additional *Cylindrospermopsis*/*Raphidiopsis* sequences derived from GenBank were included in the analyses. A set containing 1426 positions was used for the *Microcystis* 16S rRNA gene analysis*. Chroococcus subviolaceus* (MF072353) was employed as the outgroup, two strains from Meiktila Lake and 38 additional *Microcystis* sequences derived from GenBank were included in the analysis. Phylogenetic trees for 16S rRNA genes were constructed using the ML algorithm in Mega v. 7 [55]. In the ML analyses, evolutionary substitution models were evaluated using Mega v. 7 [55]. The HKY+G+I evolutionary model was found to be the best-fitting evolutionary model for the Nostocales 16S rRNA gene tree and T92+G+I for the *Microcystis* 16S rRNA gene tree. ML analyses of both trees were performed with 1000 bootstrap replicates using Mega v. 7 [55]. The sequence data were submitted to the European Nucleotide Archive (ENA) under the accession numbers listed in Table 2.

### 5.6. Toxin Analysis

#### 5.6.1. Material

LC–MS/MS utilised standards of ATX-a (Tocris Bioscience, Bristol, UK), homoATX-a (Novakits, Nantes, France) and CYN (Vinci Biochem, Vinci, Italy) and certified reference materials (CRMs) of STX, dcSTX, NeoSTX, GTX1, GTX4, GTX5 and C1 and C2 toxins (National Research Council of Canada, Halifax, NS, Canada (NRC)). LC–HRMS utilised CRMs of MC-RR (**3**), MC-LR (**17**) and [Dha^7^]MC-LR (**18**) (NRC) and an RM of [D-Leu^1^]MC-LY [38]. Additional RMs of [D-Asp^3^]MC-RR (**1**), D-Asp^3^]MC-LR (**13**), MC-YR (**14**), MC-HilR (**21**) containing traces of MC-FR (**25**), MC-WR (**26**) and MC-LA (**41**) were prepared at NRC from commercial samples (Enzo Life Sciences, Farmingdale, NY, USA), and extracts containing an array of other identified MCs were available from previous work [12,13,15]. Standards for the Adda-ELISA and for the CYN, ATX and STX ELISAs were as provided with the kits (Abraxis LLC, Warminister, PA, USA).

#### 5.6.2. ELISA for MCs, CYNs, ATXs and STXs

Fresh culture material of two *Microcystis* and five *Raphidiopsis* strains was frozen and thawed three times. The *R. raciborskii* strains were tested for CYNs using the Abraxis Cylindrospermopsin ELISA kit (Abraxis LLC, Warminister, PA, USA) following the manufacturer’s instructions. The test is a direct competitive ELISA that detects cylindrospermopsin but also recognizes deoxycylindrospermopsin and 7-*epi*-cylindrospermopsin. The ELISA results do not distinguish between dissolved and cell-bound toxins. Both *Microcystis* strains were tested for microcystins using the Abraxis Microcystins/Nodularins (ADDA) ELISA kits (Abraxis LLC, Warminister, PA, USA). The test is an indirect competitive ELISA designed to detect Adda, (3-amino-9-methoxy-2,6,8-trimethyl-10-phenyldeca-4,6-dienoic acid), based on specific recognition of the Adda moiety [56]. ADDA is a nonprotein amino acid and is the most common side chain at position-5 in microcystins (Figure 5).

All strains were also tested for saxitoxins and anatoxin-a using the Abraxis Saxitoxins (PSP) and Abraxis Anatoxin (VFDF) ELISA kits (Abraxis LLC, Warminister, PA, USA). The saxitoxin ELISA is a direct competitive ELISA that detects saxitoxin based on specific antibody recognition but also recognizes other saxitoxins (e.g., dcSTX, GTXs, lyngbyatoxin, NeoSTX) to varying degrees according to the manufacturer’s instructions. The test for anatoxin-a is a direct competitive ELISA that detects anatoxin-a based on specific antibody recognition but also recognizes homoanatoxin according to the manufacturer’s instructions The colour reaction of all ELISA tests was evaluated at 450 nm on a Perkin Elmer1420 Multilabel counter Victor3 (Perkin Elmer, Waltham, MA, USA), and concentrations were evaluated by manual analysis of the absorbance data as recommended by the vendor.

#### 5.6.3. Microcystin Analysis by LC–HRMS

Fresh culture material of both *Microcystis* strains was prepared for LC–HRMS by freeze-thawing (3 times), diluting with an equal volume of MeOH and filtering (0.22 μm) [57]). LC–HRMS/MS analysis was performed on a Q Exactive-HF Orbitrap mass spectrometer equipped with a HESI-II heated electrospray ionization interface (ThermoFisher Scientific, Waltham, MA, USA) using an Agilent 1200 LC system including a binary pump, autosampler and column oven (Agilent, Santa Clara, CA, USA). Analyses were performed with SymmetryShield 3.5 µm C18 column (100 × 2.1 mm; Waters, Milford, MA, USA) held at 40 °C with mobile phases A and B of H_2_O and CH_3_CN, respectively, each of which contained formic acid (0.1% v/v). Gradient elution (0.3 mL min^−1^) was from 20% to 90% B over 18 min, then to 100% B over 0.1 min and a hold at 100% B (2.9 min), then returned to 20% B over 0.1 min with a hold at 20% B (3.9 min) to equilibrate the column (total run time 25 min). Injection volume was typically 1–5 µL.

The MS was operated in positive ion mode and calibrated from *m*/*z* 74 to 1622. The spray voltage was 3.7 kV, the capillary temperature was 350 °C and the sheath and auxiliary gas flow rates were 25 and 8 units, respectively, with MS data acquired from 2 to 20 min. Mass spectral data were collected using a combined full scan (FS) and data independent acquisition (DIA) method. FS data was collected from *m*/*z* 500 to 1400 using the 60,000-resolution setting, an AGC target of 1 × 10^6^ and a max IT of 100 ms. DIA data was collected using the 15,000 resolution setting, an AGC target of 2 × 10^5^, maxIT set to “auto” and a stepped collision energy of 30, 60 and 80 eV. Precursor isolation windows were 62 *m*/*z* wide with centering at *m*/*z* 530, 590, 650, 710, 770, 830, 890, 950, 1010, 1070, 1130, 1190, 1250, 1310 and 1370. Mass spectral data were also collected using a combined full scan (FS) and top-10 data-dependent acquisition (DDA) method. Data was acquired as for DIA but with an exclusion list generated from a blank injection and an inclusion list (both at ± 5 ppm) from a publicly available database of MC *m*/*z* values [58], except that maxIT was set to 100 ms and dynamic exclusion 5.0 s and “if idle pick others” were selected. Putative MCs detected using the above FS/DIA method were further probed in a targeted manner using the parallel reaction monitoring scan (PRM) mode with a 0.7 *m*/*z* precursor isolation window, typically using the 30,000-resolution setting, an AGC target of 5 × 10^5^ and a max IT of 400 ms. Typical collision energies were: stepped CE at 30 and 35 eV for MCs with no Arg; stepped CE at 60, 65 and 70 eV for MCs with one Arg; and CE at 65 eV for [M+H]^+^ and stepped CE at 20, 25 and 30 eV for [M+2H]^2+^ of MCs with two Arg groups. Full scan chromatograms were obtained in MS-SIM mode as for DIA but with resolution 120,000 and max IT 300 ms.

In negative mode, the mass spectrometer was calibrated from *m*/*z* 69 to 1780 and the spray voltage was −3.7 kV, while the capillary temperature, sheath and auxiliary gas flow rates were the same as for positive mode. Mass spectrometry data were collected in FS/DIA scan mode as above using a scan range of *m*/*z* 750–1400, a resolution setting of 60,000, AGC target of 1 × 10^6^ and a max IT of 100 ms. For DIA, MS/MS data was collected from *m*/*z* 93 to 1400 using a resolution setting of 15,000, AGC target of 2 × 10^5^, max IT set to “auto” and stepped collision energy 65 and 100 eV. Isolation windows were 45 *m*/*z* wide and centered at *m*/*z* 772, 815, 858, 902, 945, 988, 1032, 1075, 1118, 1162, 1205, 1248, 1294, 1335 and 1378. Mass spectral data were also collected using a combined full scan (FS) and top-10 data dependent acquisition (DDA) method. Data were acquired as for DIA but with an exclusion list generated from a blank injection and an inclusion list from a publicly available list (both at ± 5 ppm) of MC *m*/*z* values [58], except that maxIT was set to 100 ms and dynamic exclusion 5.0 s and “if idle pick others” were selected. Full scan chromatograms were obtained over a scan range *m*/*z* 750–1400 at a resolution setting of 120,000 using an AGC target of 1 × 10^6^ and a max IT of 300 ms.

Thiol derivatizations were performed by addition of (NH_4_)_2_CO_3_ (0.1 M, 200 µL) to the filtered extract (200 µL), with 200 µL transferred to two LC-MS vials. To one vial was added 1 µL of a 1:1 mixture of mercaptoethanol and *d*_4_-mercaptoethanol (Sigma–Aldrich, St. Louis, MO, USA), while 1 µL of water was added to the other vial as a control. Oxidations were performed by addition of DMSO (5 µL) and Oxone (10 mg/mL in water; 25 µL) to 50 µL of extract [15]. Samples and reactions were placed in the sample tray (held at 15 °C) for analysis, and the reactions were monitored periodically until completion and then analysed.

#### 5.6.4. CYN, deoxyCYN, ATXs and STXs Analysis by LC-MS/MS

Extraction was performed on freeze-dried cultures (40 mL), according to the protocol in [59]. In brief, dry material was treated with 6 mL of 50% methanol and sonicated (Omniruptor4000 probe sonicator, Omni-Inc., Kennesaw, MA, USA) for 10 min in pulsed mode (50%) using 160 W power. An aliquot of the solution was then filtered on Phenex RC syringe filters (0.2 μm; Phenomenex, Castel Maggiore, Italy) and analyzed by LC–MS/MS.

LC-MS/MS analysis were performed using a Waters Acquity UPLC system (Waters, Milford, MA, USA) coupled to a SCIEX 4000 QTRAP mass spectrometer (AB Sciex Pte. Ltd., Singapore). Chromatographic separation of analytes was performed using a HILIC column (Ascentis Express OH5, 2.7 μm, 50 × 2.1 mm; Merck Life Science S.r.l., Milan, Italy), while MS detection was performed using positive electrospray ionization using scheduled Multiple Reaction Monitoring. Details of the experimental set up are as described by Cerasino et al. [60], and the method was suitable for the detection and quantification of the following toxins: ATX-a, homoATX-a, CYN, STX, dcSTX, NeoSTX, GTX1, GTX4, GTX5 and C1 and C2 [60]. Quantification limits were 0.2–200 µg L^–1^. Other toxic alkaloids not available as pure standards were also screened but only for tentative analysis (hydroxy-, epoxy- and homo-ATXs, deoxyCYN, dcNeoSTX, GTX2/3, dcGTX2/3 and C3 and C4 toxins) using equivalent detection settings to their most similar analogs.

## Figures and Tables

**Table 1:** Biomass (mg L^−1^ FW) of phytoplankton groups at sampling points MK1–MK5 in Meiktila Lake in March and November of 2017.

Sampling Point	MK1	MK 1	MK 2	MK 2	MK 3	MK 3	MK 4	MK 4	MK 5	MK 5
Sampling Date	Mar	Nov	Mar	Nov	Mar	Nov	Mar	Nov	Mar	Nov
Phytoplankton Group										
Bacillariophyceae	0.525	0.149	1.316	0.101	0.035	0.301	0.083	0.064	0.088	0.059
Chlorophyceae	0.143	0.055	0.182	0.146	0.102	0.045	0.101	0.102	0.107	0.165
Chrysophyceae	0.011	0	0.002	0	0.045	0.021	0.006	0.007	0	0.004
Conjugatophyceae	0.099	0.006	0.141	0	0.013	0	0.008	0.017	0.045	0
Cryptophyceae	0.023	0.132	0.007	0.170	0.155	0.299	0.127	0.275	0.106	0.138
Cyanobacteria	1.063	1.965	1.640	1.050	0.606	0.078	1.064	1.990	0.751	2.397
Dinophyceae	0.186	0.005	0.222	0	0.285	0	0.084	0.006	0.078	0
Euglenophyceae	0.255	0.029	0.159	0.005	0.014	0.050	0.009	0.002	0.053	0.021
Eustigmatophyceae	0	0	0	0	0	0	0	0.002	0	0
Klebsormidiophyceae	0	0	0	0	0	0.023	0	0	0.002	0
Prymnesiophyceae	0	0.002	0.000	0.002	0.003	0.004	0.002	0.003	0.008	0.007
Trebouxiophyceae	0	0	0.007	0	0	0.005	0.005	0	0	0.001
Xanthophyceae	0	0	0.008	0	0.005	0	0.003	0.001	0	0
Total	2.306	2.344	3.684	1.474	1.264	0.827	1.493	2.467	1.238	2.790

**Table 2:** Strains isolated from Meiktila Lake, strain codes and European Nucleotide Archive (ENA) accession numbers.

Species	Strain	Accession nr. 16S rRNA Gene
*Raphidiopsis*		
*R. raciborskii*	AB2017/05	LR590626
*R. raciborskii*	AB2017/09	LR590627
*R. raciborskii*	AB2017/12	LR590628
*R. raciborskii*	AB2017/13	LR590629
*R. raciborskii*	AB2017/16	LR746263
*Microcystis*		
*Microcystis*	AB2017/14	LR590630
*Microcystis*	AB2017/15	LR590631

**Table 3:** Concentrations (µg mg^-1^ FW) of CYNs by ELISA and of CYN and deoxyCYN by LC–MS/MS in cultured *R. raciborskii* strains isolated from Meiktila Lake*.

Strain	ELISA	LC-MS/MS			
	CYNs	CYN	deoxyCYN	CYN (%)	deoxyCYN (%)
AB2017/09	2.18	-	-	38	62
AB2017/05	n.d.	n.d.	n.d.	n.d.	n.d.
AB2017/16	1.84	1.65	1.25	57	43
AB2017/12	n.d.	n.d.	n.d.	n.d.	n.d.
AB2017/13	4.31	2.46	7.29	25	75

*- = biomass not determined; n.d. = not detected; FW = fresh weight; percentages are of total CYNs by LC–MS/MS.

**Table 4:** Identities of microcystins detected by LC-HRMS/MS analysis in *Microcystis* strains AB2017/14 and /15 isolated from Meiktila Lake, their retention times (*t*_R_), concentrations, relative abundances (%) and observed *m*/*z* values in negative ionisation mode*^a^.*

	*m*/*z*	Compound Name	Confidence	*t*_R_ (min)	Concentration*^b^*			
					AB2017/14		AB2017/15	
					μg g^−1^	%	μg g^−1^	%
**1**	1022.5443	[D-Asp^3^]MC-RR*^c^*	Confirmed	4.18	2.4	0.21	1002.4	7.21
**2**	1022.5454	[Dha^7^]MC-RR*^c^*	Probable	4.53	0.1	0.01	31.8	0.23
**3**	1036.5597	MC-RR*^c^*	Confirmed	4.55	19.4	1.74	1458.0	10.49
**4**	995.4858	[D-Asp^3^]MC-ER*^c^*	Probable	6.14	ND	-	119.6	0.86
**5**	1114.5657	MC-LR–Cys*^d^*	Probable	6.16	0.1	0.01	ND	-
**6**	1045.5378	MC-(H2)YR*^c^*	Probable	6.18	32.4	2.90	ND	-
**7**	1031.5224	[D-Asp^3^]MC-(H2)YR*^c^*	Probable	6.20	32.9	2.95	ND	-
**8**	1033.5381	[D-Asp^3^]MC-(H4)YR*^c^*	Probable	6.27	0.6	0.05	148.1	1.07
**9**	1009.5015	MC-ER*^c^*	Probable	6.55	0.6	0.05	37.8	0.27
**10**	1029.5072	[D-Asp^3^]MC-YR*^c^*	Probable	6.59	ND	-	144.1	1.04
**11**	1047.5540	MC-(H4)YR*^c^*	Probable	6.64	6.4	0.57	310.8	2.24
**12**	1011.5530	[Mser^7^]MC-LR	Probable	6.80	0.4	0.04	13.3	0.10
**13**	979.5273	[D-Asp^3^]MC-LR*^c^*	Confirmed	6.89	40.8	3.65	1870.3	13.45
**14**	1043.5224	MC-YR*^c^*	Confirmed	6.99	9.1	0.81	317.4	2.28
**15**	1011.4999	MC-MR*^c^*^,*d*,*e*^	Probable	6.99	3.6	0.32	ND	-
**16**	1073.5329	MC-Y(OMe)R*^c^*	Probable	7.07	7.2	0.64	ND	-
**17**	993.5435	MC-LR*^c^*	Confirmed	7.12	183.0	16.38	3332.5	23.97
**18**	979.5289	[Dha^7^]MC-LR*^c^*	Confirmed	7.13	0.8	0.07	37.4	0.27
**19**	1080.5170	Unidentified MC*^c^*	Unidentified	7.15	59.0	5.28	ND	-
**20**	1070.5332	MC-KynR*^c^*	Probable	7.35	4.5	0.40	ND	-
**21**	1007.5582	MC-HilR*^c^*	Confirmed	7.38	41.8	3.74	17.9	0.13
**22**	1080.5169	Unidentified*^c^*	Unidentified	7.38	59.6	5.34	ND	-
**23**	1082.5311	MC-OiaR*^c^*	Tentative	7.40	102.4	9.17	ND	-
**24**	995.4857	[D-Asp^3^]MC-RE*^c^*	Probable	7.43	ND	-	110.1	0.79
**25**	1027.5269	MC-FR*^c^*	Confirmed	7.48	255.2	22.85	ND	-
**26**	1066.5380	MC-WR*^c^*	Confirmed	7.64	0.7	0.06	ND	-
**27**	1009.5015	MC-RE*^c^*	Probable	7.79	3.8	0.34	1281.0	9.21
**28**	951.4955	MC-RA*^c^*	Probable	8.24	13	1.16	ND	-
**29**	965.5116	MC-RAbu*^c^*	Probable	8.83	7.1	0.64	ND	-
**30**	946.4577	MC-(H2)YG*^c^*	Probable	9.06	5.4	0.48	ND	-
**31**	1011.4966	MC-RM*^c^*^,*d*,*e*^	Probable	9.23	0.2	0.02	ND	-
**32**	960.4738	MC-(H2)YA*^c^*	Probable	9.34	10.1	0.90	ND	-
**33**	953.4783	Unidentified MC*^c^*	Unidentified	9.50	12	1.07	ND	-
**34**	967.4942	Unidentified MC*^c^*	Unidentified	9.78	2.3	0.21	ND	-
**35**	984.4735	Unidentified MC*^c^*	Unidentified	9.96	3.7	0.33	ND	-
**36**	968.4272	[D-Asp^3^]MC-EE*^c^*	Probable	11.43	ND	-	403.3	2.90
**37**	982.4432	MC-EE*^c^*	Probable	12.58	0.9	0.08	1533.5	11.03
**38**	952.4690	[D-Asp^3^]MC-LE*^c^*	Probable	12.82	1.2	0.11	358.8	2.58
**39**	894.4631	[D-Asp^3^]MC-LA*^c^*	Probable	13.32	6.5	0.58	ND	-
**40**	966.4849	MC-LE*^c^*	Probable	13.69	12.9	1.15	1376.1	9.90
**41**	908.4788	MC-LA*^c^*	Confirmed	14.87	44.8	4.01	ND	-
**42**	922.4942	MC-HilA*^c^*	Probable	15.29	5.9	0.53	ND	-
**43**	981.4737	MC-WA*^c^*	Probable	15.88	4.4	0.39	ND	-
**44**	942.4629	MC-FA*^c^*	Probable	15.95	42.3	3.79	ND	-
**45**	922.4943	MC-LAbu*^c^*	Probable	16.09	16.6	1.49	ND	-
**46**	936.5110	MC-HilAbu*^c^*	Probable	16.55	2.2	0.20	ND	-
**47**	995.4898	MC-WAbu*^c^*	Probable	17.20	13.4	1.20	ND	-
**48**	956.4784	MC-FAbu*^c^*	Probable	17.39	26.4	2.36	ND	-
**49**	936.5106	MC-LV*^c^*	Probable	17.62	3.6	0.32	ND	-
**50**	950.5254	MC-LL*^c^*	Probable	17.93	3.9	0.35	ND	-
**51**	970.4944	MC-FV*^c^*	Probable	18.40	0.7	0.06	ND	-
**52**	950.5262	*iso*-MC-LL*^c^*	Tentative	18.49	1.2	0.11	ND	-
**53**	1009.5041	MC-WV*^c^*	Probable	18.54	2.9	0.26	ND	-
**54**	1023.5210	MC-WL*^c^*	Probable	18.74	2.8	0.25	ND	-
**55**	970.4942	*iso*-MC-FV*^c^*	Tentative	18.79	2.1	0.19	ND	-
**56**	1023.5216	*iso*-MC-WL*^c^*	Tentative	19.16	1.3	0.12	ND	-

*^a^* A comprehensive version of this table, including positive and negative ionisation MS data, reactivity towards thiols and mild oxidising agents, number of rings plus double-bond equivalents (RDBE) and presence of characteristic ions observed in positive and negative ionisation MS/MS spectra, is in the Supporting Information (Table S1) together with LC–HRMS/MS spectra (Figures S1–S59). *^b^* Concentration expressed per weight of biomass (FW) and as a percentage of total microcystins detected in each culture); ND = not detected; *^c^* Reacted with mercaptoethanol; *^d^* Oxidised by Oxone/DMSO; *^e^* HRMS/MS spectrum of oxidation product obtained.

**Table 5:** Sampling points and sampling depth in Meiktila Lake for chemical and biological measurements.

Sampling Point	Water Depth (m)	Depth of Integrated Sample (m)	Geographical Position
MK1	3.3	0–1	N 20° 52’ 59.196, E 95° 51’ 12.204
MK2	2	0–1	N 20° 53’ 21.48, E 95° 51’ 2.124
MK3	2.5	0–1	N 20° 54’ 16.38, E 95° 50’ 40.092
MK4	4.4	0–2	N 20° 52’ 21.468, E 95° 51’ 12.528
MK5	7.1	0–3	N 20° 51’ 58.752, E 95° 51’ 18.936

## Supplementary Materials

The following are available online at https://www.mdpi.com/2072-6651/12/4/232/s1, Figures S1–S59: LC-HRMS/MS spectra of **1**, **3**, **4**, **6**, **7**, **9**, **11**–**15**, **17**, **19**–**25**, and **27**–**56**, Figure S60: LC-HRMS spectra of unidentified microcystins **33**–**35,** Table S1: Identities of microcystins detected by LC-HRMS/MS analysis in *M. aeruginosa* strains AB2017/14 and /15, their retention times (*t*_R_), concentrations, elemental compositions, observed *m*/*z* values in positive and negative ionisation modes, whether they matched retention times in reference samples and whether characteristic microcystin product ions were observed.
