# Supplementary material for: Cylindrospermopsin- and Deoxycylindrospermopsin-Producing Raphidiopsis raciborskii and Microcystin-Producing Microcystis spp. in Meiktila Lake, Myanmar
Source: Toxins (Basel). 2020 Apr 7;12(4):232. doi: 10.3390/toxins12040232 (PMC7232193; doi:10.3390/toxins12040232)
Supplement: Supplementary file 1 [file toxins-12-00232-s001.zip › Figures S1-S60 supp for ms update 2.pdf]

Supplementary Materials: Cylindrospermopsin- and Deoxycylindrospermopsin-Producing *Raphidiopsis raciborskii* and Microcystin-Producing *Microcystis* spp. in Meiktila Lake, Myanmar

Andreas Ballot, Thida Swe, Marit Mjelde, Leonardo Cerasino, Vladyslava Hostyeva and Christopher O. Miles

Table of Contents		Page
Figure S1	LC-HRMS/MS spectra of [D-Asp^3]MC-RR (**1**) and MC-RR (**3**)	S3
Figure S2	LC-HRMS/MS spectra of [D-Asp^3]MC-ER (**4**), MC-ER (**9**), [D-Asp^3]MC-RE (**24**), and MC-RE (**27**)	S4
Figure S3	LC-HRMS/MS spectra of MC-(H2)YR (**6**), MC-LR (**17**), and MC-(H4)YR (**11**)	S5
Figure S4	LC-HRMS/MS spectra of MC-HilR (**21**), MC-LR (**17**), and [D-Asp^3]MC-(H2)YR (**7**)	S6
Figure S5–7	LC-HRMS/MS spectra of MC-ER (**9**) and MC-LR (**17**)	S7–9
Figure S8	LC-HRMS/MS spectra of [Mser^7]MC-LR (**12**), MC-MR (**15**), and MC-RM (**31**)	S10
Figure S9	LC-HRMS/MS spectra of [D-Asp^3]MC-LR (**13**) and MC-LR (**17**)	S11
Figure S10	LC-HRMS/MS spectra of MC-LR (**17**) and MC-YR (**14**)	S12
Figure S11	LC-HRMS/MS spectra of MC-MR (**15**) and MC-M(O)R	S13
Figure S12	LC-HRMS/MS spectra of unidentified MCs **19** and **22**, and MC-OiaR (**23**)	S14
Figure S13	LC-HRMS/MS spectra of MC-LR (**17**) and MC-KynR (**20**)	S15
Figure S14	LC-HRMS/MS spectra of MC-FR (**25**), MC-M(O)R and MC-RM(O)	S16
Figure S15	LC-HRMS/MS spectra of MC-RM (**31**), MC-RA (**28**), and MC-RE (**27**)	S17
Figure S16	LC-HRMS/MS spectra of MC-RA (**28**) and MC-RAbu (**29**)	S18
Figure S17	LC-HRMS/MS spectra of MC-(H2)YG (**30**), MC-LA (**41**), and MC-(H2)YG (**32**)	S19
Figure S18	LC-HRMS/MS spectra of MC-LA (**41**) and unidentified MC **33**	S20
Figure S19	LC-HRMS/MS spectra of MC-LA (**41**) and unidentified MCs **33** and **34**	S21
Figure S20	LC-HRMS/MS spectra of MC-LA (**41**) and unidentified MC **35**	S22
Figure S21–26	LC-HRMS/MS spectra of [D-Asp^3]MC-LE (**38**), [D-Asp^3]MC-LA (**39**), and [D-Asp^3]MC-EE (**36**)	S19–28
Figure S27	LC-HRMS/MS spectra of MC-EE (**37**), [D-Asp^3]MC-EE (**36**), MC-LE (**40**), and [D-Asp^3]MC-LE (**38**)	S29
Figure S28–33	LC-HRMS/MS spectra of MC-LE (**40**), MC-LA (**41**), and MC-EE (**37**)	S30–35
Figure S34–36	LC-HRMS/MS spectra of MC-HilA (**42**), MC-LA (**41**), and MC-LAbu (**45**)	S36–38
Figure S37–39	LC-HRMS/MS spectra of MC-FA (**44**), MC-LA (**41**), and MC-WA (**43**)	S39–41
Figure S40–42	LC-HRMS/MS spectra of MC-HilA (**42**), MC-LA (**41**), and MC-HilAbu (**46**)	S42–44
Figure S43–45	LC-HRMS/MS spectra of MC-FAbu (**48**), MC-LAbu (**45**), and MC-WAbu (**47**)	S45–47
Figure S46–48	LC–HRMS/MS spectra of MC-LL (**50**), MC-LA (**41**), and MC-LV (**49**)	S48–50
Figure S49–51	LC–HRMS/MS spectra of MC-FV (**51**), MC-LV (**49**), and MC-WV (**53**)	S51–53
Figure S52–54	LC–HRMS/MS spectra of *iso*-MC-LL (**52**), MC-LL (**50**), and MC-WL (**54**)	S54–56
Figure S55	LC–HRMS/MS spectra of MC-FV (**51**), and *iso*-MC-FV (**55**)	S57
Figure S56	LC–HRMS/MS spectra of MC-WL (**54**), and *iso*-MC-WL (**56**)	S58
Figure S57–59	LC–HRMS/MS spectra of MC-WL (**54**), and standard of MC-LW	S59–61
Figure S60	LC–HRMS spectra of unidentified MCs **33**–**35**	S62
Table S1		S63

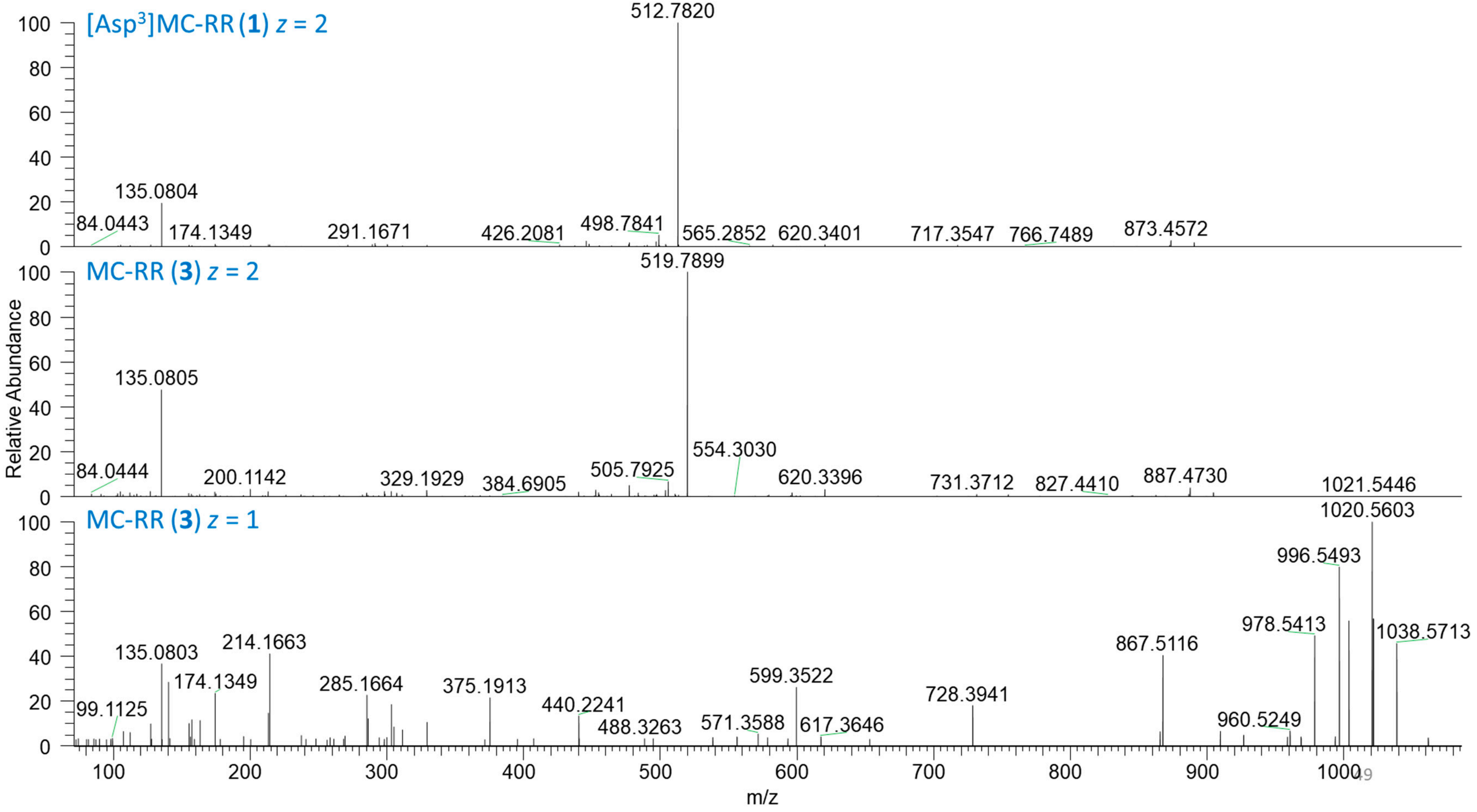

Figure S1. LC–HRMS/MS spectra of $[M+2H]^{2+}$ of [D-Asp³]MC-RR (**1**) and MC-RR (**3**), and of $[M+H]^{+}$ of **3**.

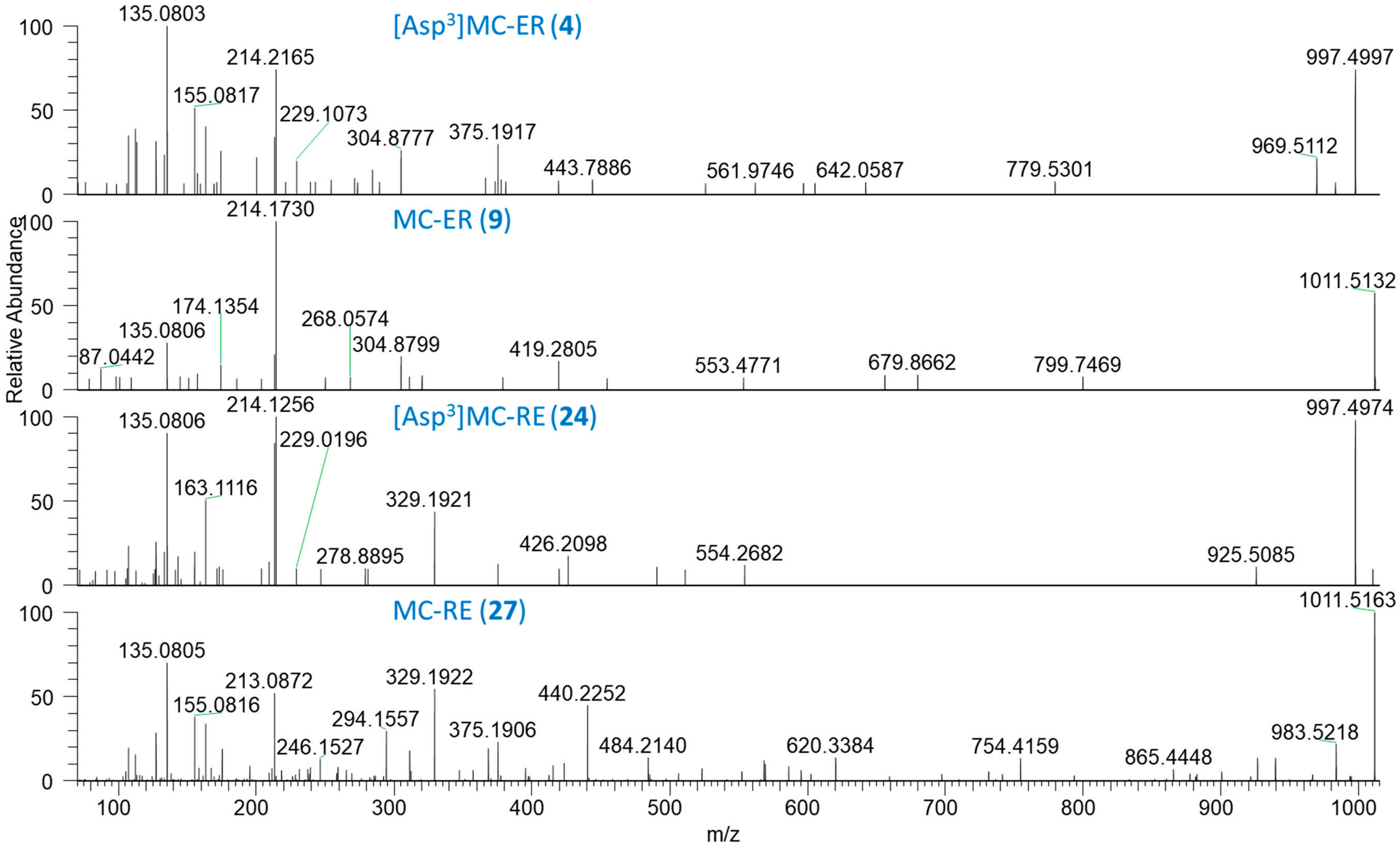

Figure S2. LC–HRMS/MS spectra of $[M+H]^+$ of [D-Asp³]MC-ER (**4**), MC-ER (**9**), [D-Asp³]MC-RE (**24**) and MC-RE (**27**).

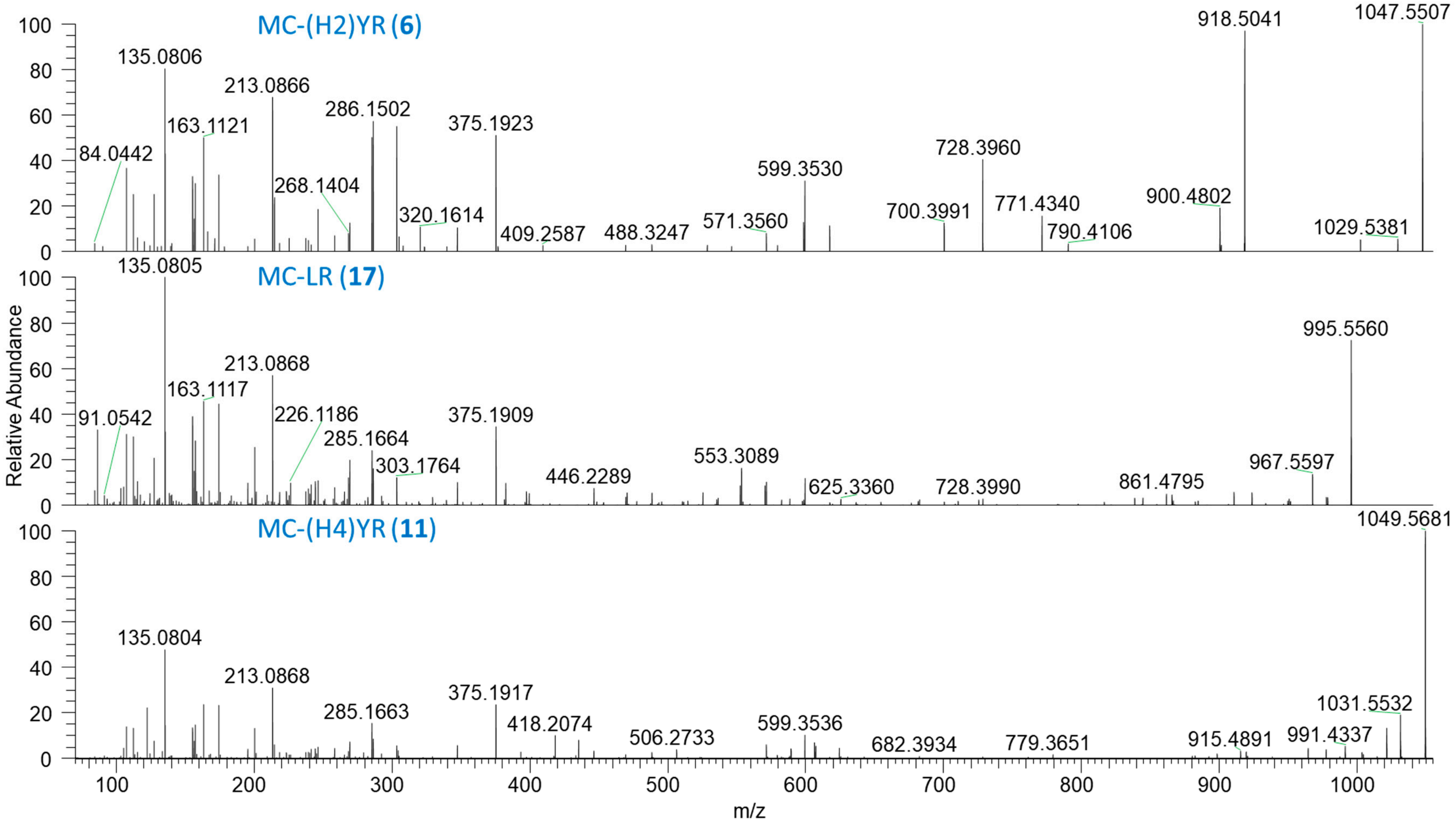

Figure S3. LC–HRMS/MS spectra of $[M+H]^+$ of MC-(H2)YR (**6**), MC-LR (**17**), and MC-(H4)YR (**11**).

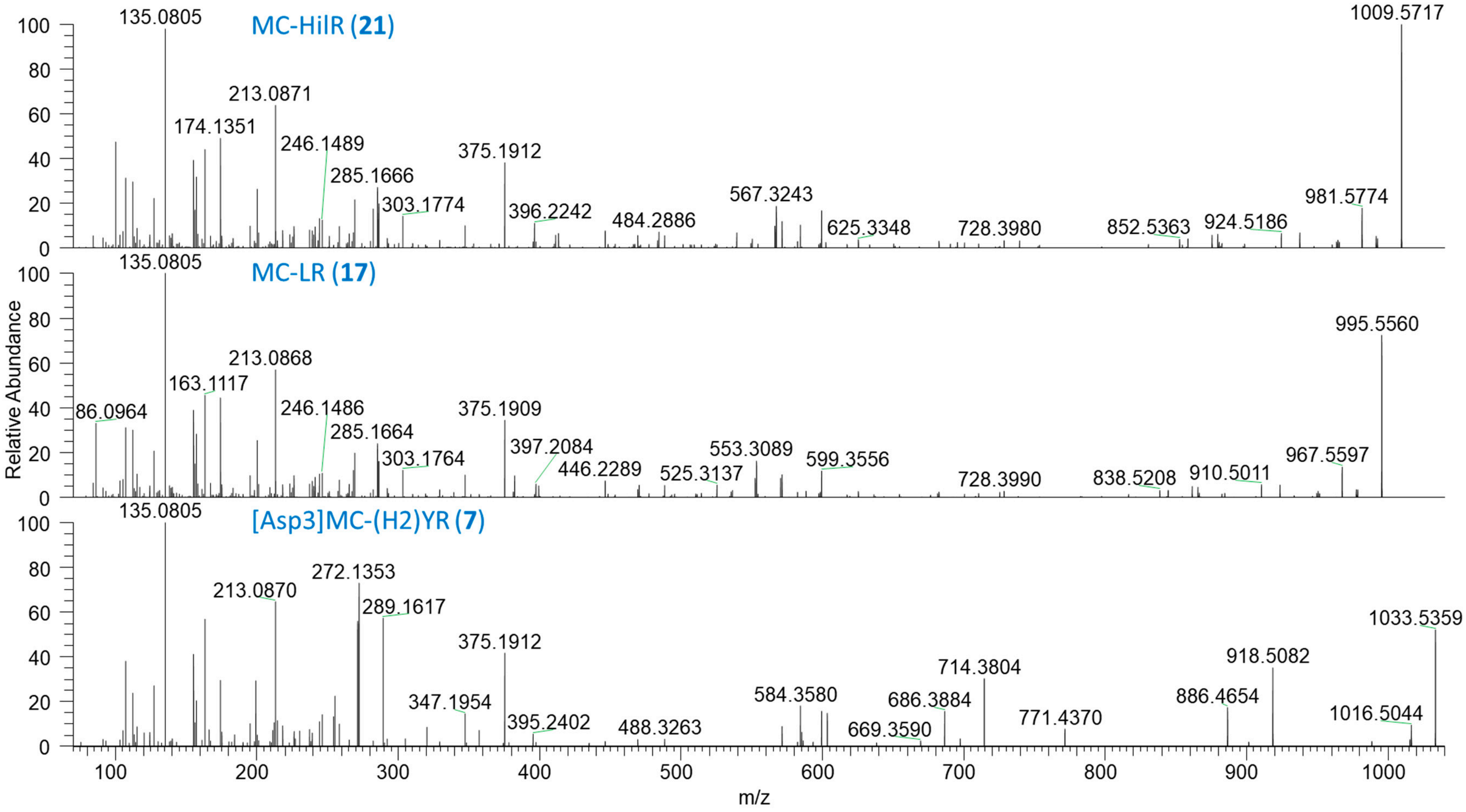

Figure S4. LC–HRMS/MS spectra of [M+H]$^+$ of MC-HilR (**21**), MC-LR (**17**), and [D-Asp^3]MC-(H2)YR (**7**).

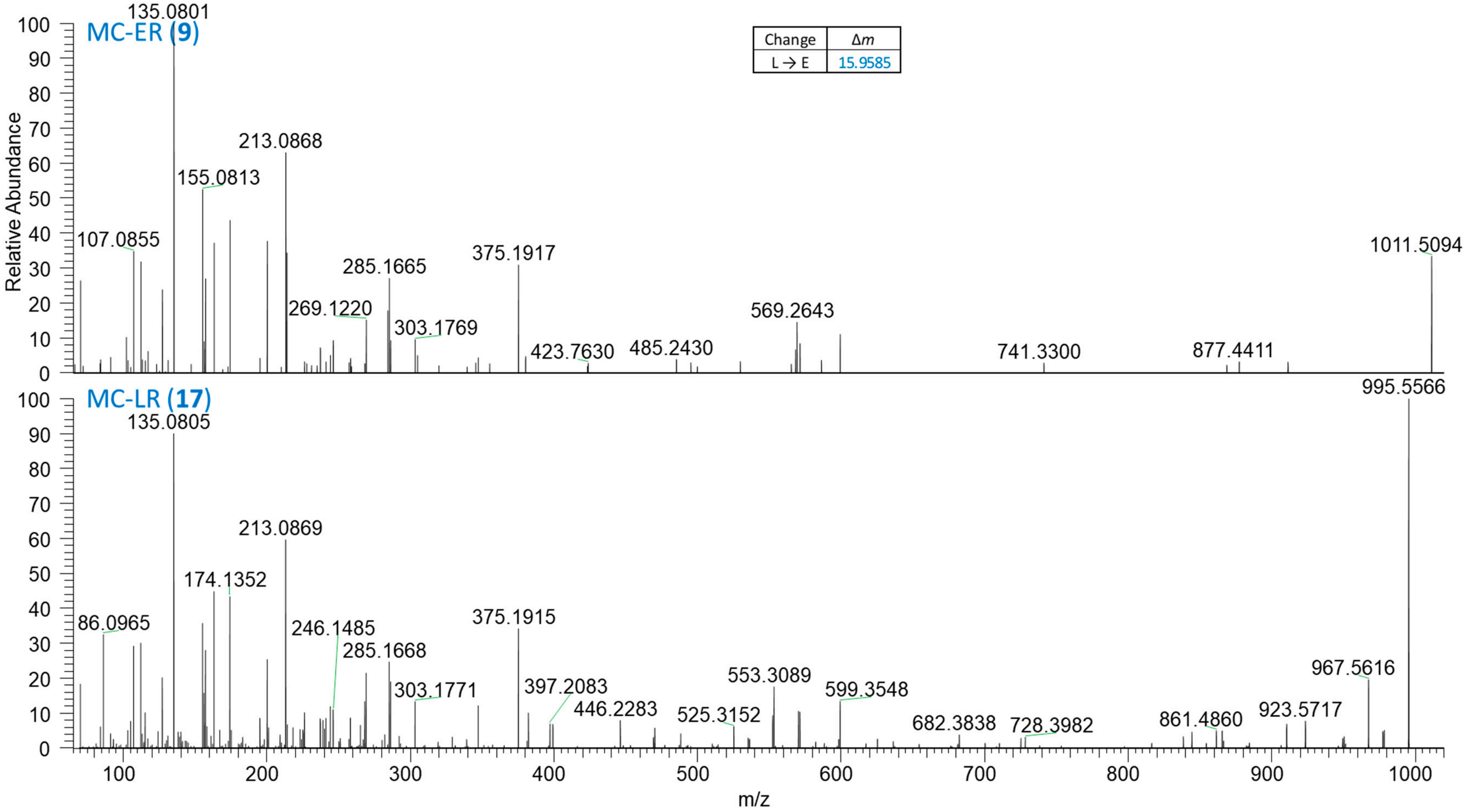

Figure S5. LC–HRMS/MS spectra of [M+H]+ of MC-ER (**9**) and MC-LR (**17**).

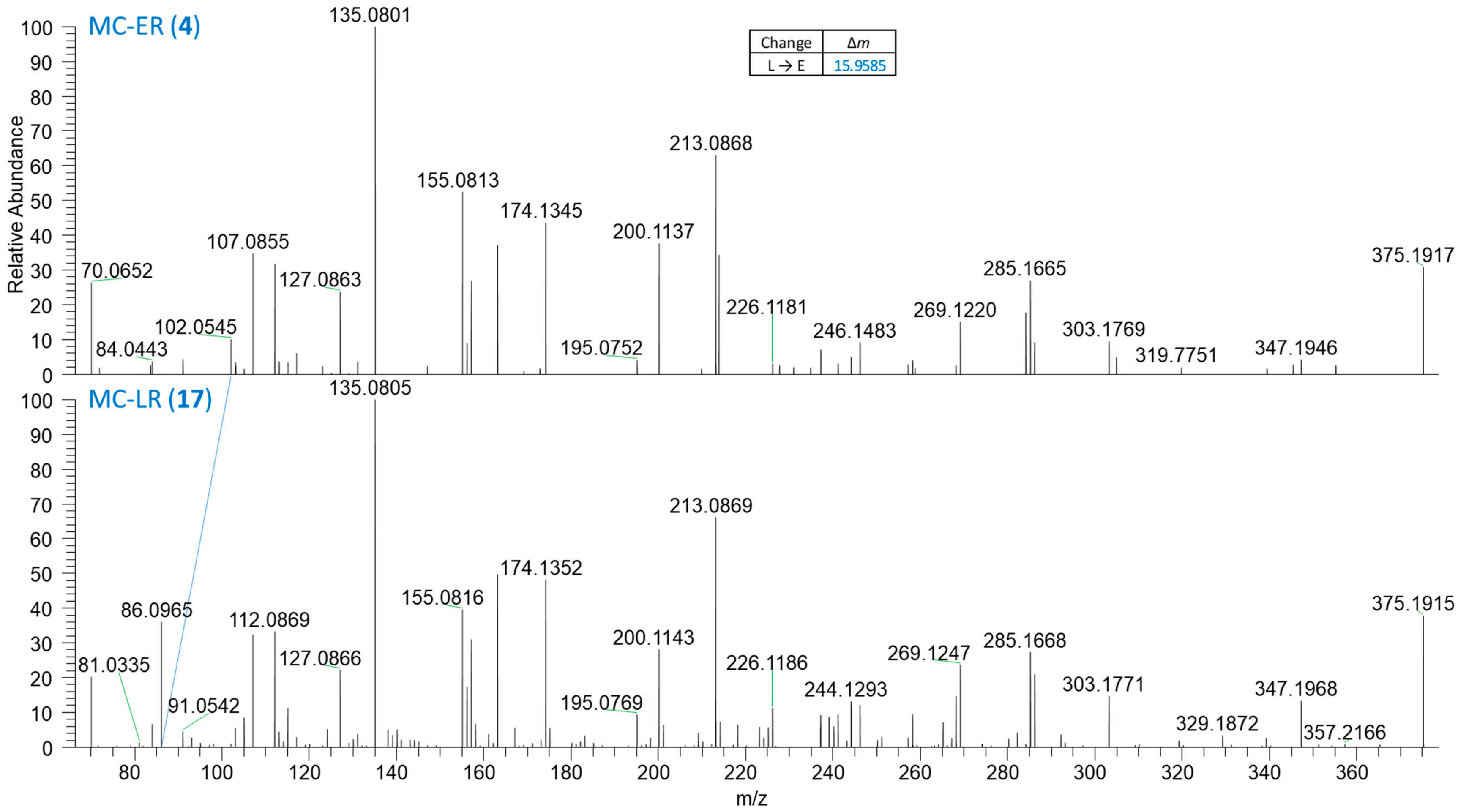

Figure S6. Expansion of LC–HRMS/MS spectra of [M+H]$^+$ of MC-ER (**9**) and MC-LR (**17**).

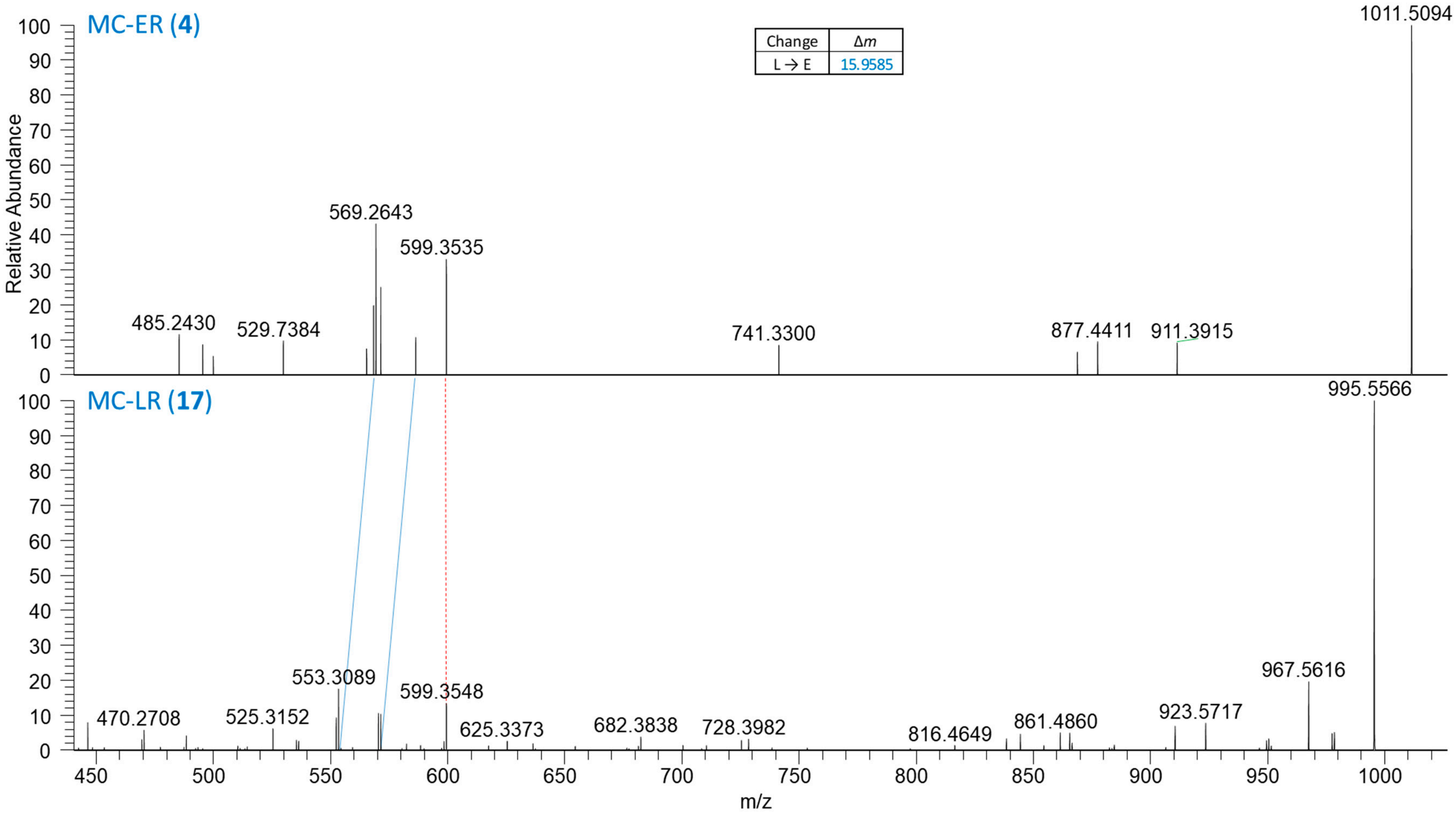

Figure S7. Expansion of LC–HRMS/MS spectra of $[M+H]^+$ of MC-ER (**9**) and MC-LR (**17**).

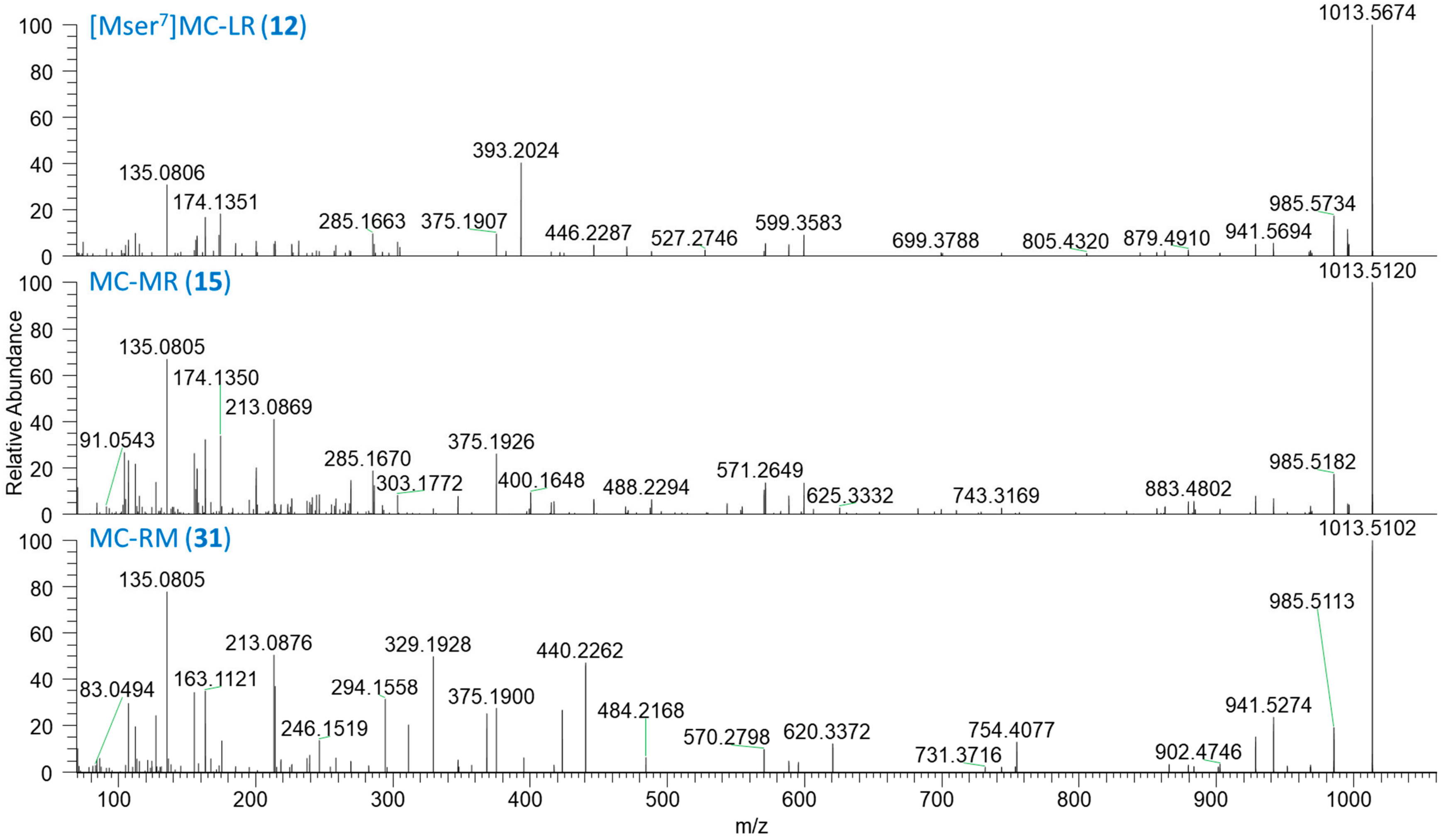

Figure S8. LC–HRMS/MS spectra of [M+H]+ of [Mser7]MC-LR (**12**), MC-MR (**15**), and MC-RM (**31**).

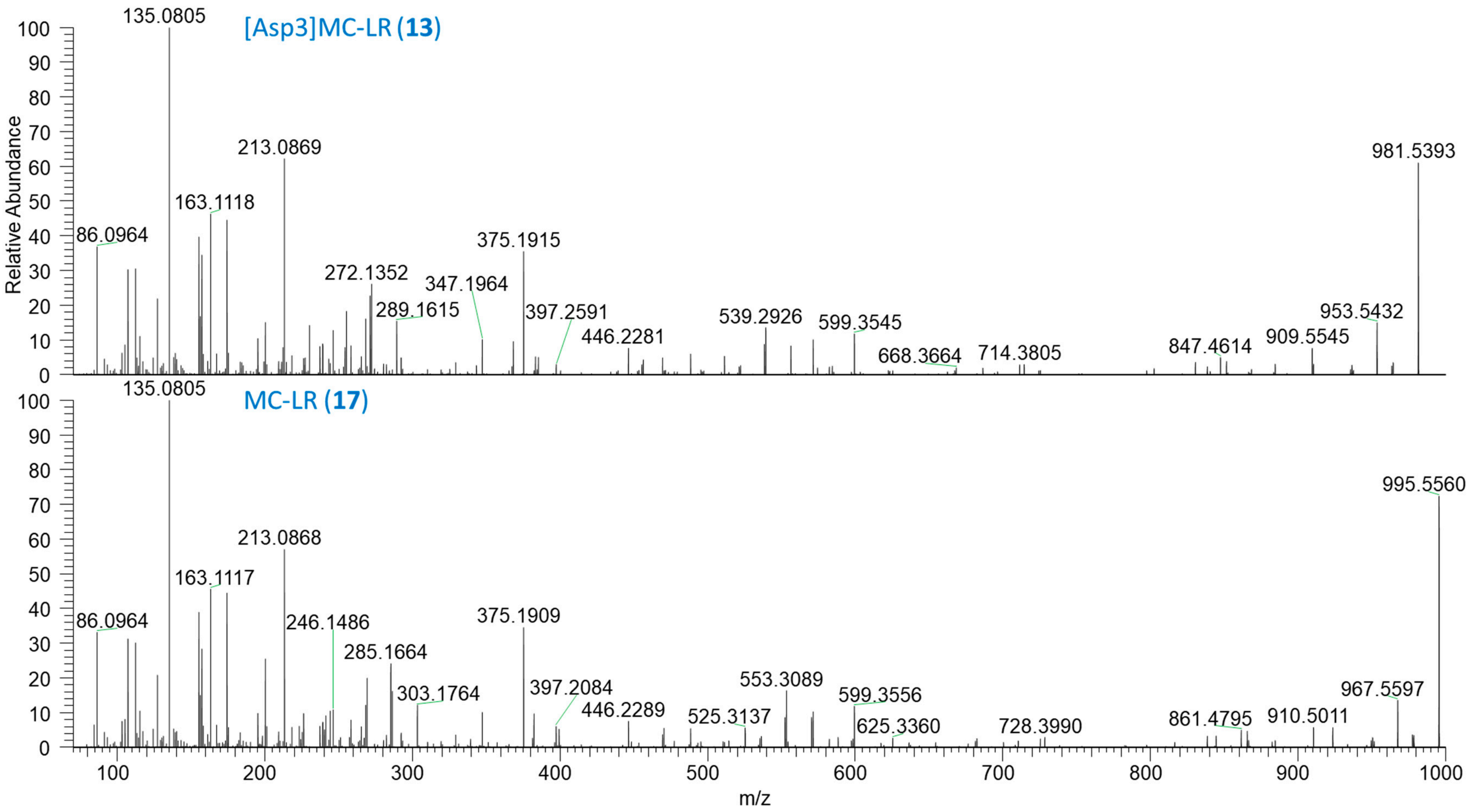

Figure S9. LC–HRMS/MS spectra of $[M+H]^+$ of [D-Asp^3]MC-LR (**13**), and MC-LR (**17**).

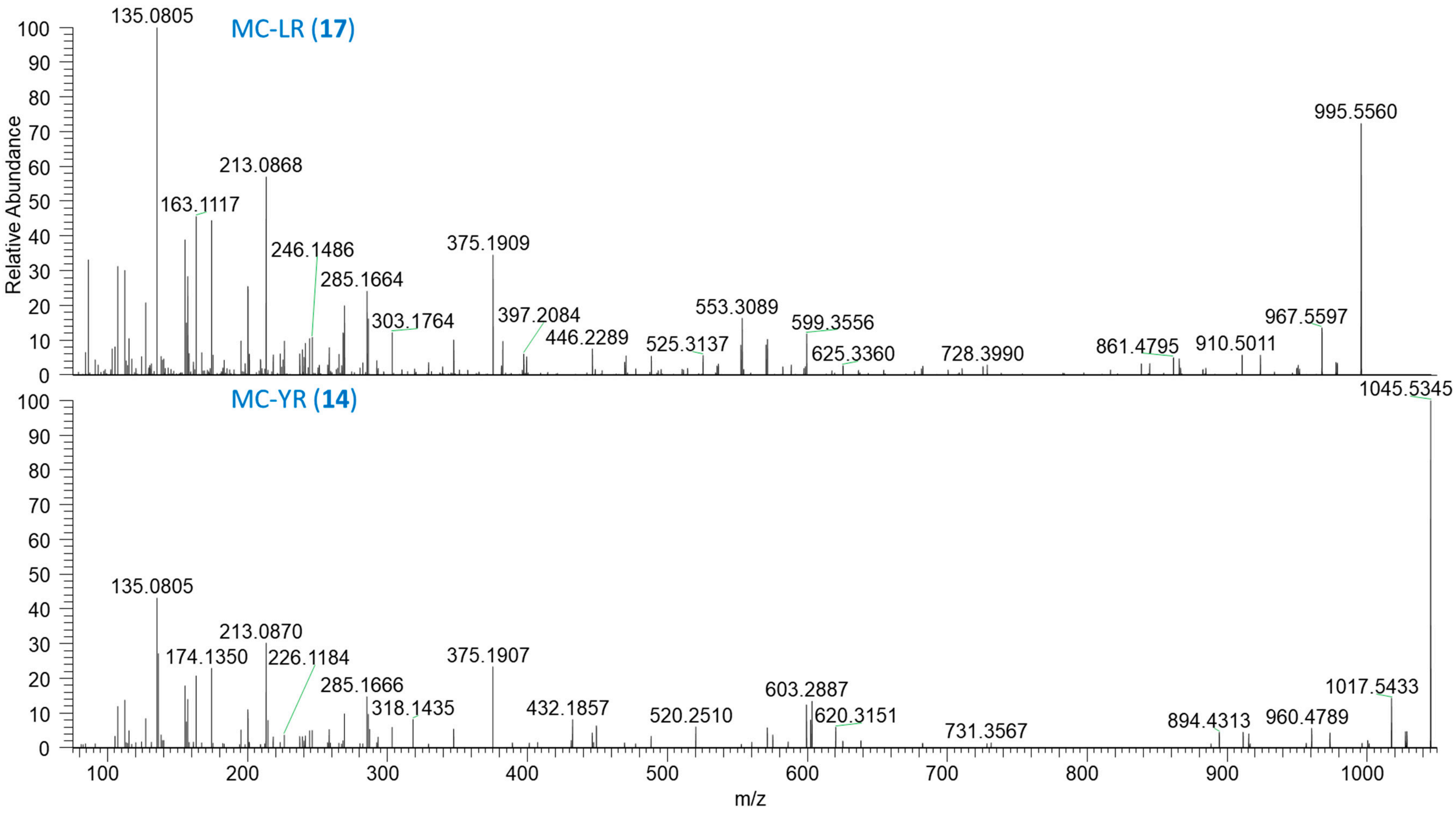

Figure S10. LC–HRMS/MS spectra of $[M+H]^+$ of MC-LR (**17**), and MC-YR (**14**).

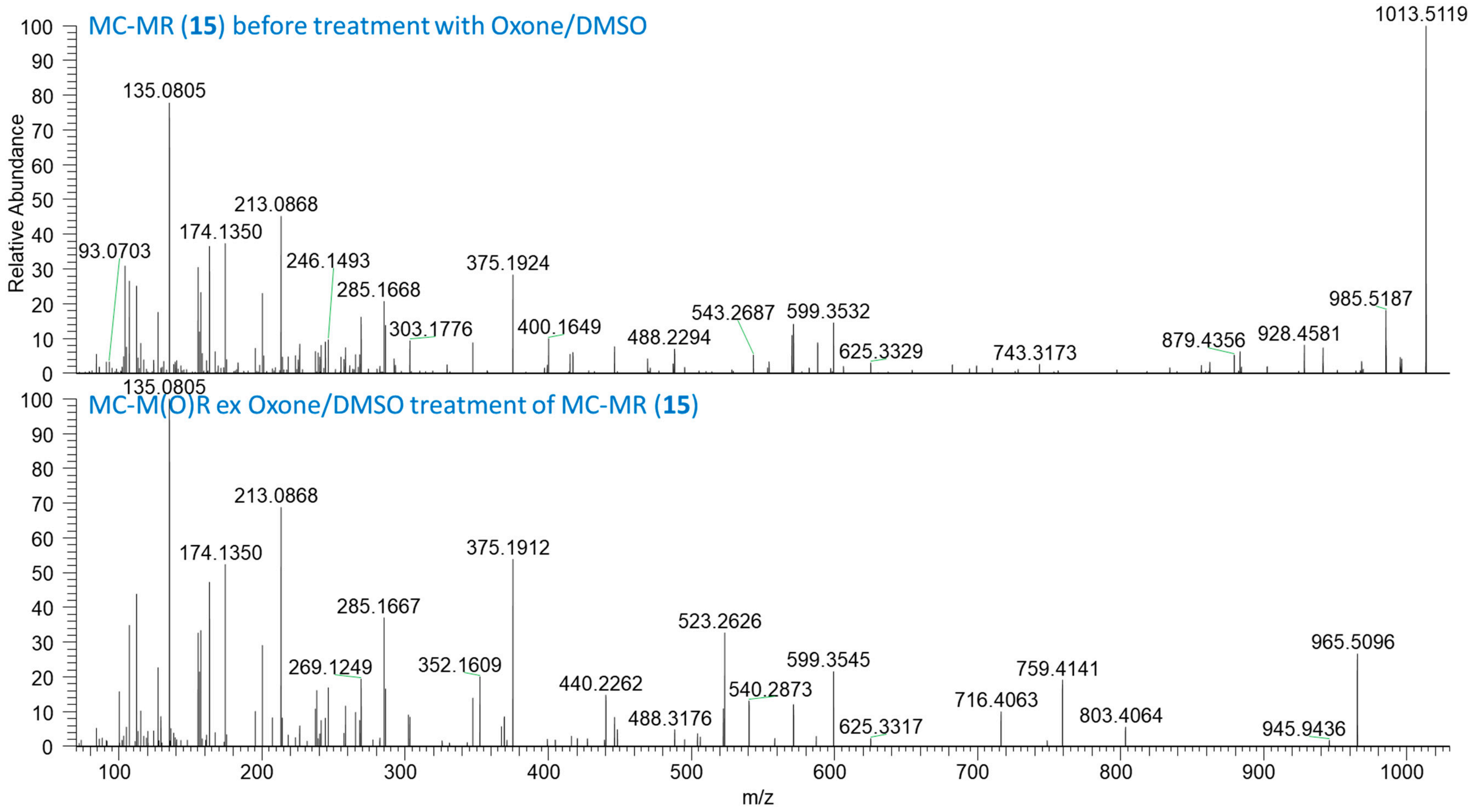

Figure S11. LC–HRMS/MS spectra of $[M+H]^+$ of MC-MR (**15**), and MC-M(O)R produced by oxidation of **15** in the sample with Oxone and DMSO.

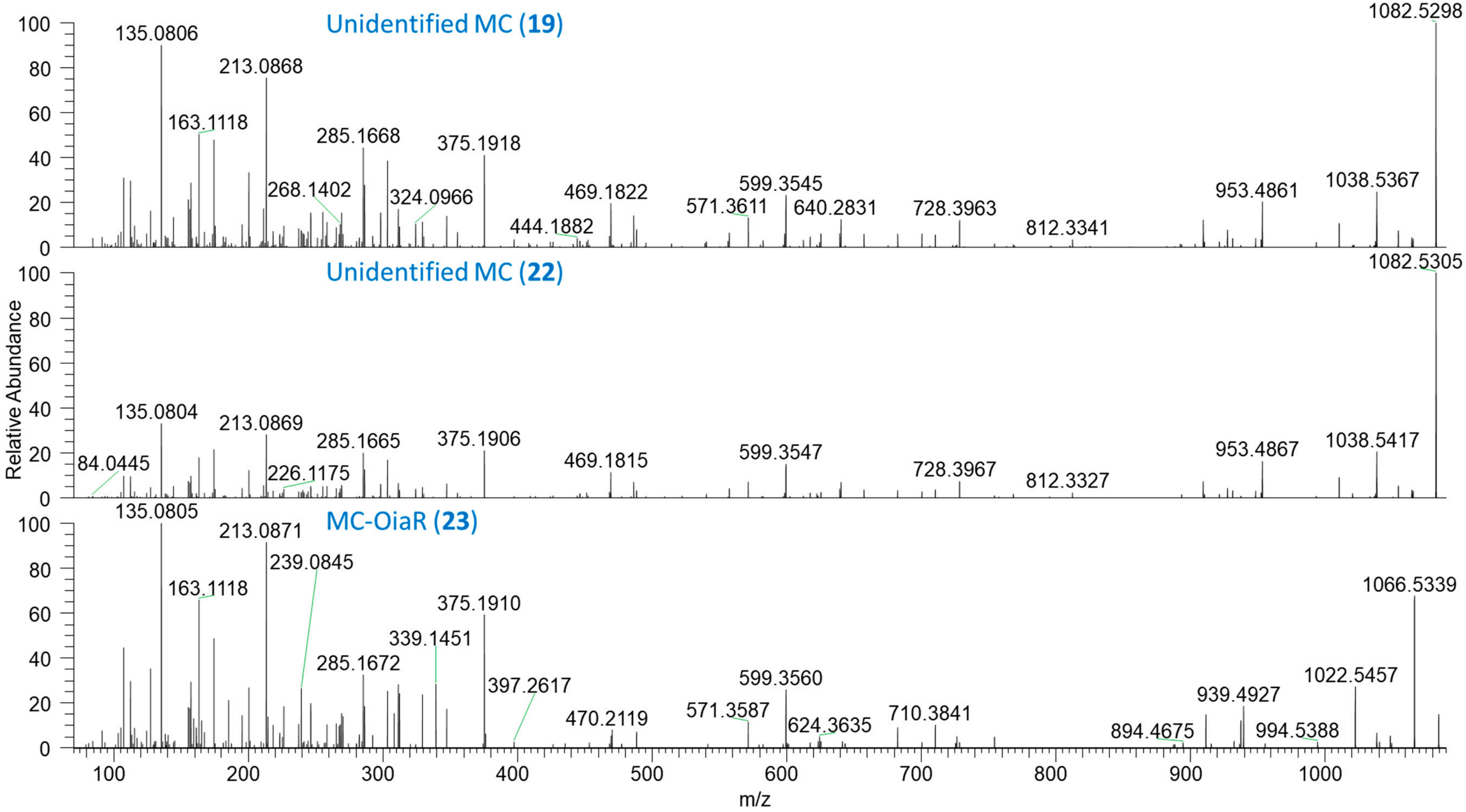

Figure S12. LC–HRMS/MS spectra of $[M+H]^+$ of unidentified microcystin-**19**, unidentified microcystin-**22**, and MC-OiaR (**23**).

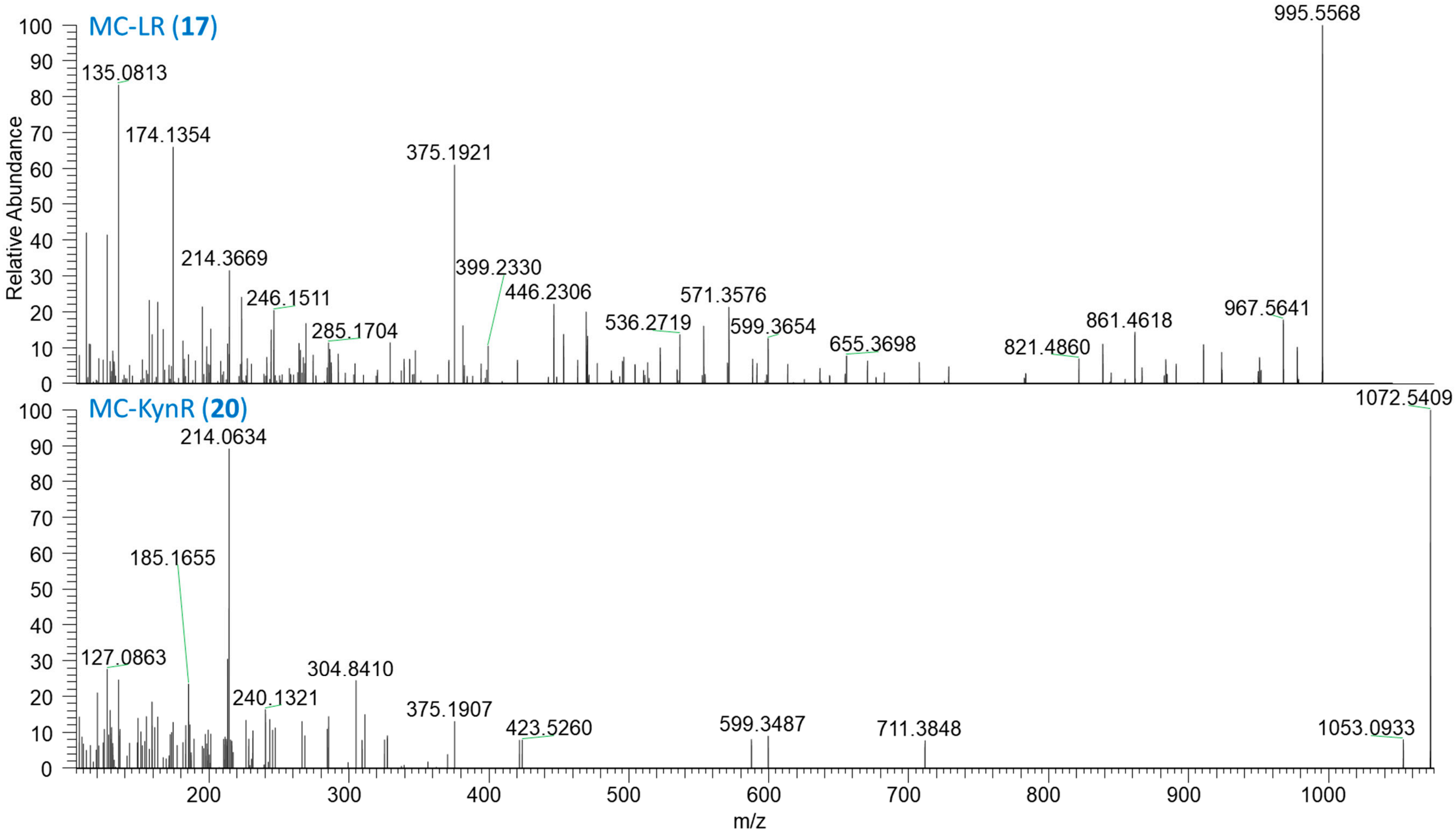

Figure S13. LC–HRMS/MS spectra of [M+H]$^+$ of MC-LR (**17**), and MC-KynR (**20**).

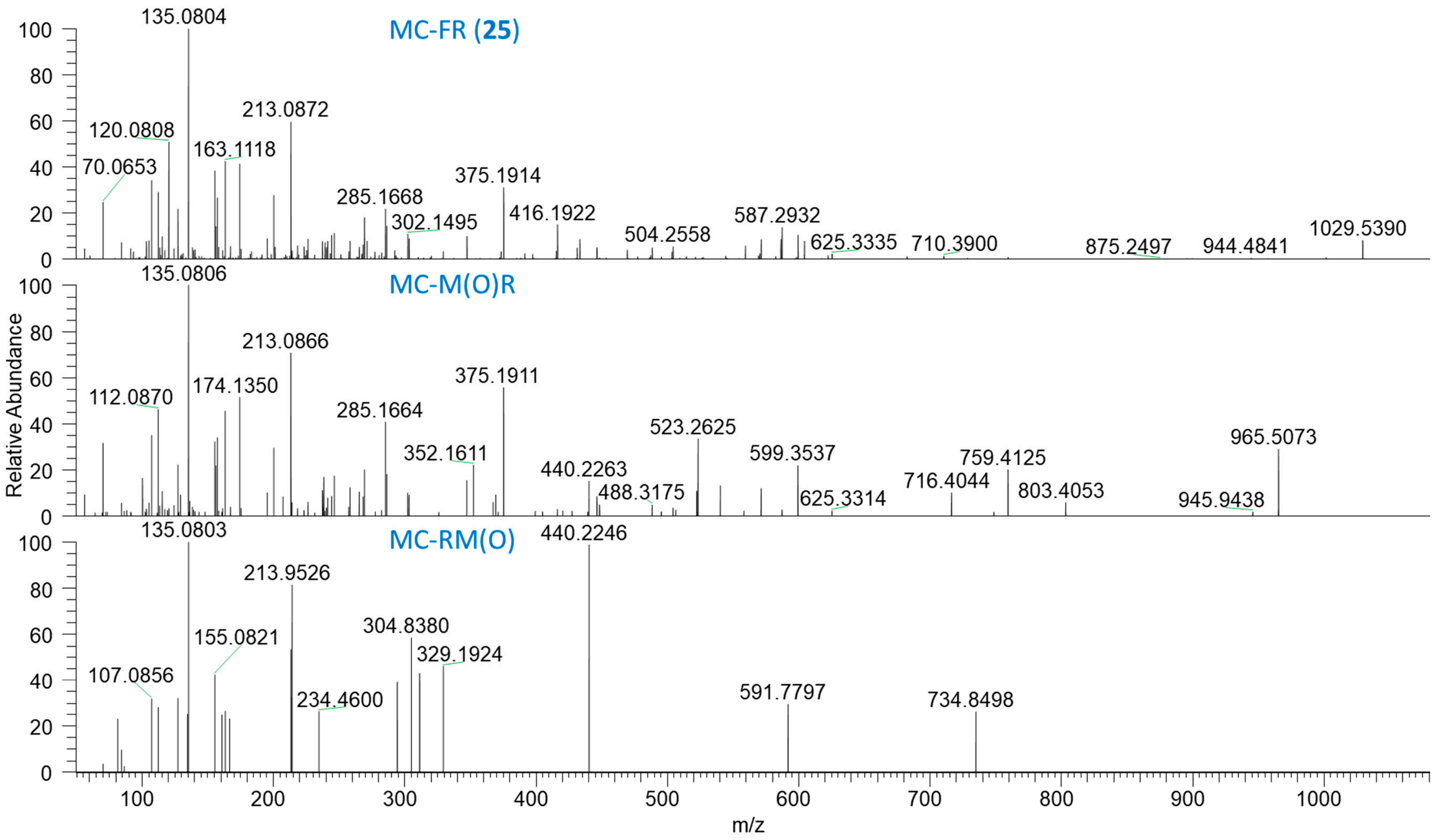

Figure S14. LC–HRMS/MS spectra of $[M+H]^+$ of MC-FR (**25**), and of MC-M(O)R and MC-RM(O) produced by oxidation of **15** and **31**, respectively, with Oxone/DMSO.

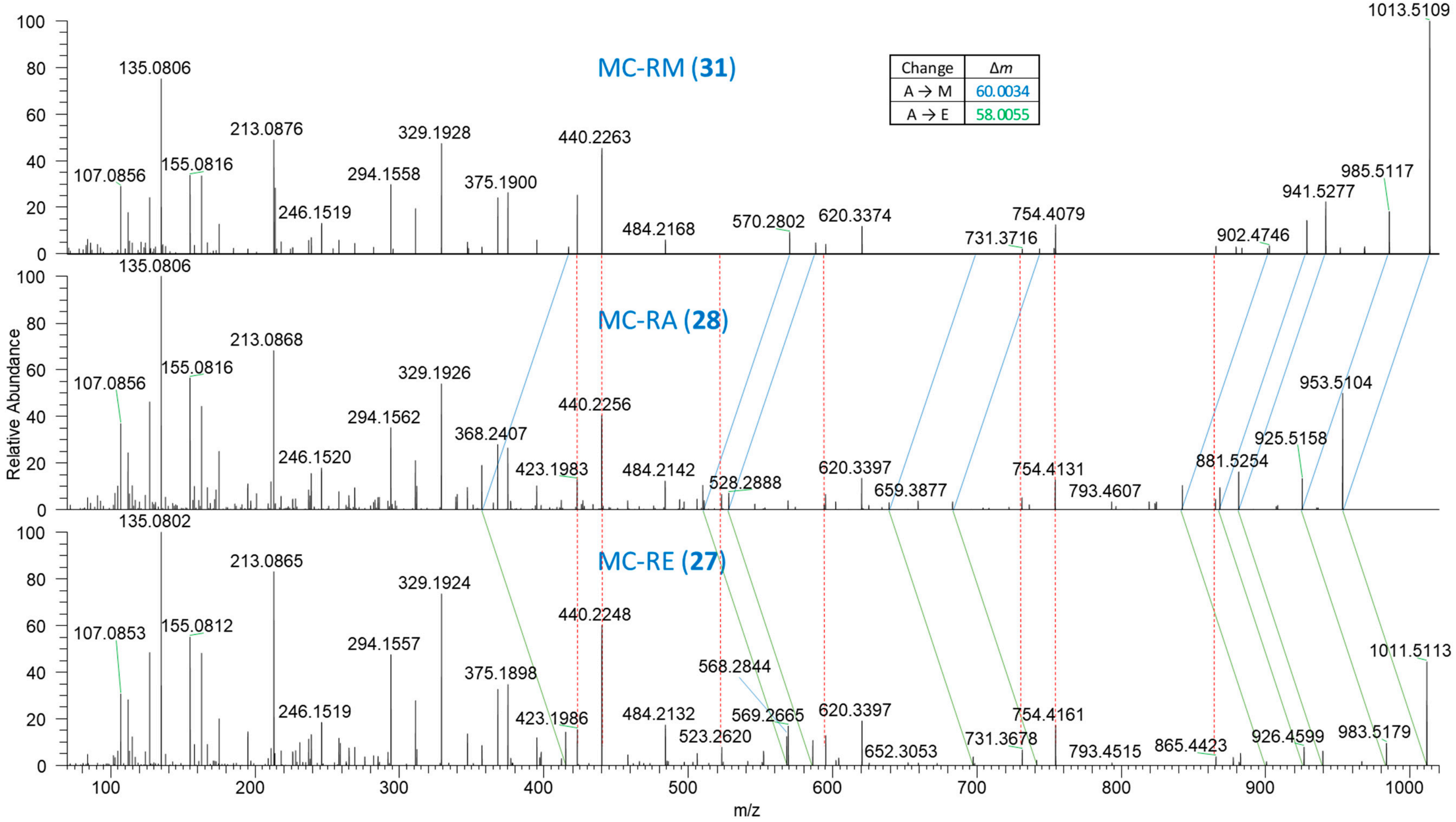

Figure S15. LC–HRMS/MS spectra of $[M+H]^+$ of MC-RM (**31**), MC-RA (**28**), and MC-RE (**27**).

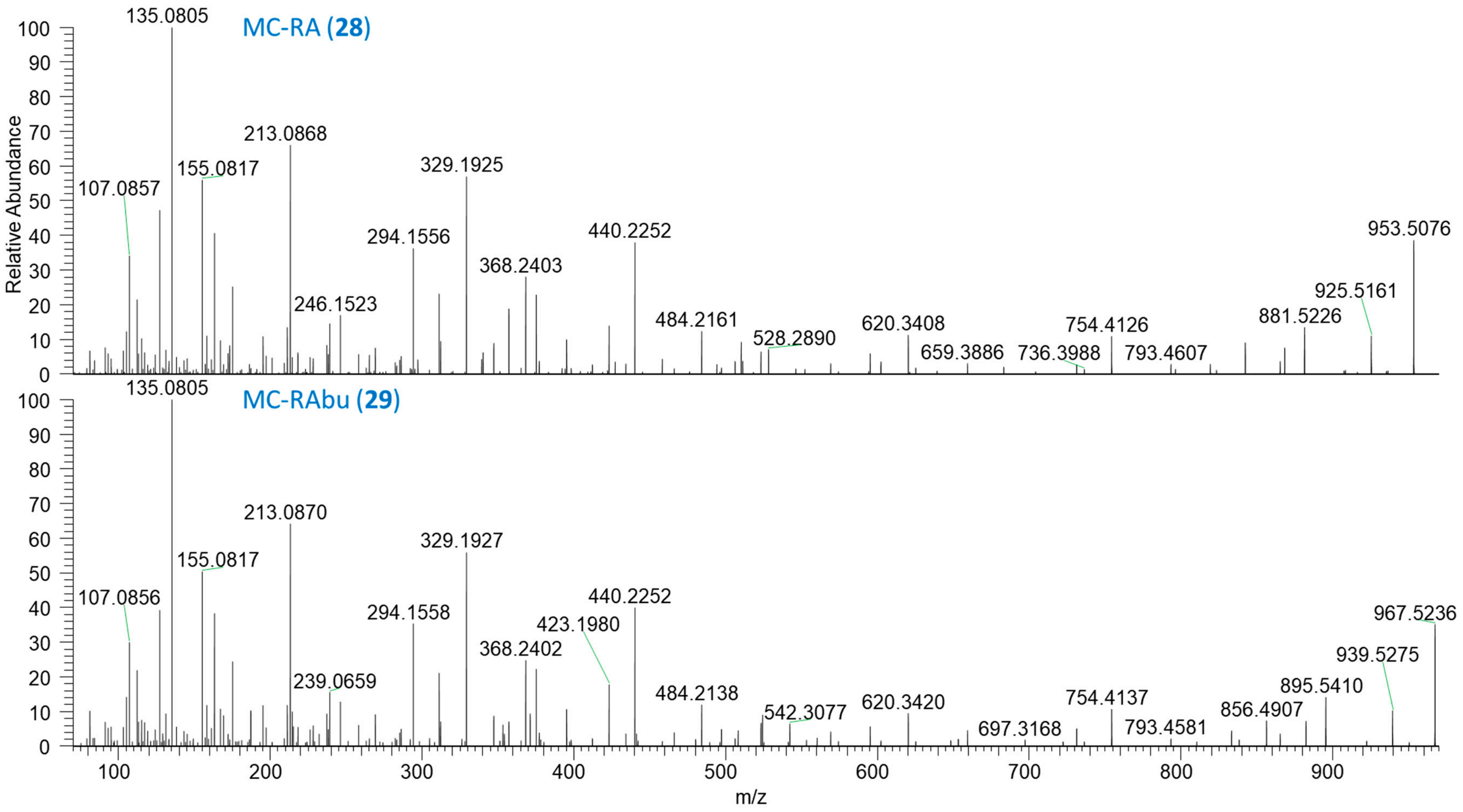

Figure S16. LC–HRMS/MS spectra of $[M+H]^+$ of MC-RA (**28**), and MC-RAbu (**29**).

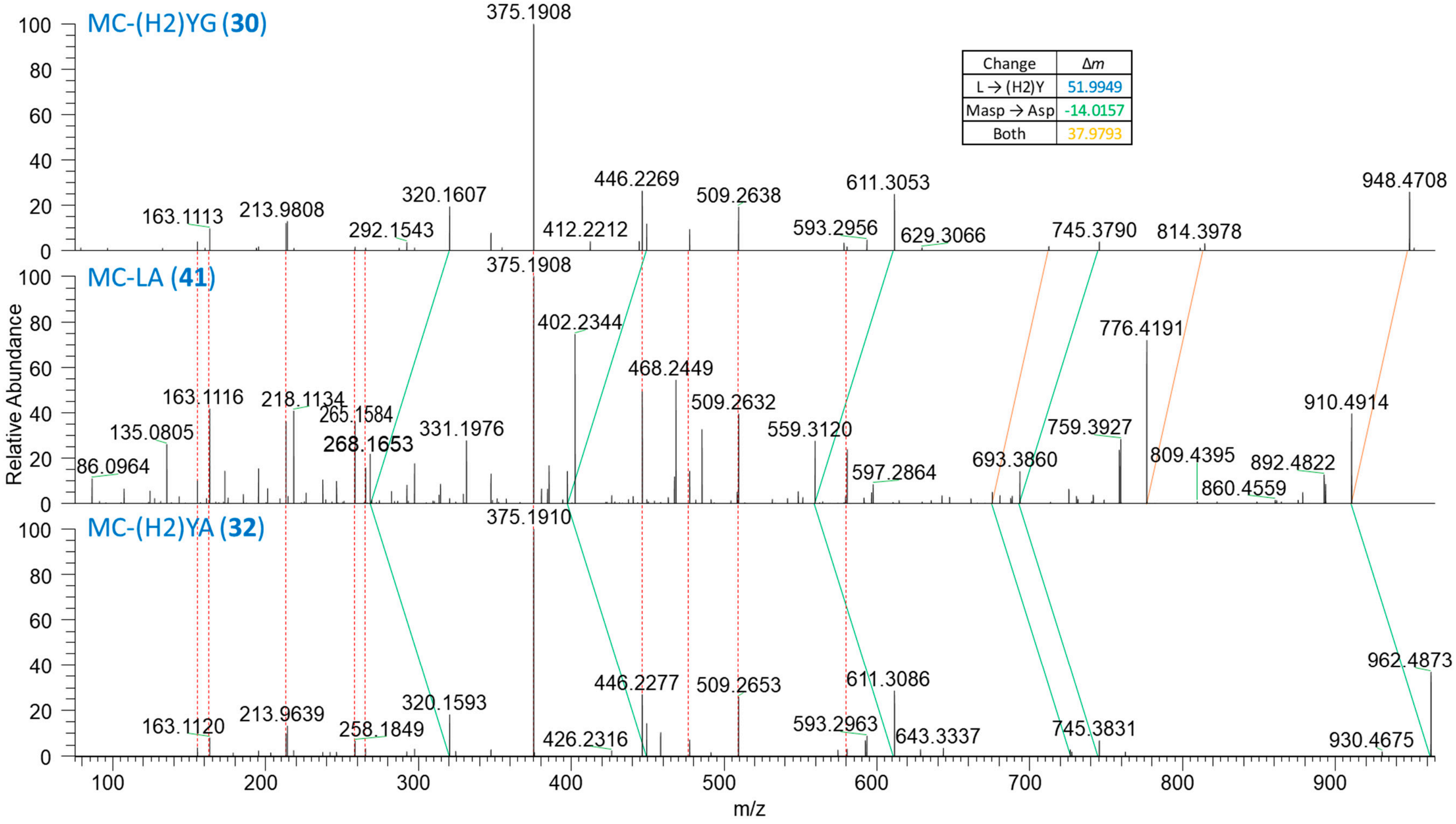

Figure S17. LC–HRMS/MS spectra of [M+H]$^+$ of MC-(H2)YG (**30**), MC-LA (**41**), and MC-(H2)YA (**32**).

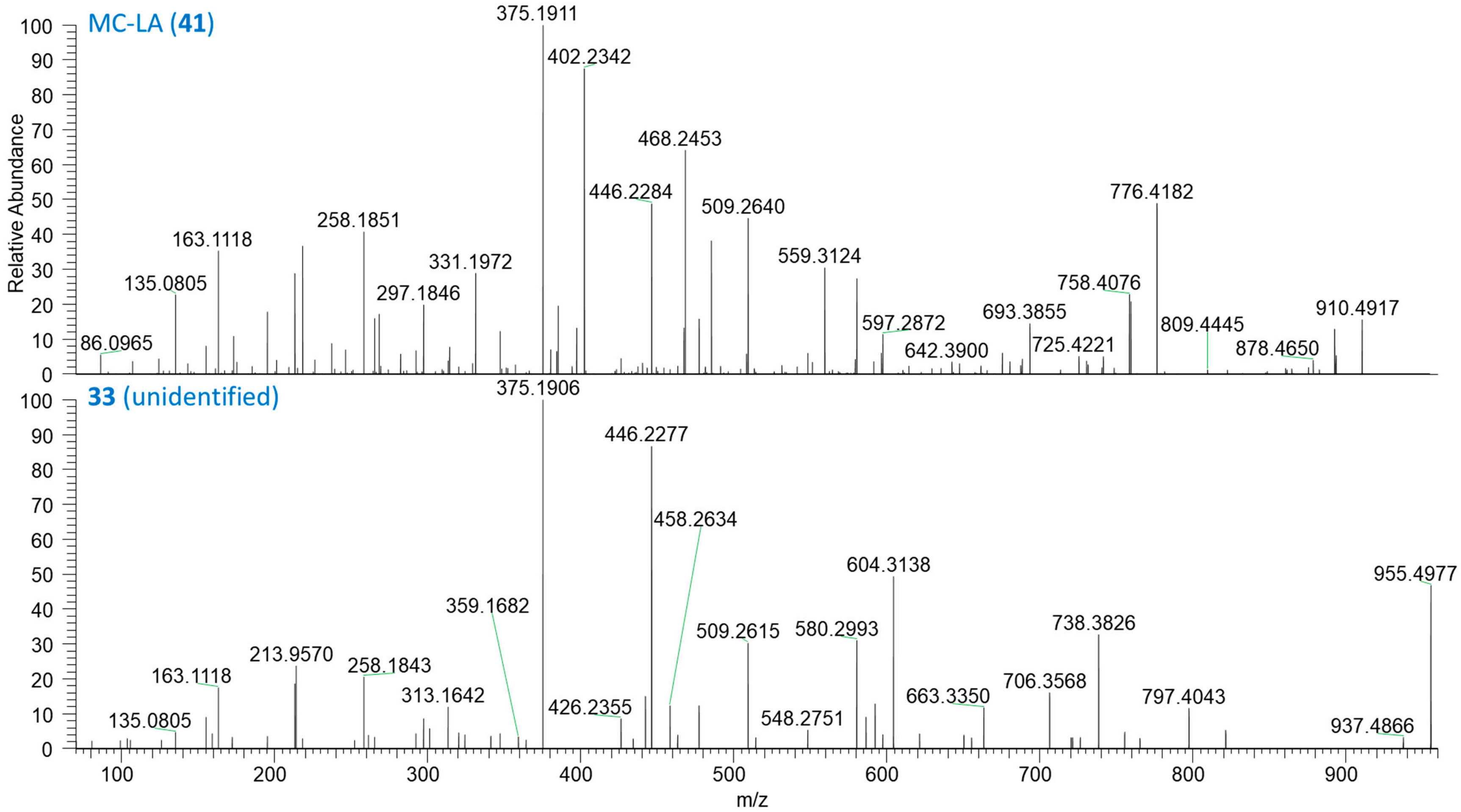

Figure S18. LC–HRMS/MS spectra of [M+H]+ of MC-LA (**41**), and unidentified microcystin-**33**.

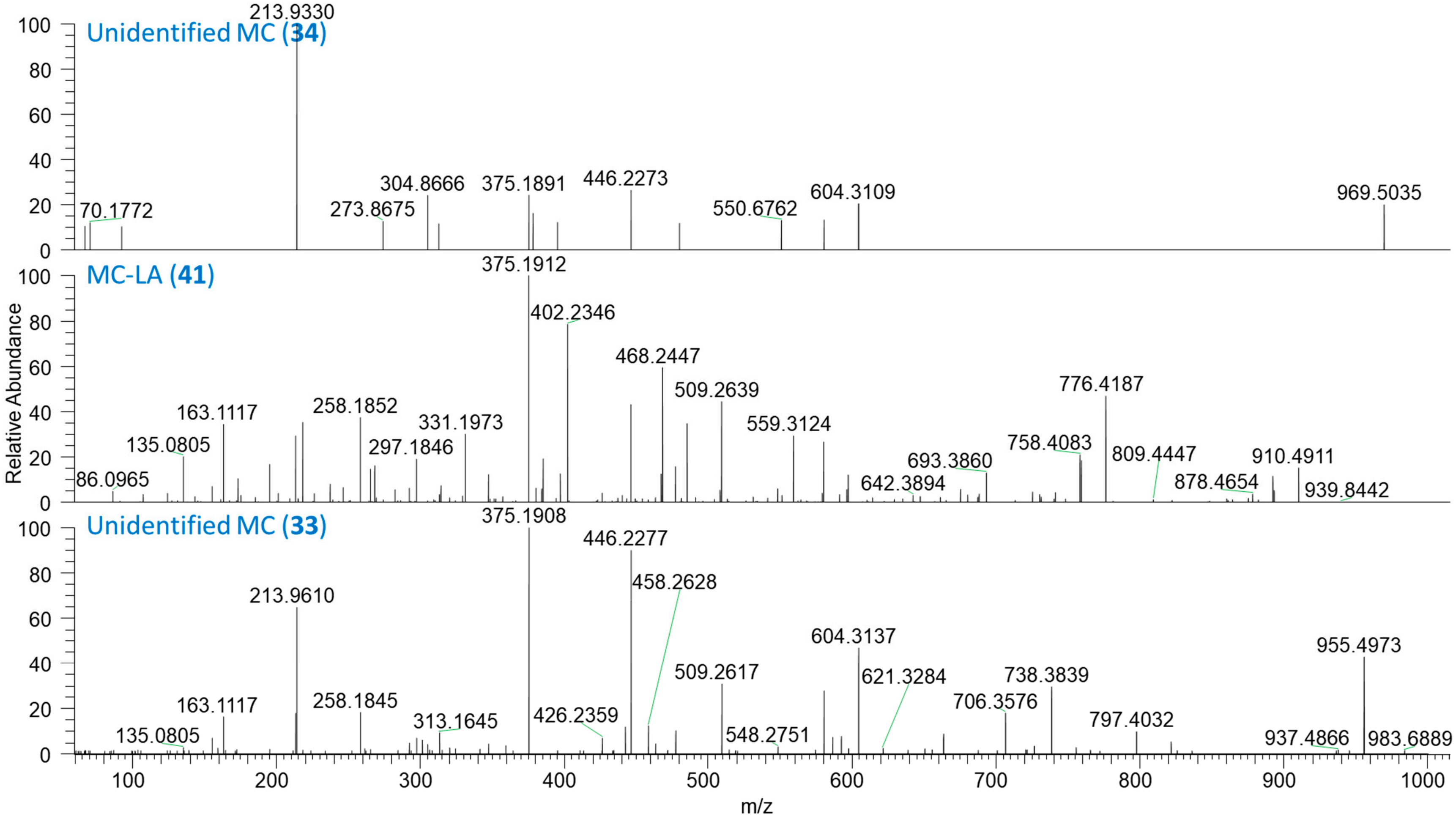

Figure S19. LC–HRMS/MS spectra of [M+H]⁺ of unidentified microcystin-**34**, MC-LA (**41**), and unidentified microcystin-**33**.

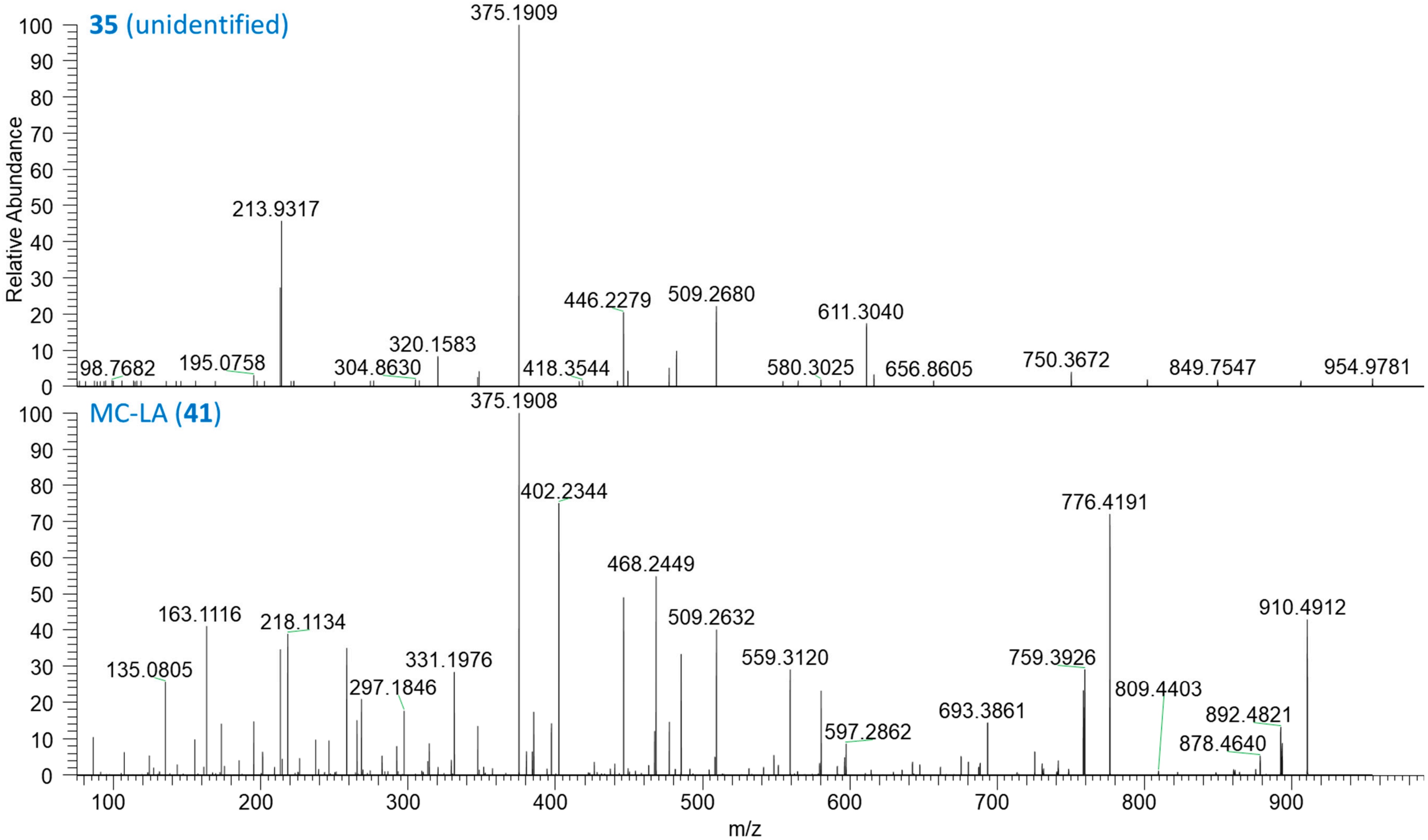

Figure S20. LC–HRMS/MS spectra of $[M+H]^+$ of unidentified microcystin-**35**, and MC-LA (**41**).

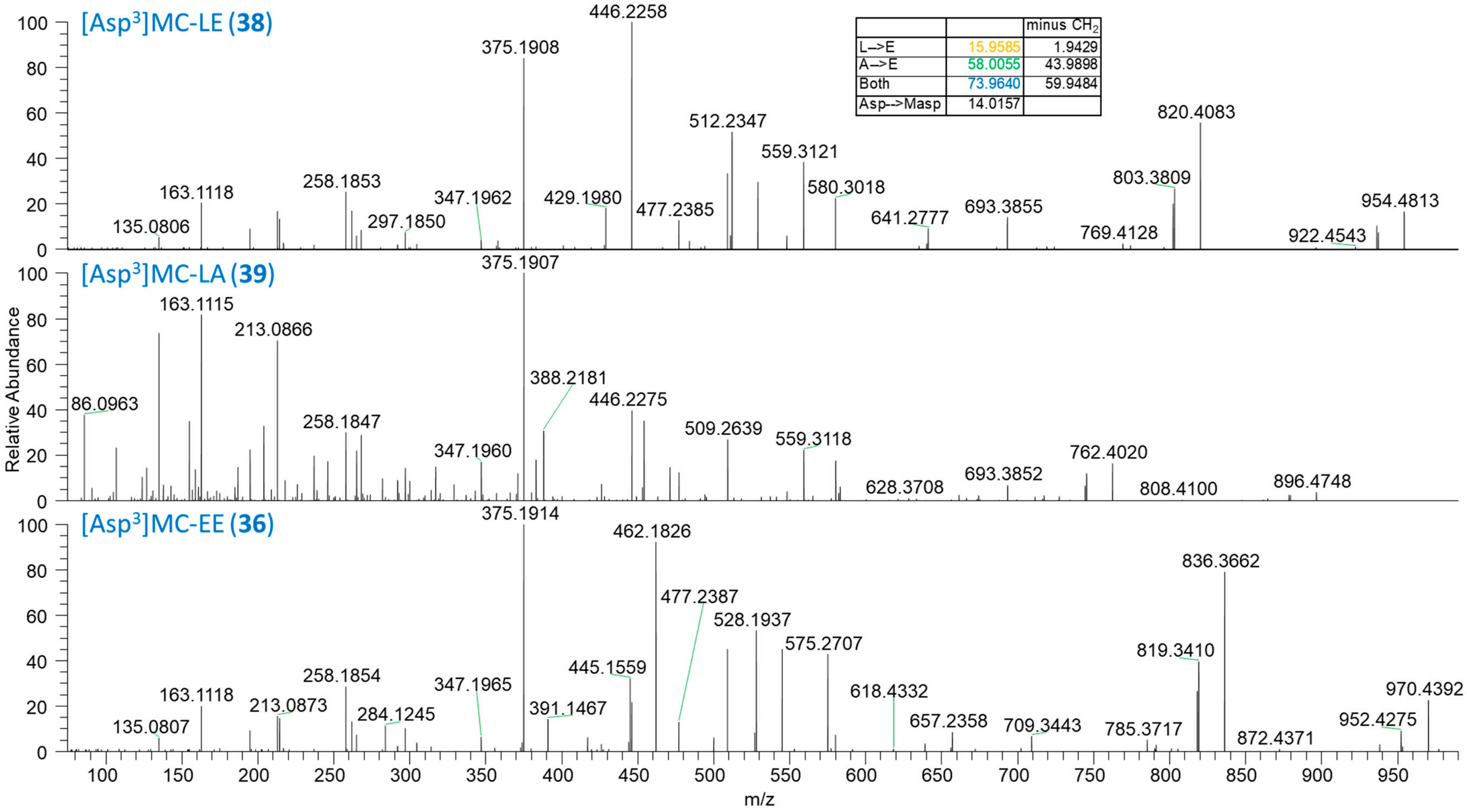

		minus CH_2
L–>E	15.9585	1.9429
A–>E	58.0055	43.9898
Both	73.9640	59.9484
Asp–>Masp	14.0157	

Figure S21. LC–HRMS/MS spectra of [M+H]+ of [D-Asp3]MC-LE (**38**), [D-Asp3]MC-LA (**39**), and [D-Asp3]MC-EE (**36**).

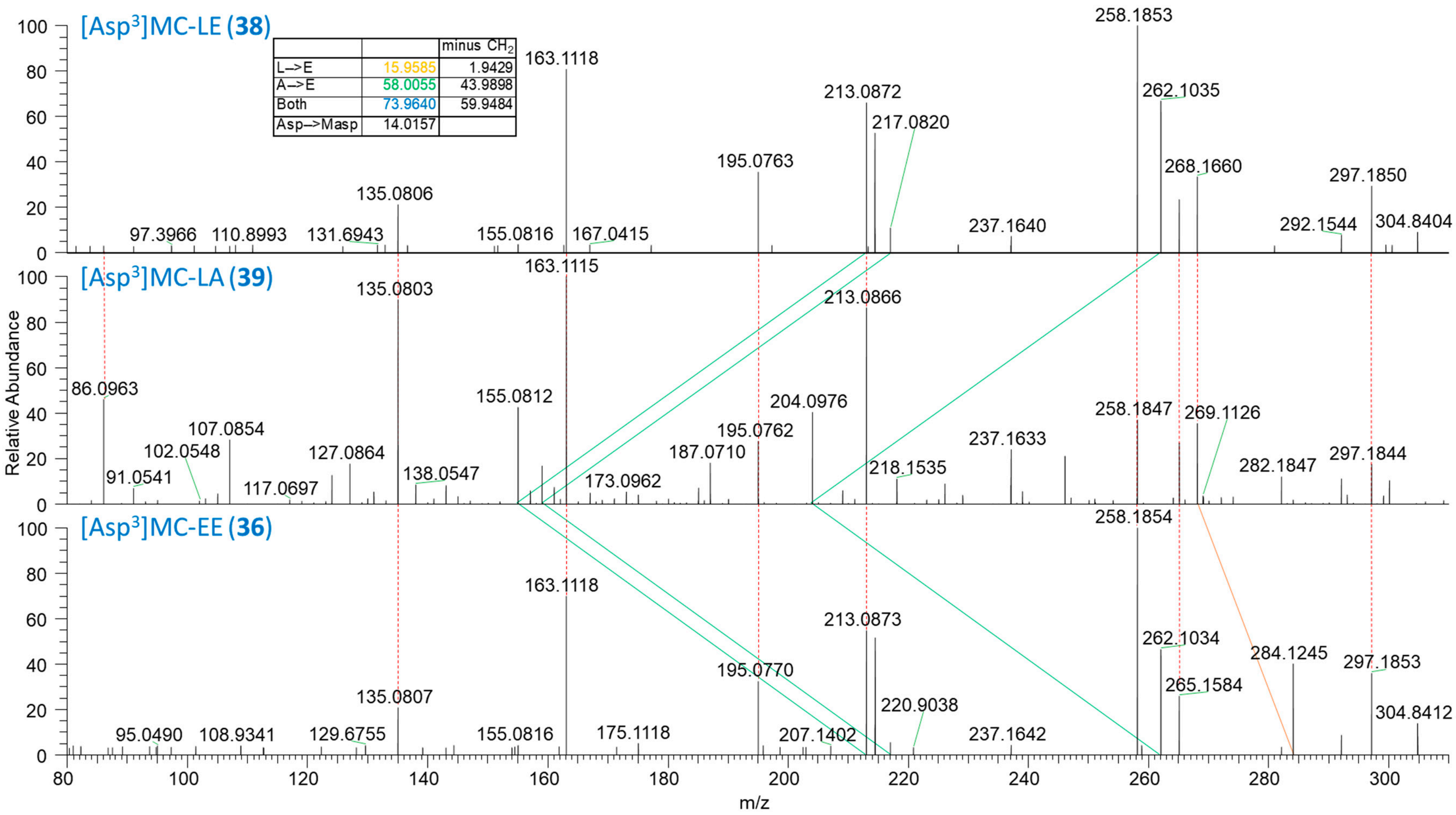

Figure S22. Expansion of LC–HRMS/MS spectra of [M+H]+ of [D-Asp3]MC-LE (**38**), [D-Asp3]MC-LA (**39**), and [D-Asp3]MC-EE (**36**).

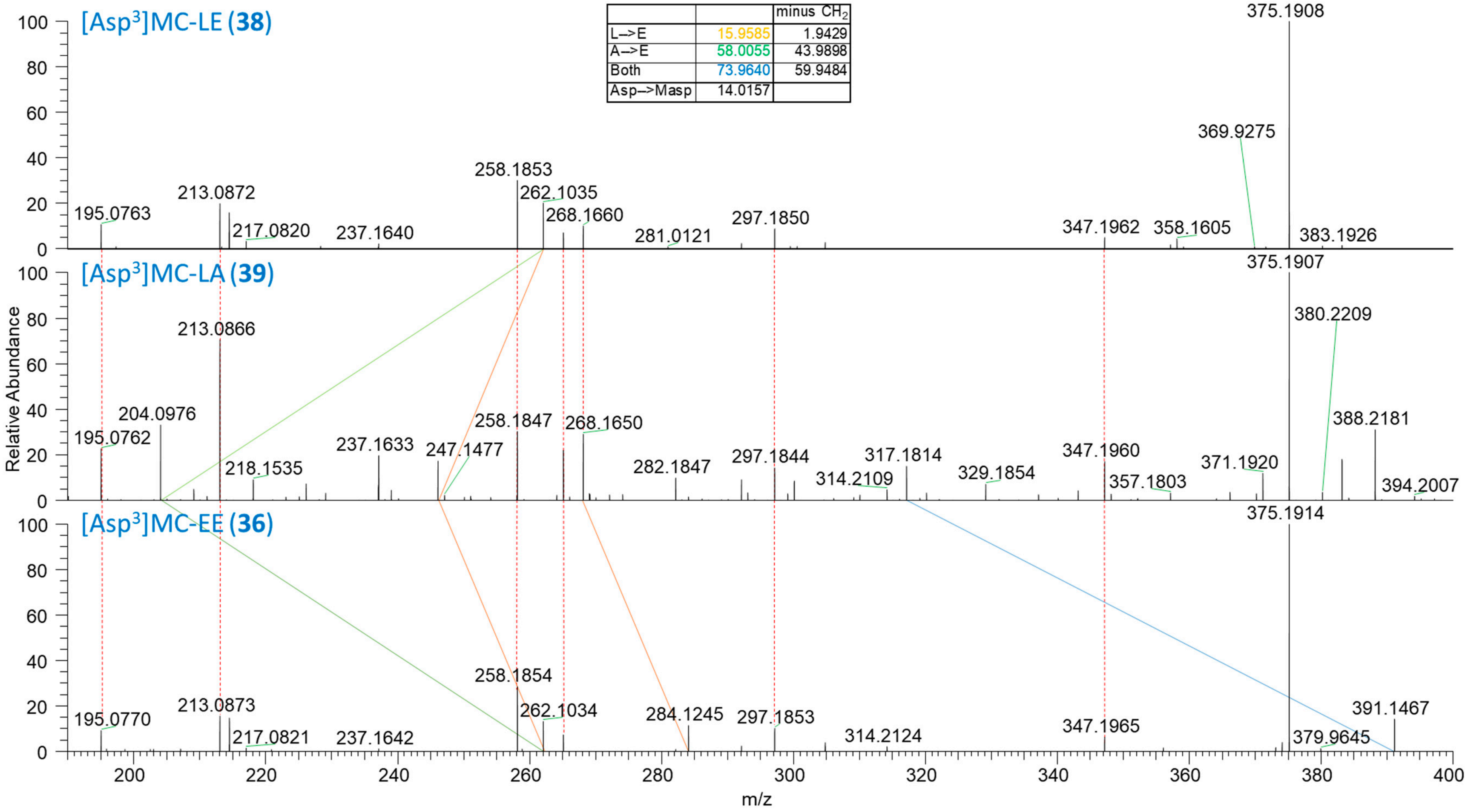

Figure S23. Expansion of LC–HRMS/MS spectra of $[M+H]^+$ of [D-Asp³]MC-LE (**38**), [D-Asp³]MC-LA (**39**), and [D-Asp³]MC-EE (**36**).

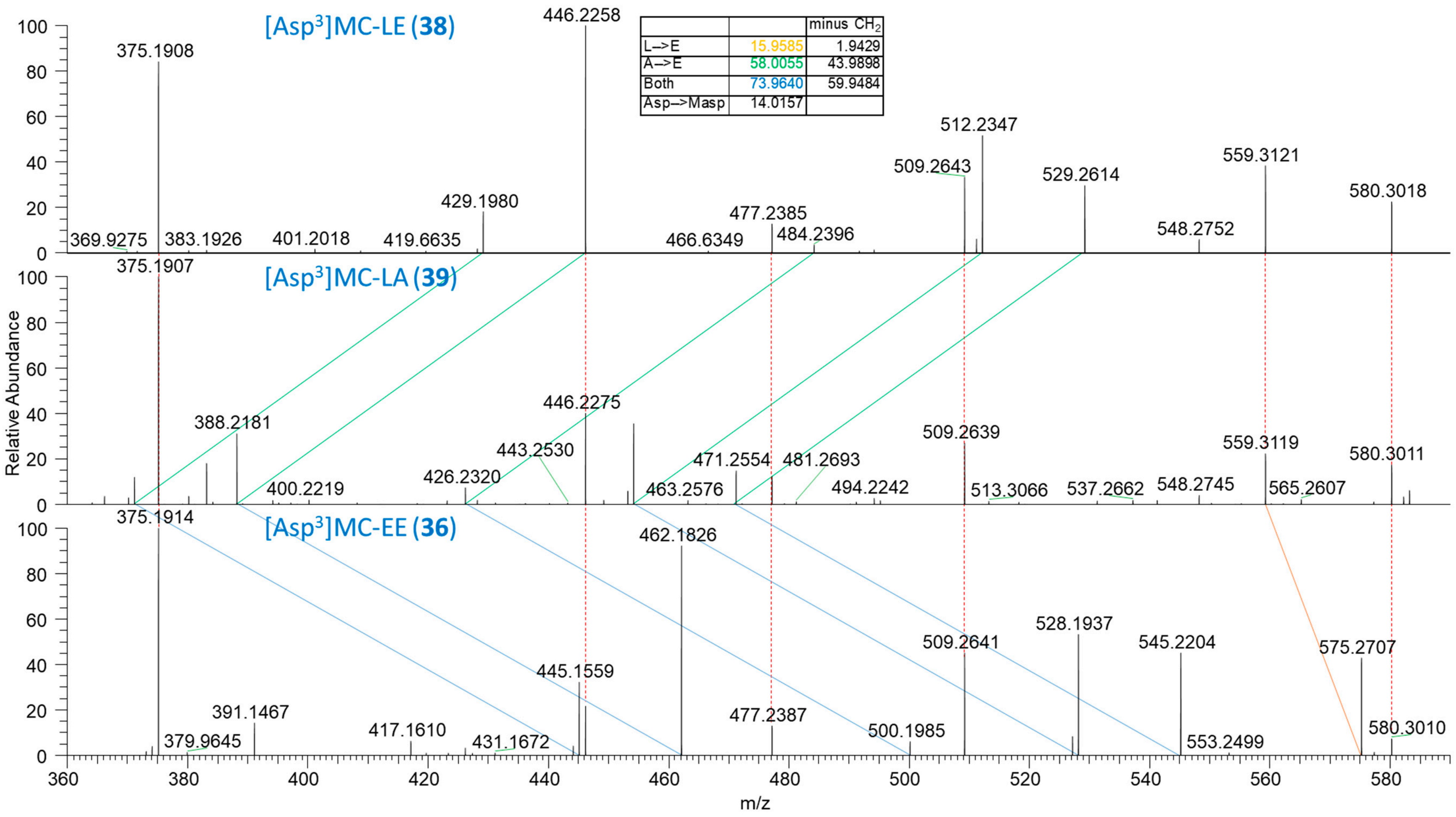

Figure S24. Expansion of LC–HRMS/MS spectra of $[M+H]^+$ of [D-Asp^3]MC-LE (**38**), [D-Asp^3]MC-LA (**39**), and [D-Asp^3]MC-EE (**36**).

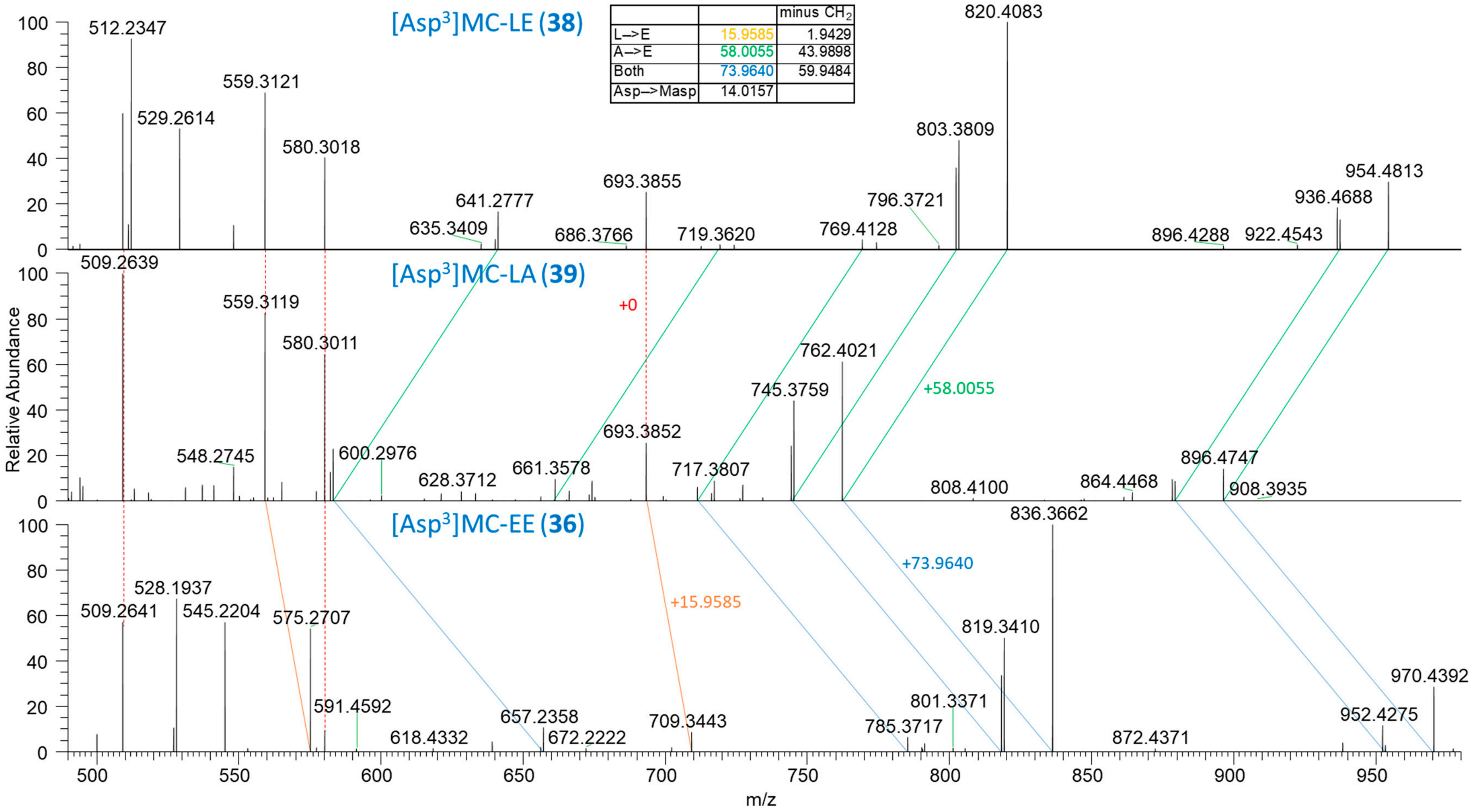

Figure S25. Expansion of LC–HRMS/MS spectra of [M+H]+ of [D-Asp3]MC-LE (**38**), [D-Asp3]MC-LA (**39**), and [D-Asp3]MC-EE (**36**).

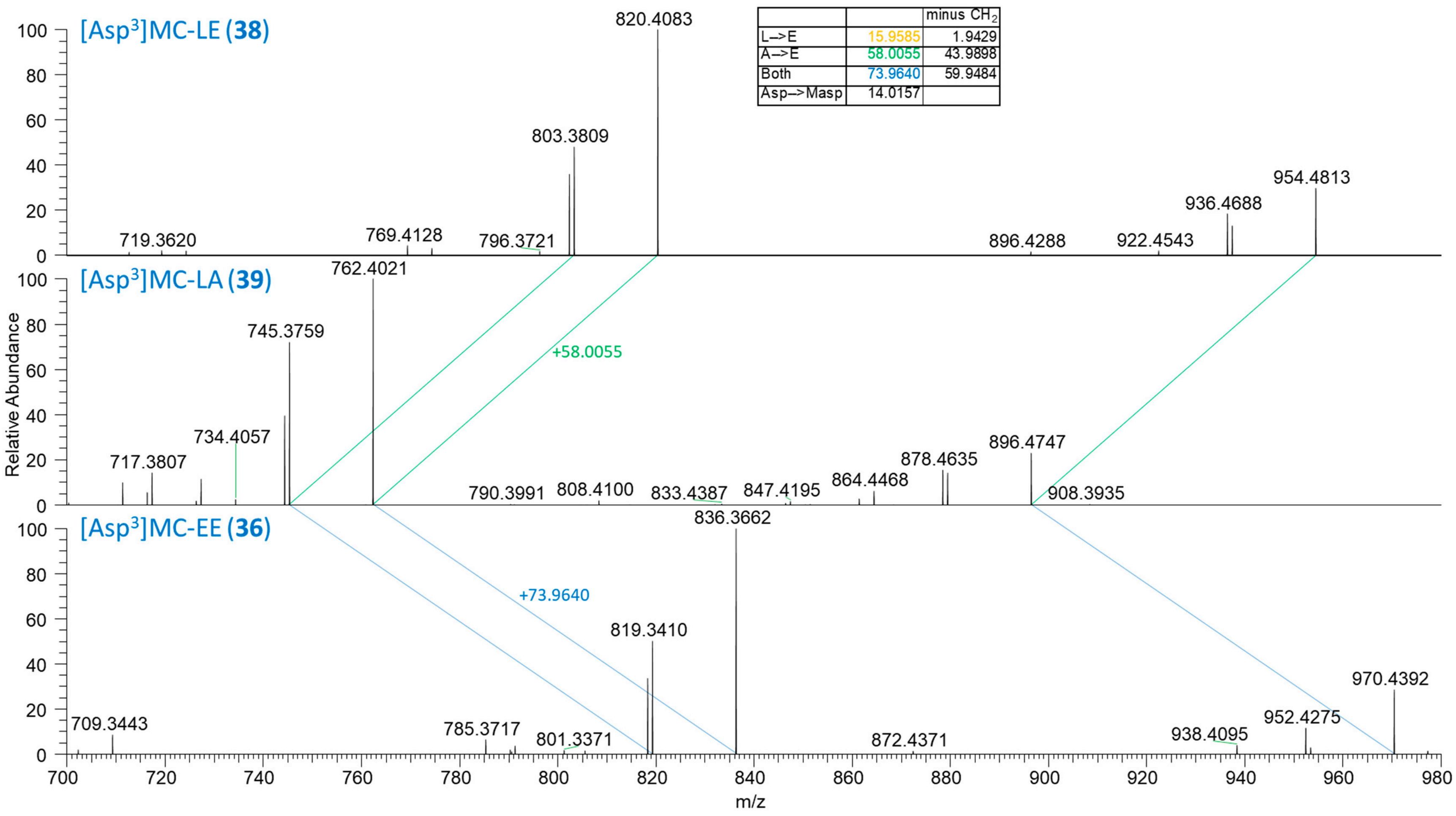

Figure S26. Expansion of LC–HRMS/MS spectra of [M+H]+ of [D-Asp3]MC-LE (**38**), [D-Asp3]MC-LA (**39**), and [D-Asp3]MC-EE (**36**).

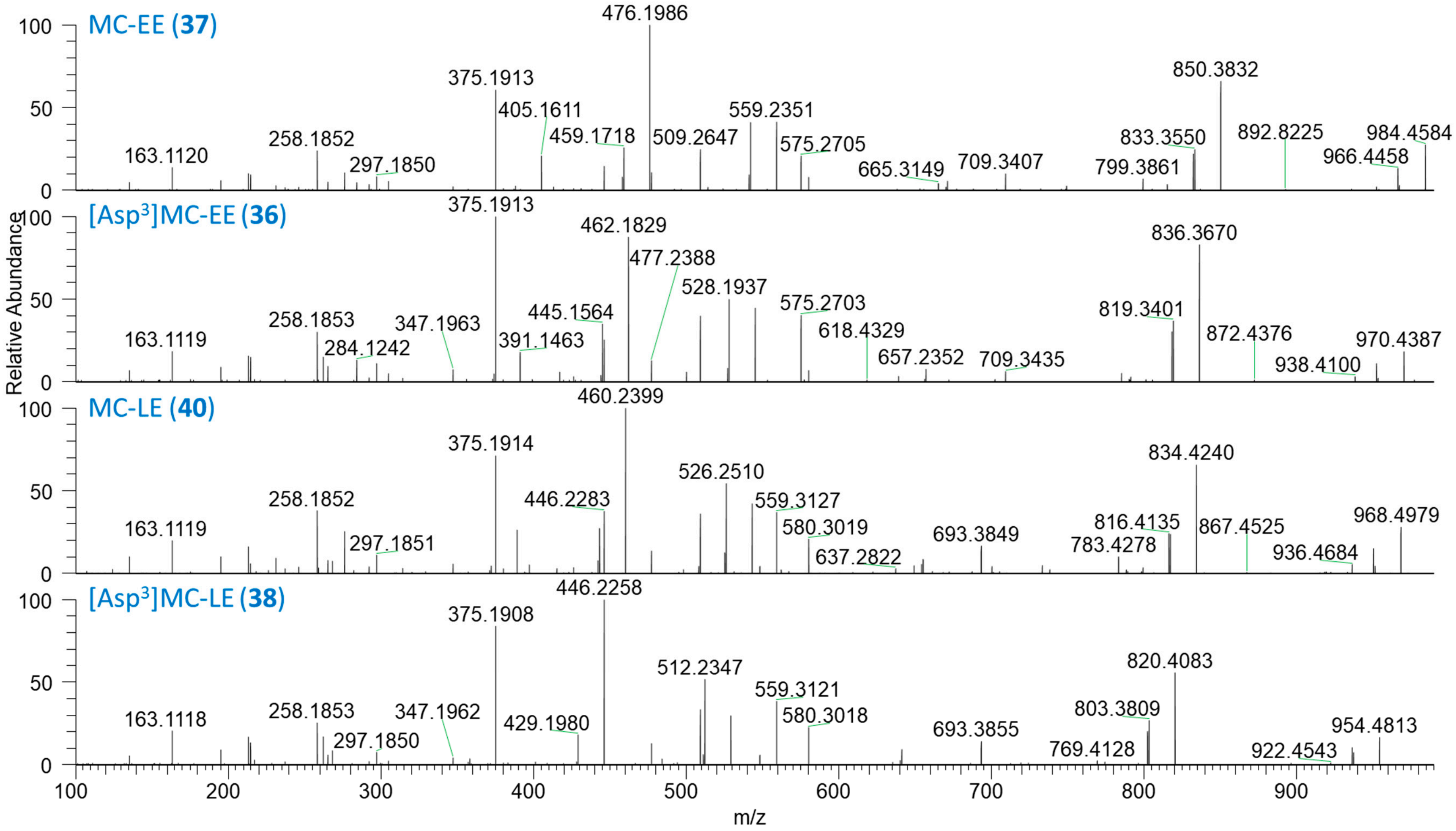

Figure S27. LC–HRMS/MS spectra of [M+H]+ of MC-EE (**37**), [D-Asp3]MC-EE (**36**), MC-LE (**40**), and [D-Asp3]MC-LE (**38**).

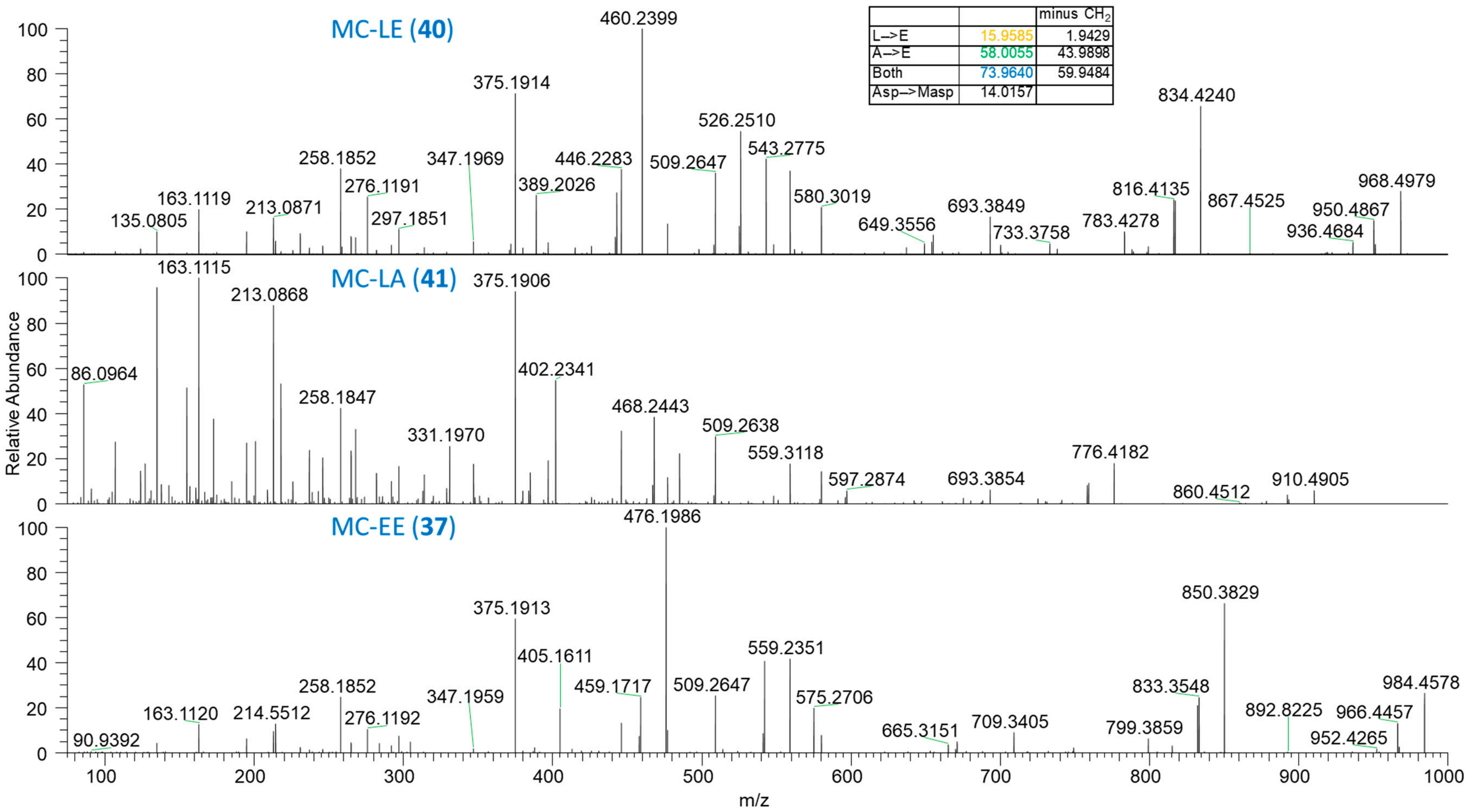

Figure S28. LC–HRMS/MS spectra of [M+H]⁺ of MC-LE (**40**), MC-LA (**41**), and MC-EE (**37**).

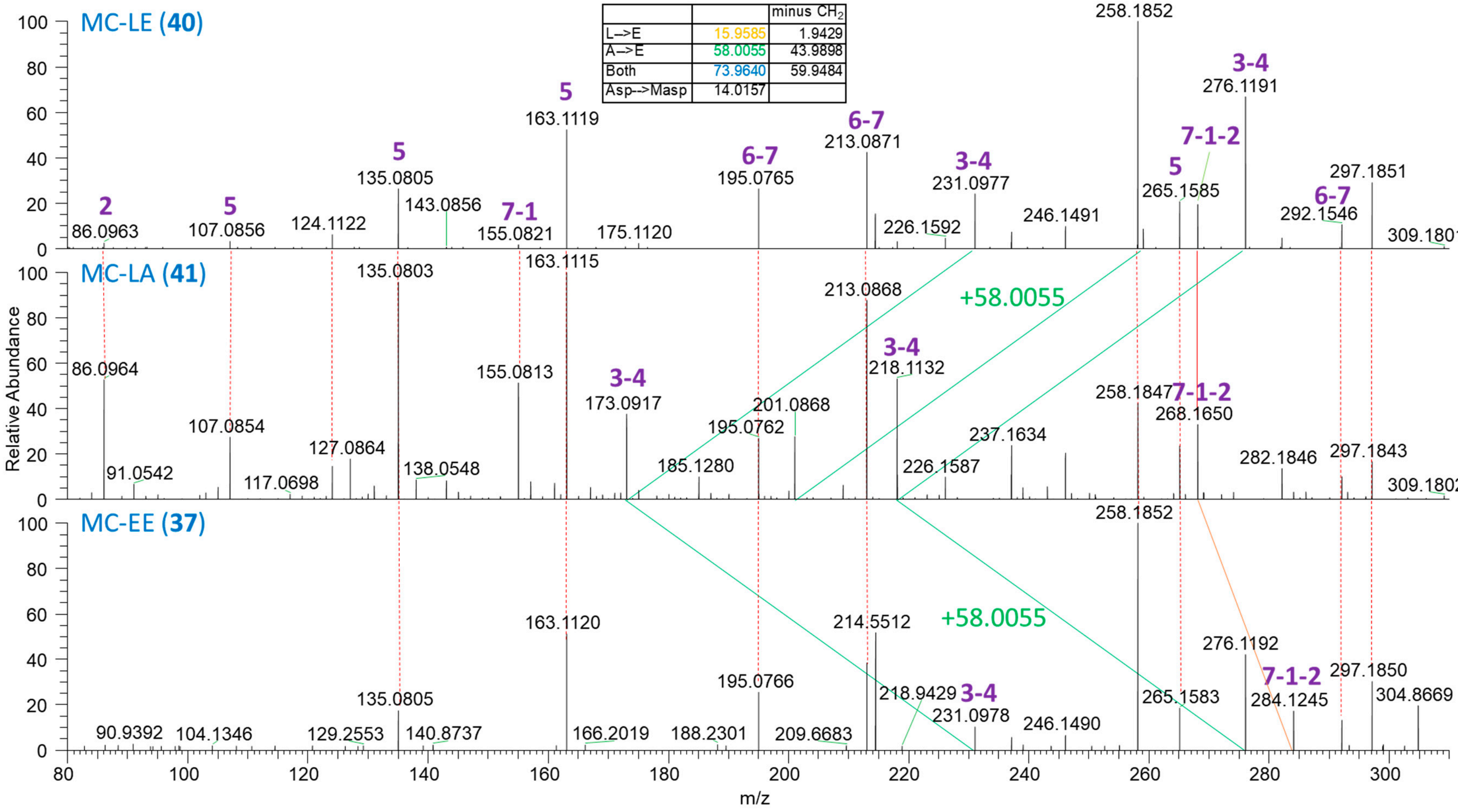

Figure S29. Expansion of LC–HRMS/MS spectra of $[M+H]^+$ of MC-LE (**40**), MC-LA (**41**), and MC-EE (**37**).

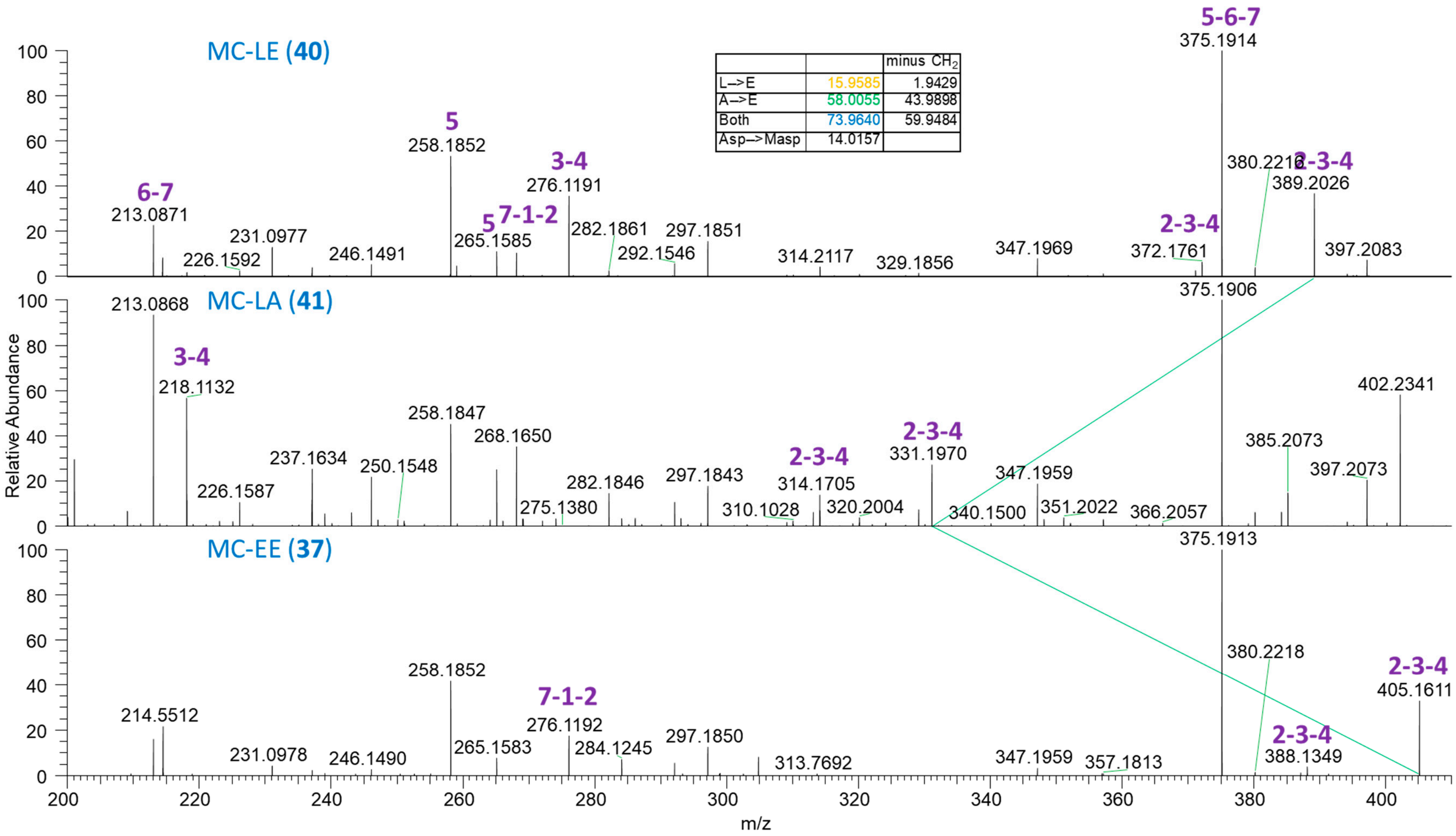

Figure S30. Expansion of LC–HRMS/MS spectra of [M+H]⁺ of MC-LE (**40**), MC-LA (**41**), and MC-EE (**37**).

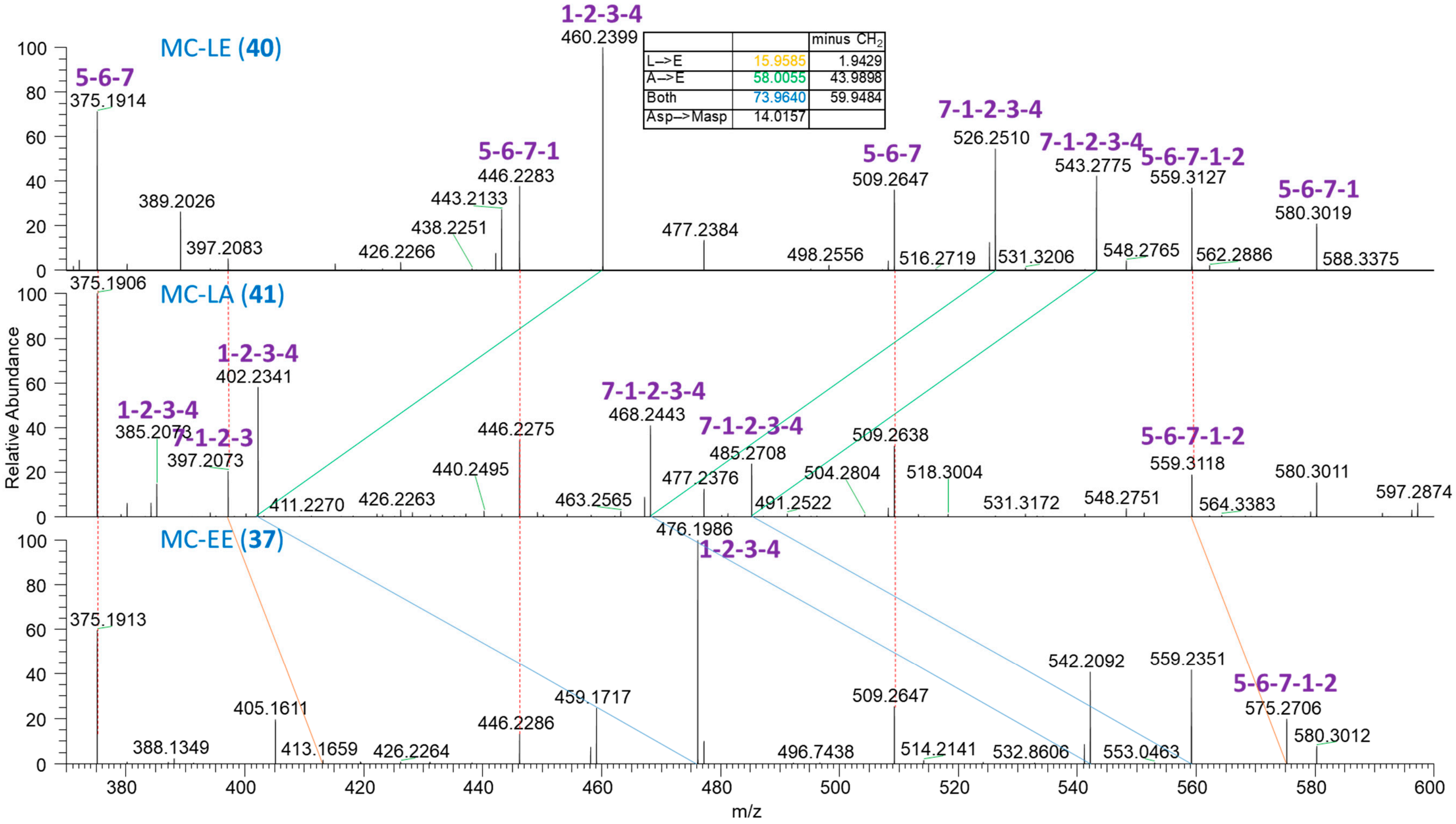

Figure S31. Expansion of LC–HRMS/MS spectra of [M+H]+ of MC-LE (**40**), MC-LA (**41**), and MC-EE (**37**).

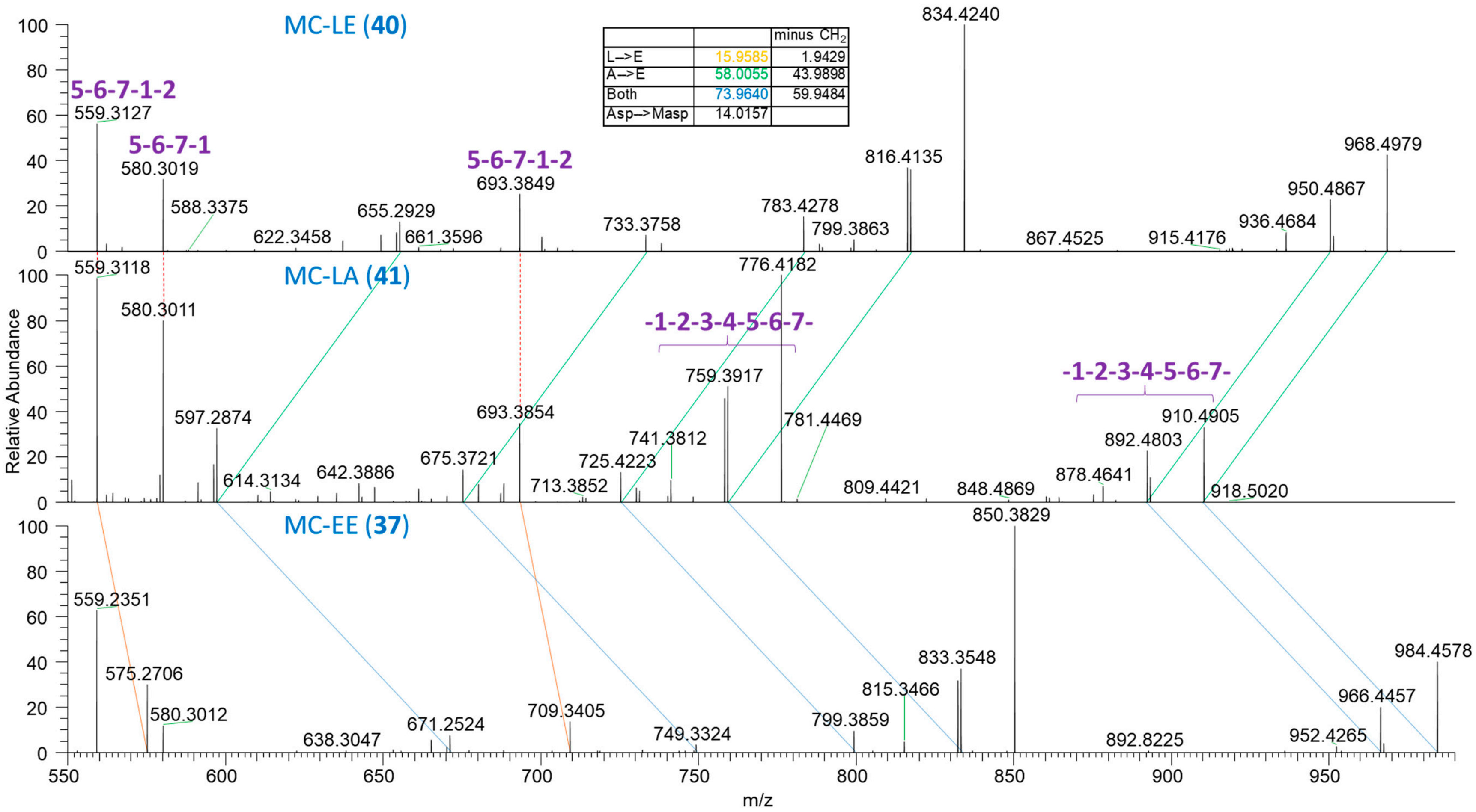

Figure S32. Expansion of LC–HRMS/MS spectra of $[M+H]^+$ of MC-LE (**40**), MC-LA (**41**), and MC-EE (**37**).

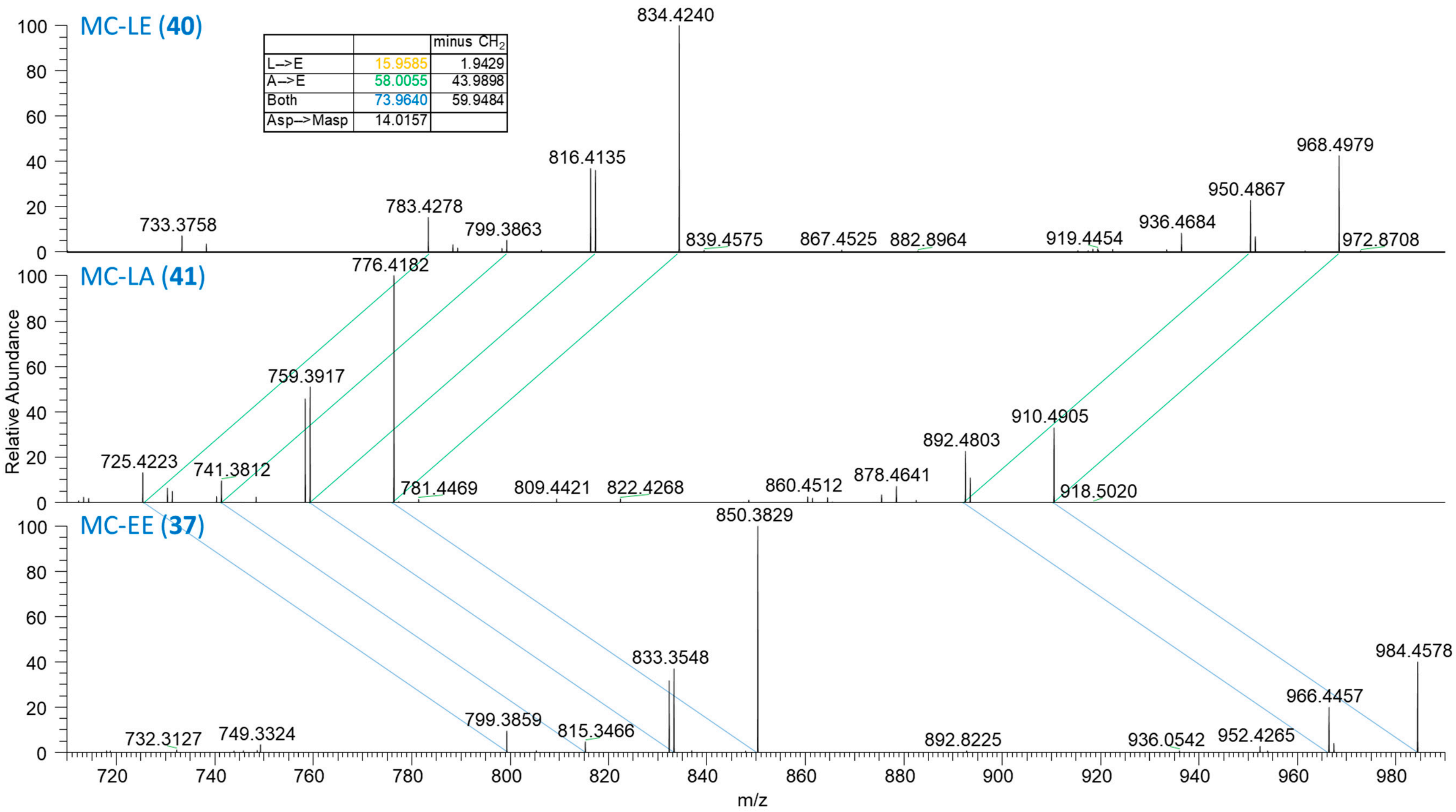

Figure S33. Expansion of LC–HRMS/MS spectra of $[M+H]^+$ of MC-LE (**40**), MC-LA (**41**), and MC-EE (**37**).

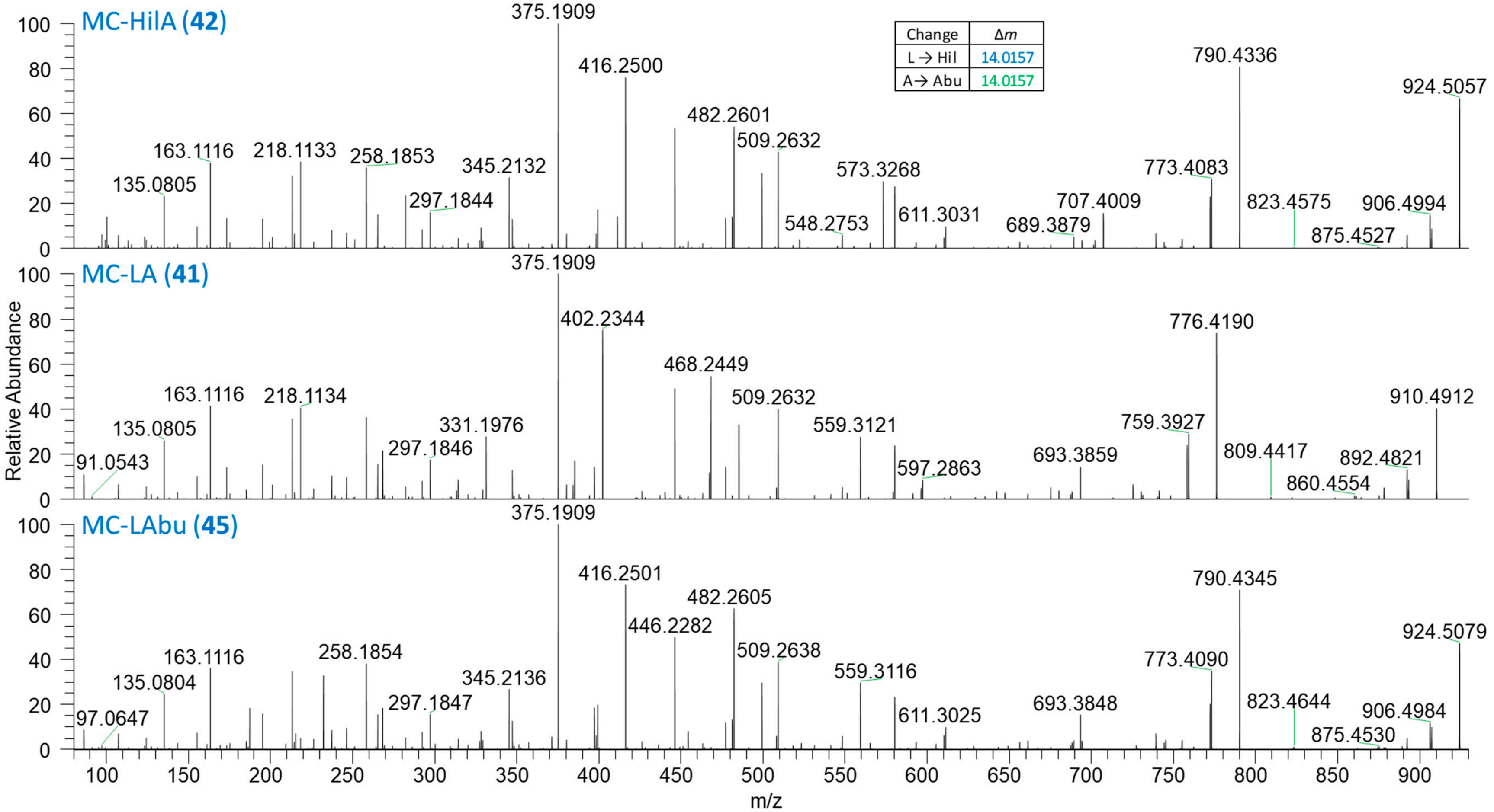

Figure S34. LC–HRMS/MS spectra of [M+H]$^+$ of MC-HilA (**42**), MC-LA (**41**), and MC-LAbu (**45**).

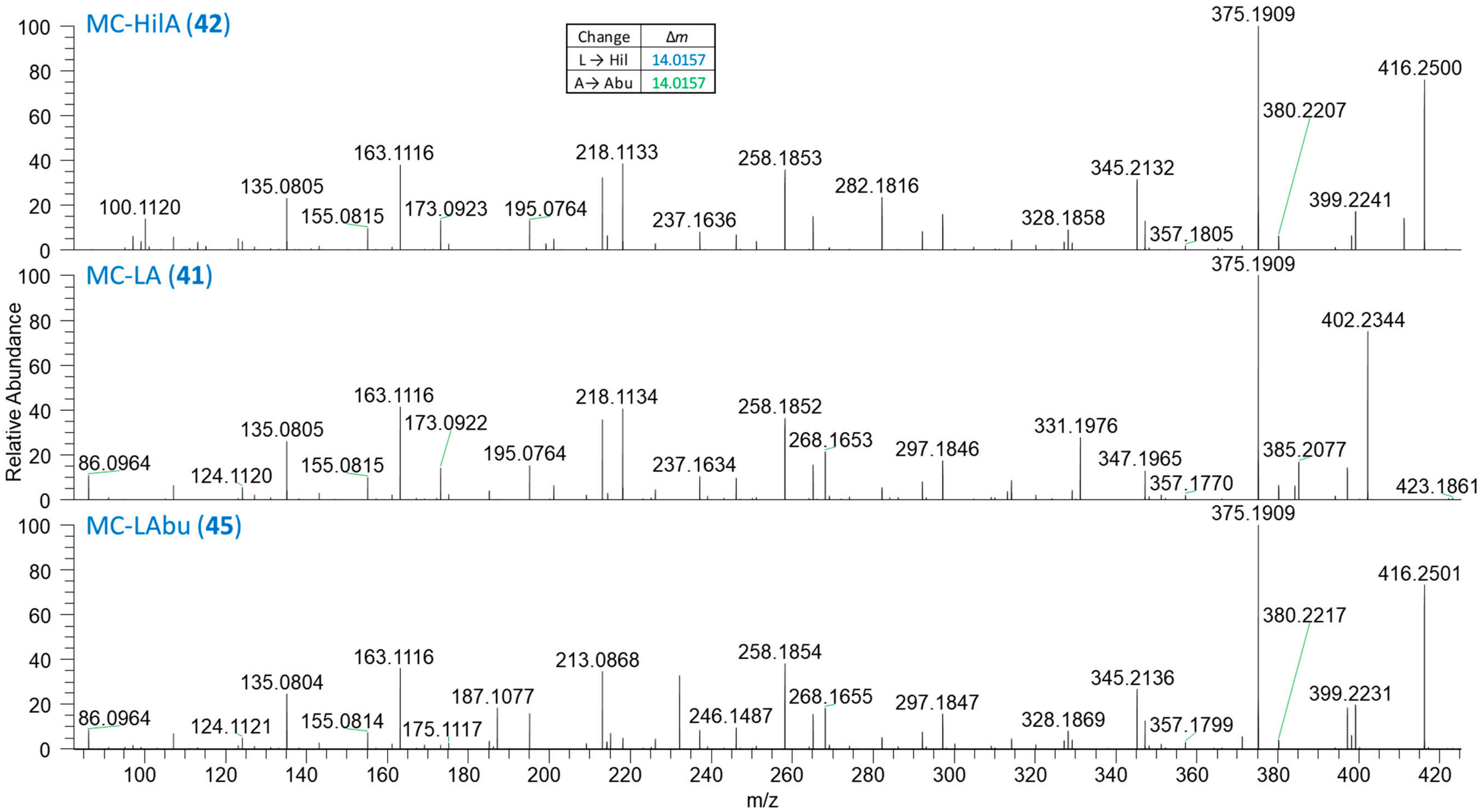

Figure S35. Expansion of LC–HRMS/MS spectra of [M+H]+ of MC-HilA (**42**), MC-LA (**41**), and MC-LAbu (**45**).

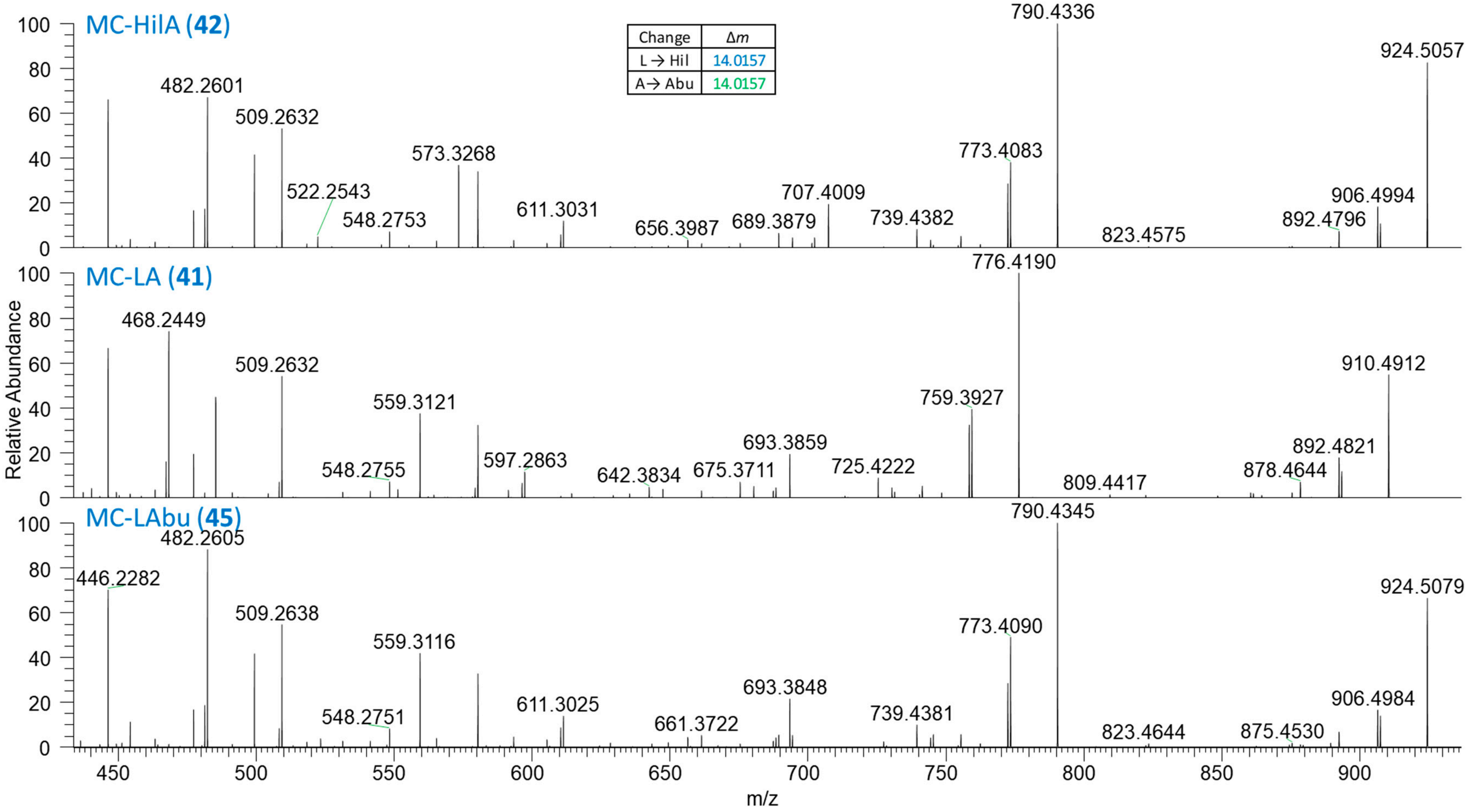

Figure S36. Expansion of LC–HRMS/MS spectra of [M+H]+ of MC-HilA (**42**), MC-LA (**41**), and MC-LAbu (**45**).

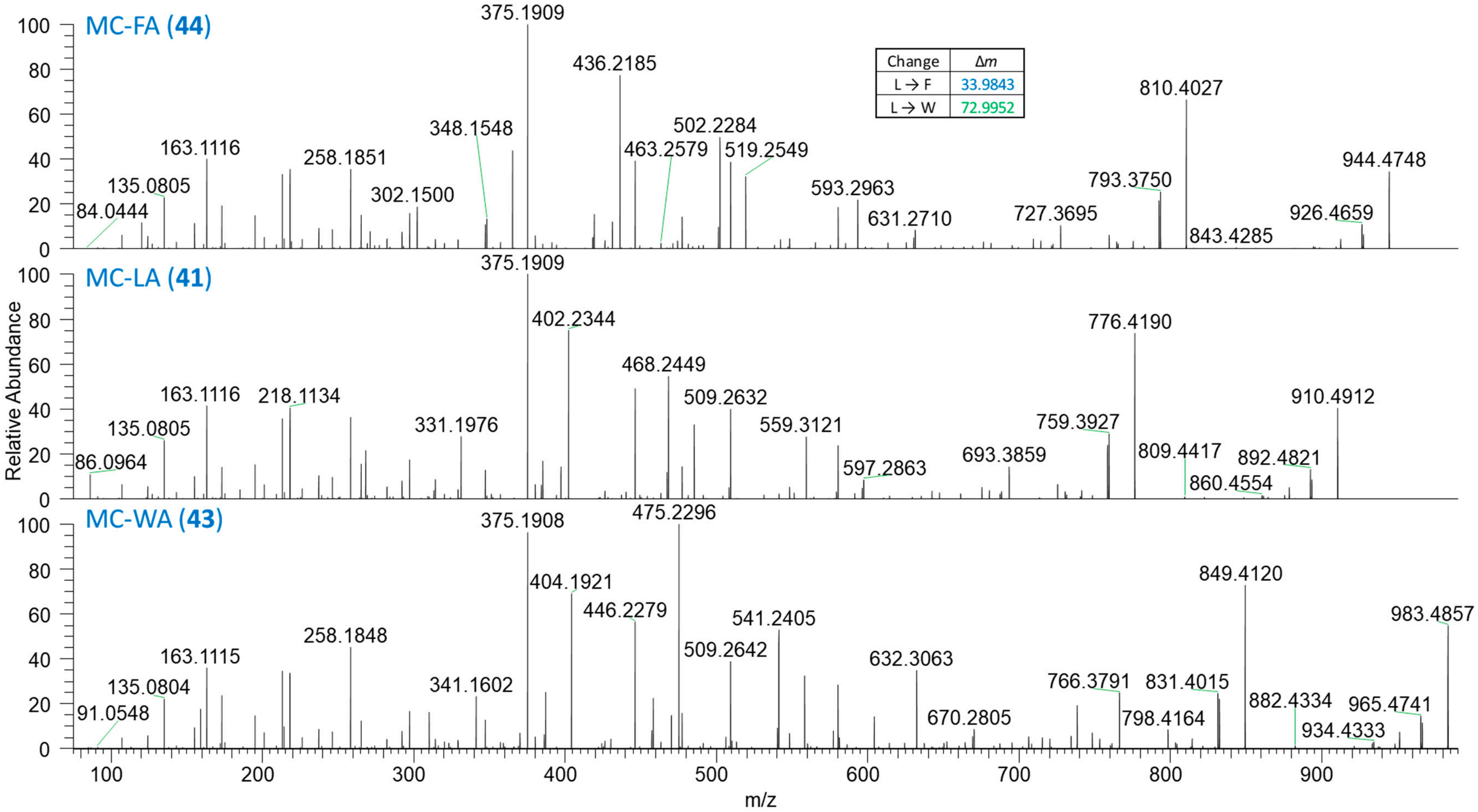

Figure S37. LC–HRMS/MS spectra of $[M+H]^+$ of MC-FA (**44**), MC-LA (**41**), and MC-WA (**43**).

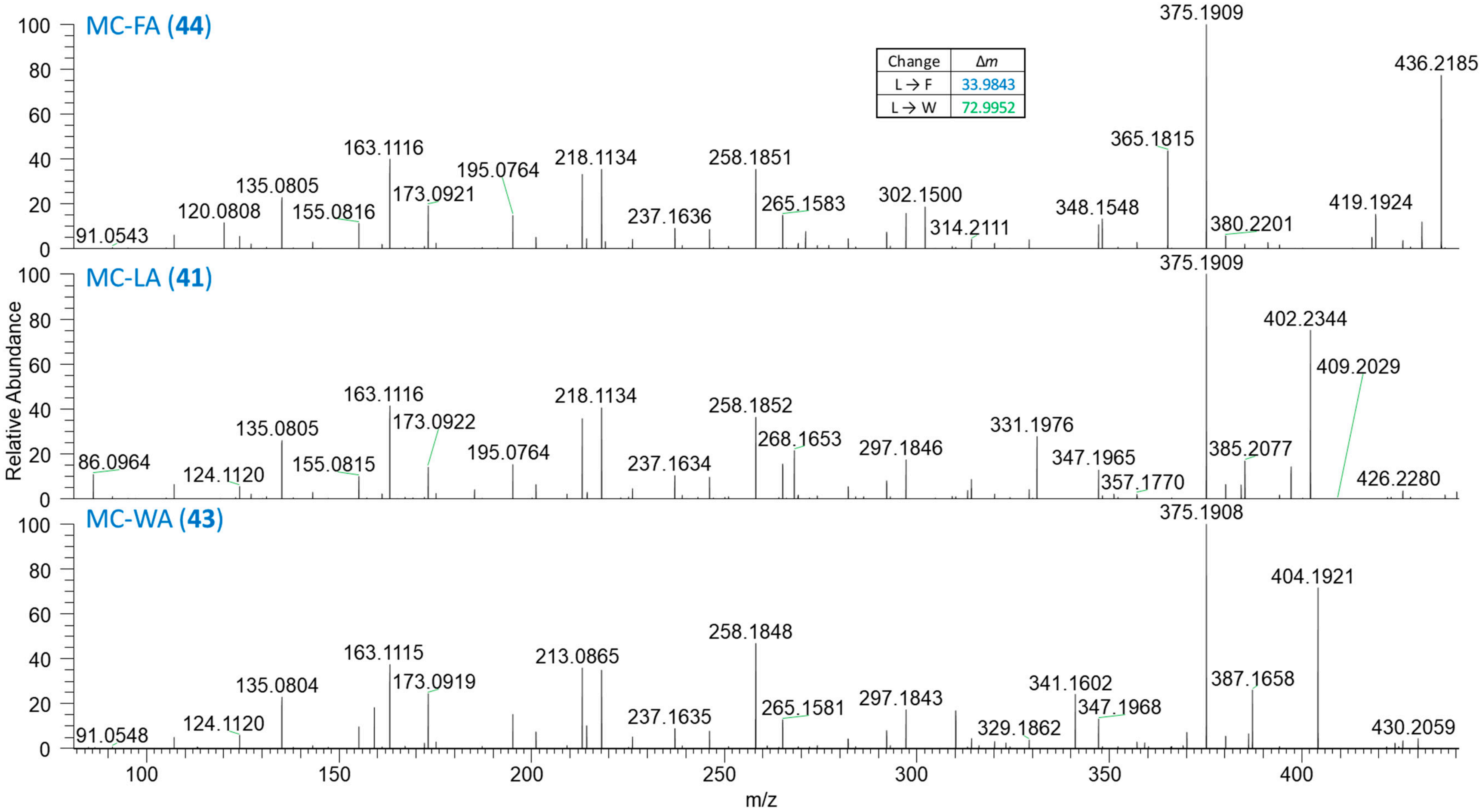

Figure S38. Expansion of LC–HRMS/MS spectra of [M+H]$^+$ of MC-FA (**44**), MC-LA (**41**), and MC-WA (**43**).

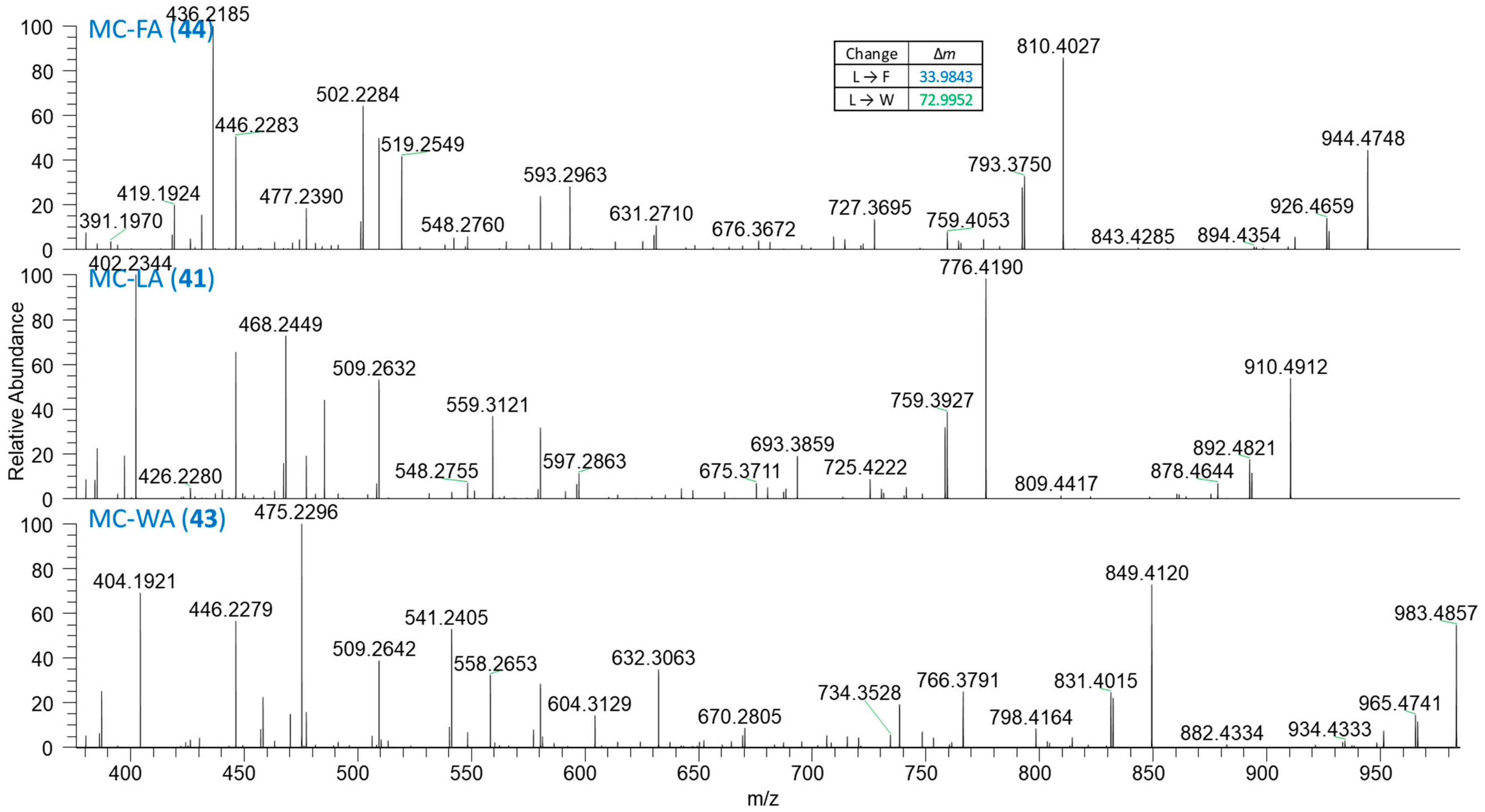

Figure S39. Expansion of LC–HRMS/MS spectra of [M+H]$^+$ of MC-FA (**44**), MC-LA (**41**), and MC-WA (**43**).

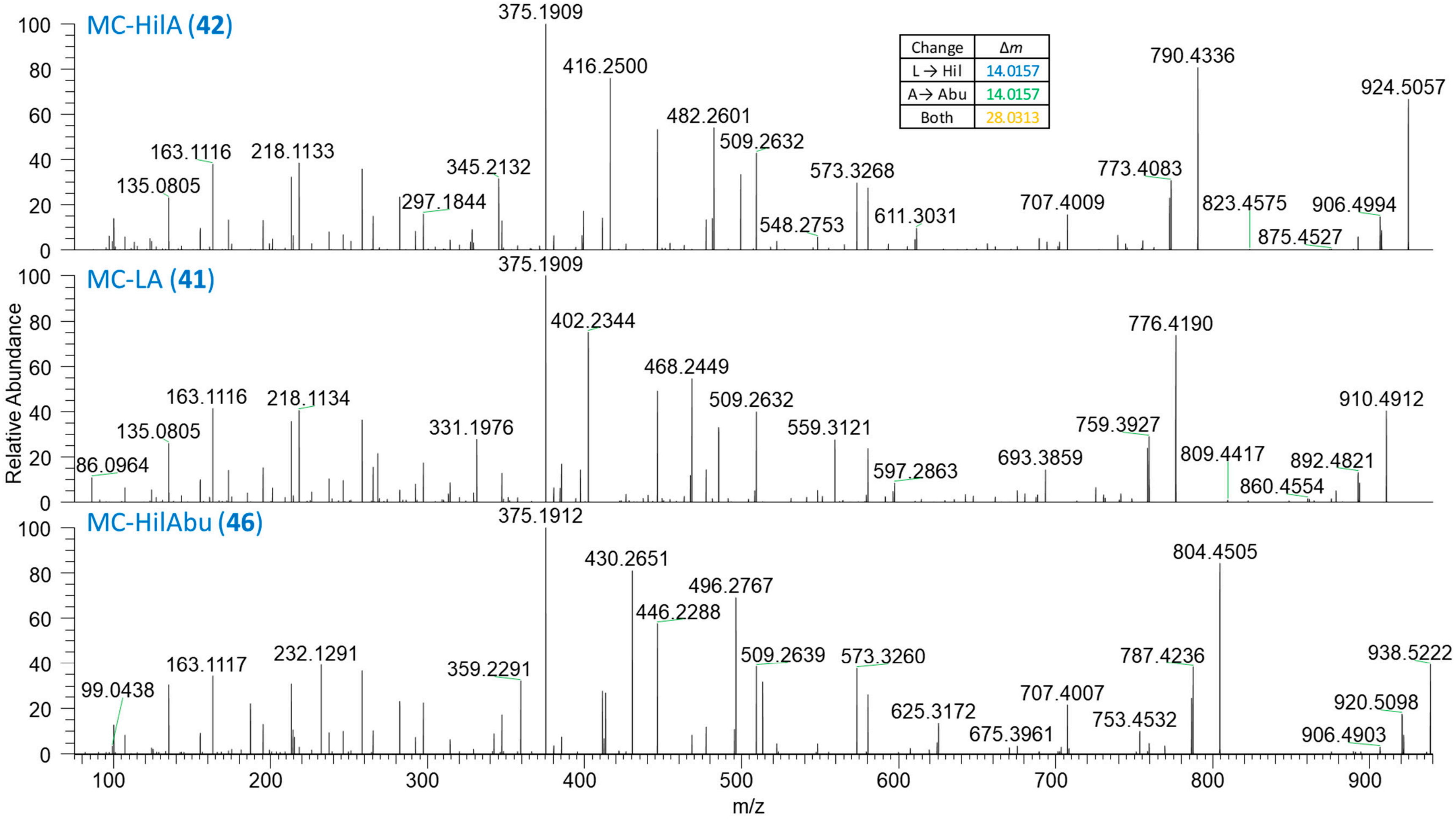

Figure S40. LC–HRMS/MS spectra of $[M+H]^+$ of MC-HilA (**42**), MC-LA (**41**), and MC-HilAbu (**46**).

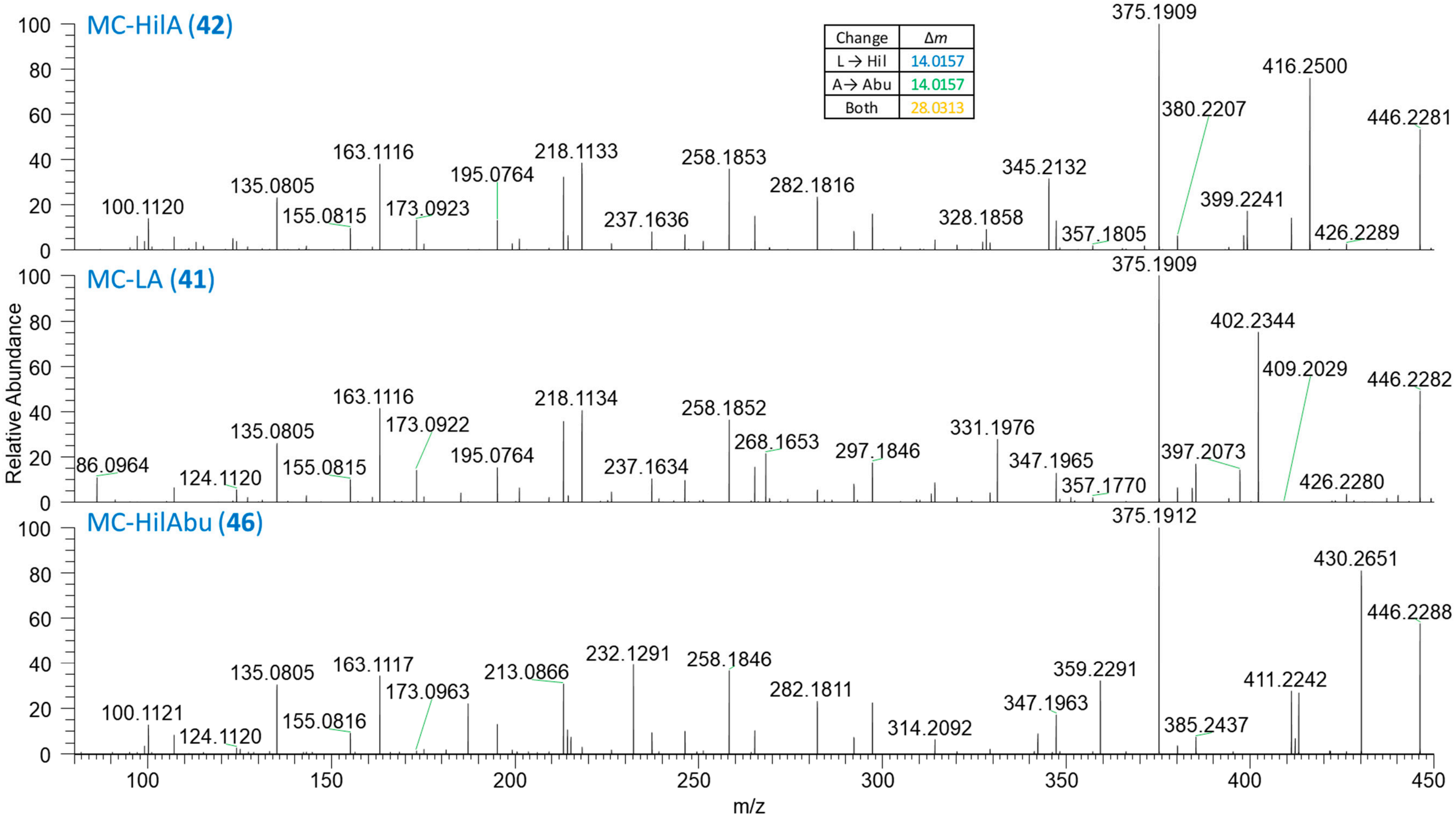

Figure S41. Expansion of LC–HRMS/MS spectra of [M+H]+ of MC-HilA (**42**), MC-LA (**41**), and MC-HilAbu (**46**).

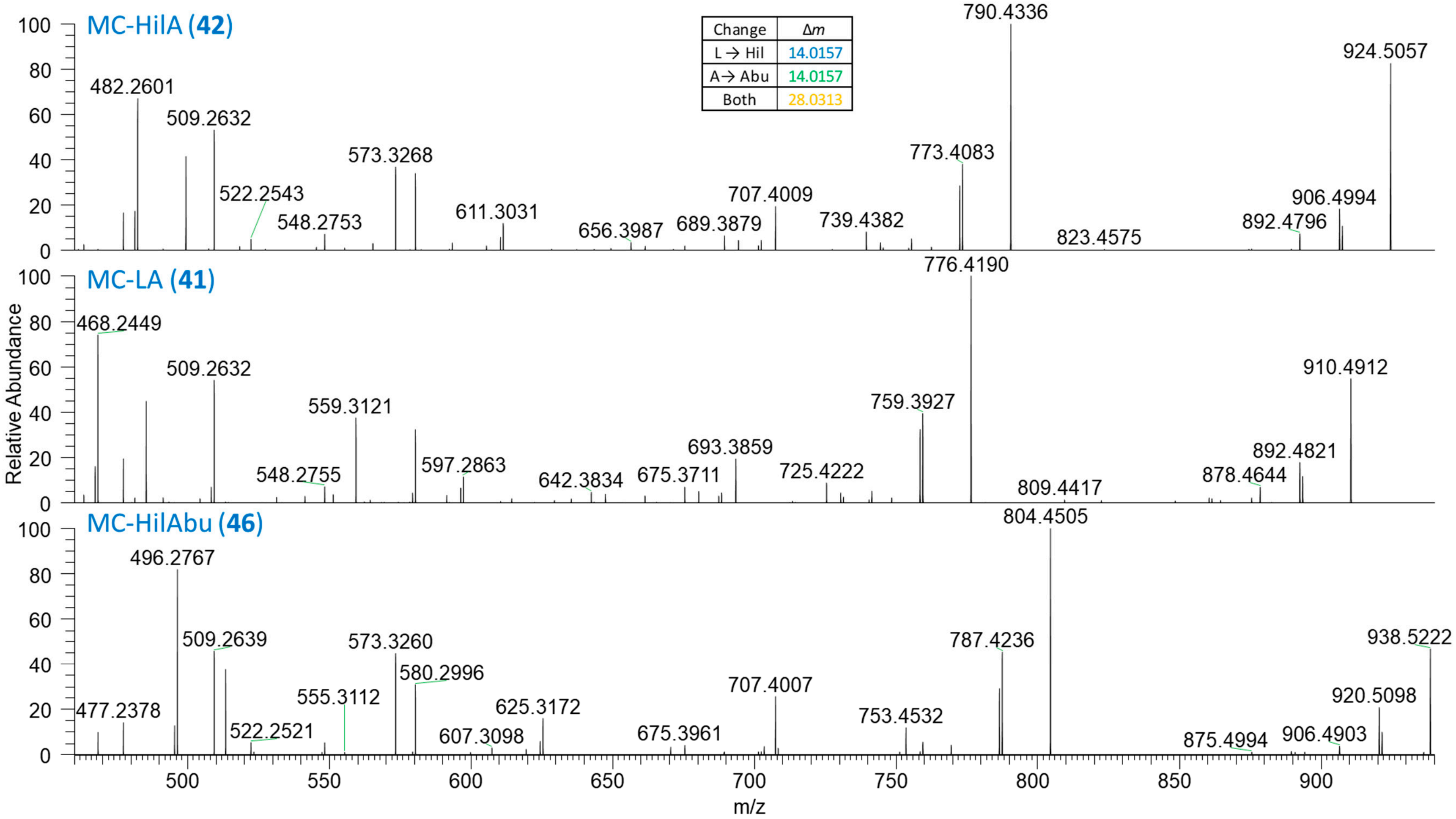

Figure S42. Expansion of LC–HRMS/MS spectra of $[M+H]^+$ of MC-HilA (**42**), MC-LA (**41**), and MC-HilAbu (**46**).

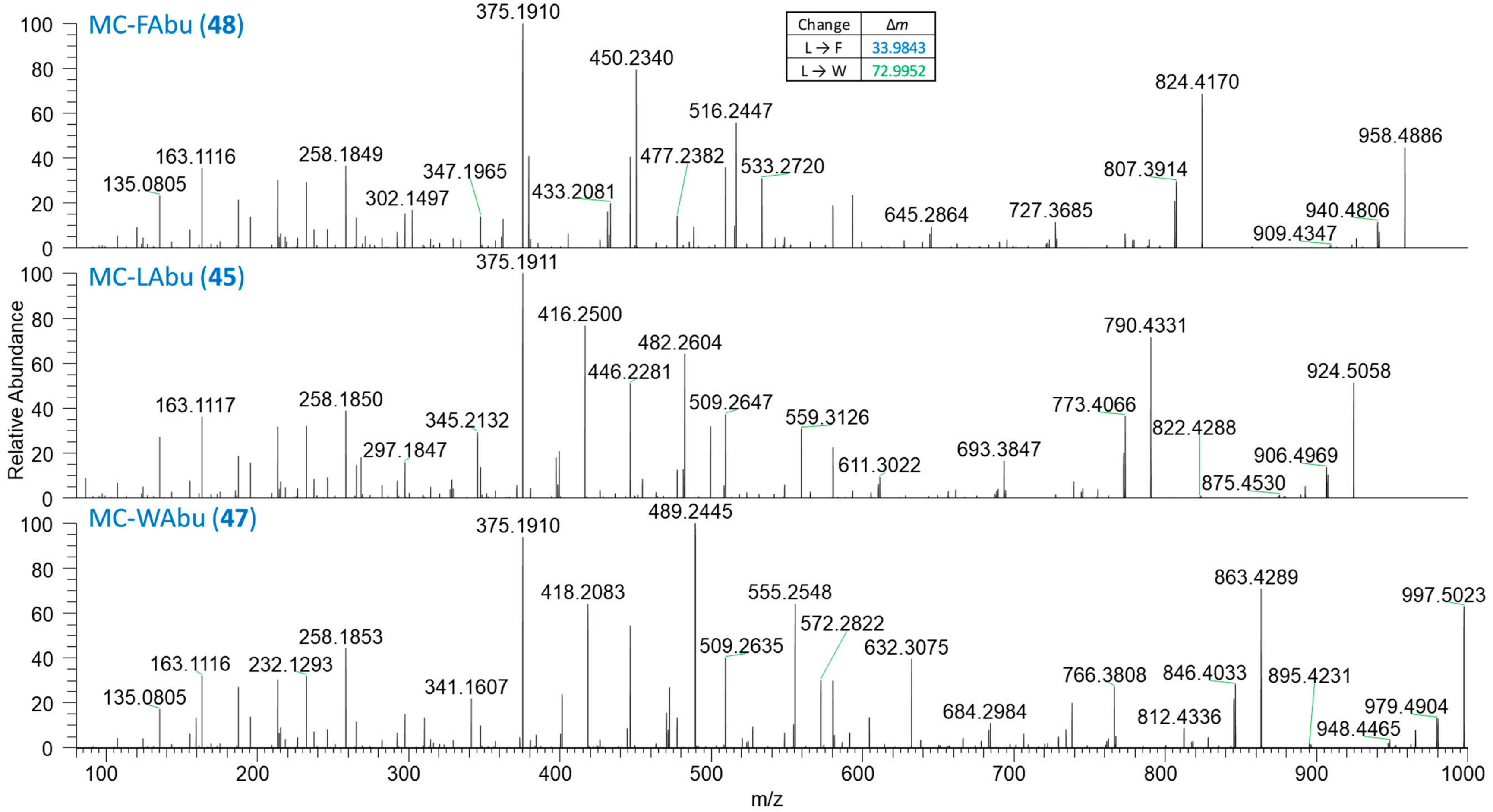

Figure S43. LC–HRMS/MS spectra of $[M+H]^+$ of MC-FAbu (**48**), MC-LAbu (**45**), and MC-WAbu (**47**).

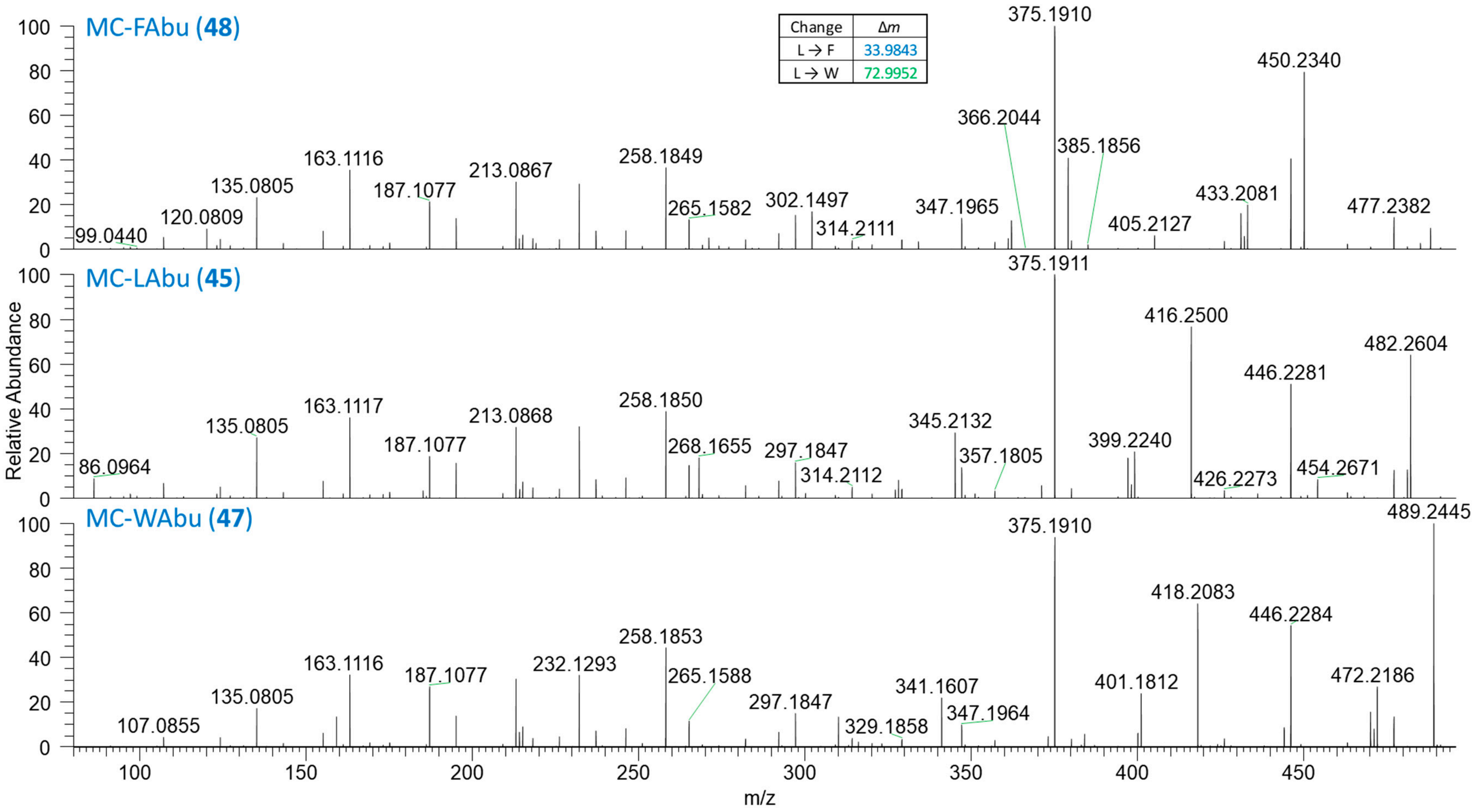

Figure S44. Expansion of LC–HRMS/MS spectra of [M+H]+ of MC-FAbu (**48**), MC-LAbu (**45**), and MC-WAbu (**47**).

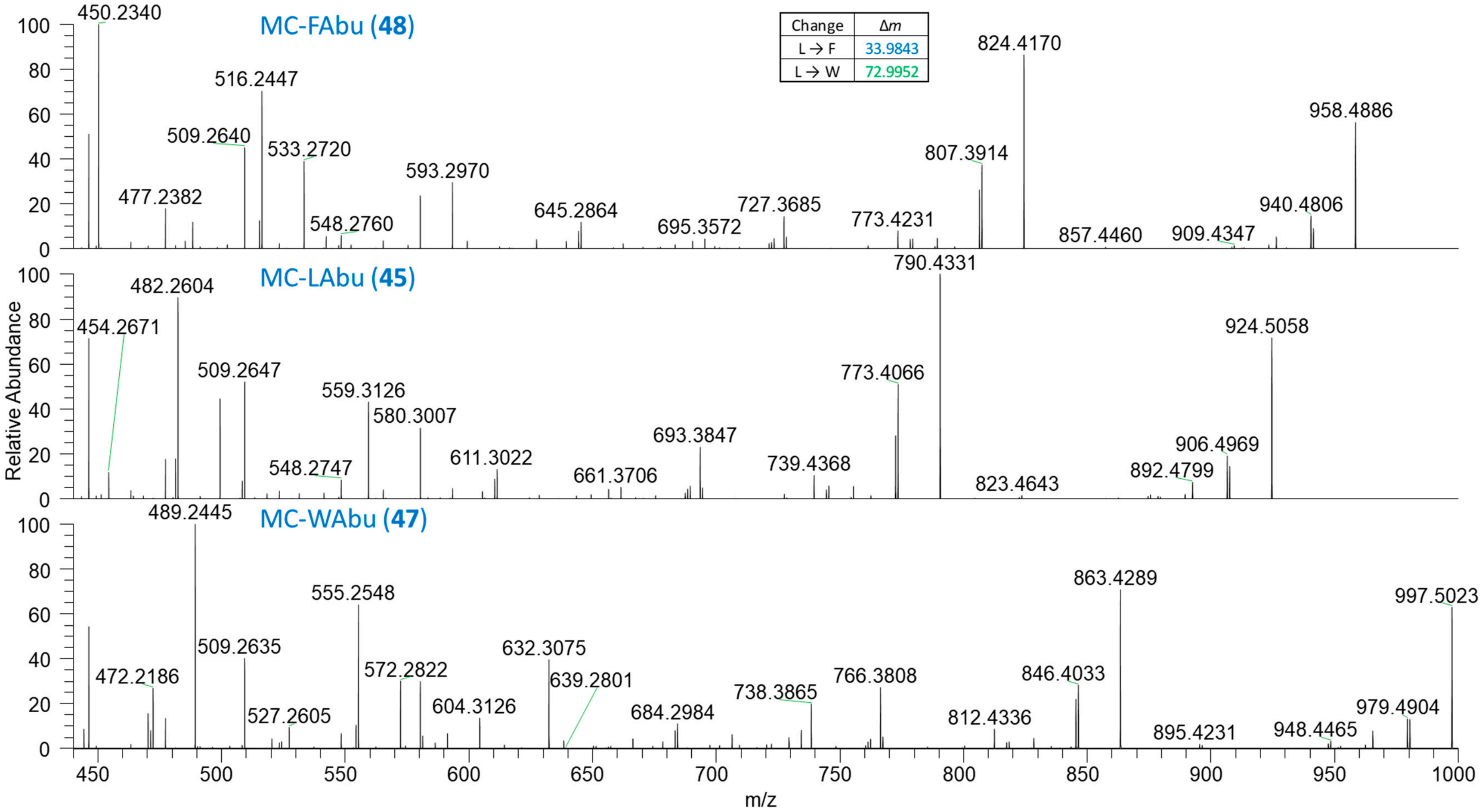

Figure S45. Expansion of LC–HRMS/MS spectra of [M+H]⁺ of MC-FAbu (**48**), MC-LAbu (**45**), and MC-WAbu (**47**).

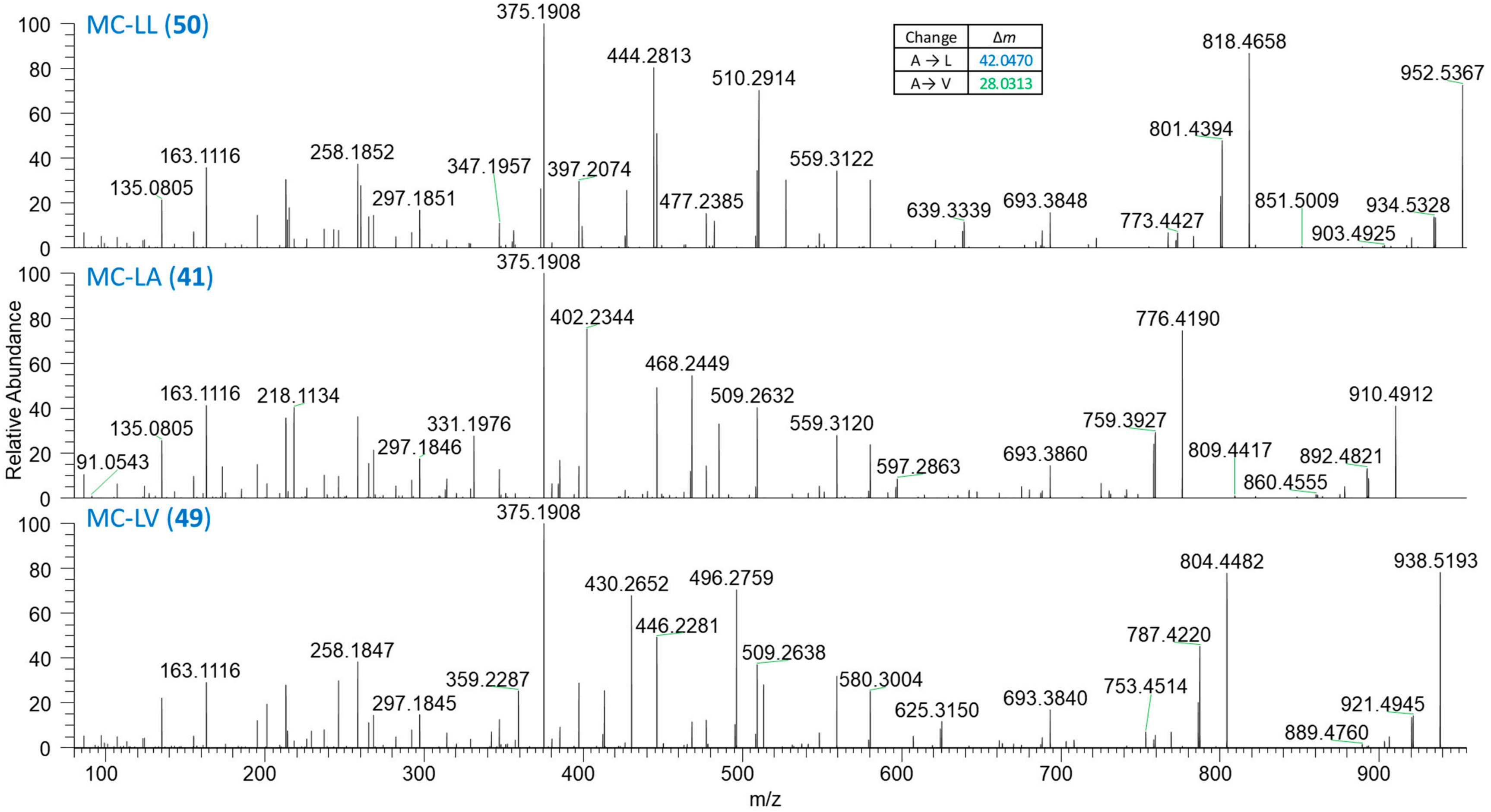

Figure S46. LC–HRMS/MS spectra of [M+H]+ of MC-LL (**50**), MC-LA (**41**), and MC-LV (**49**).

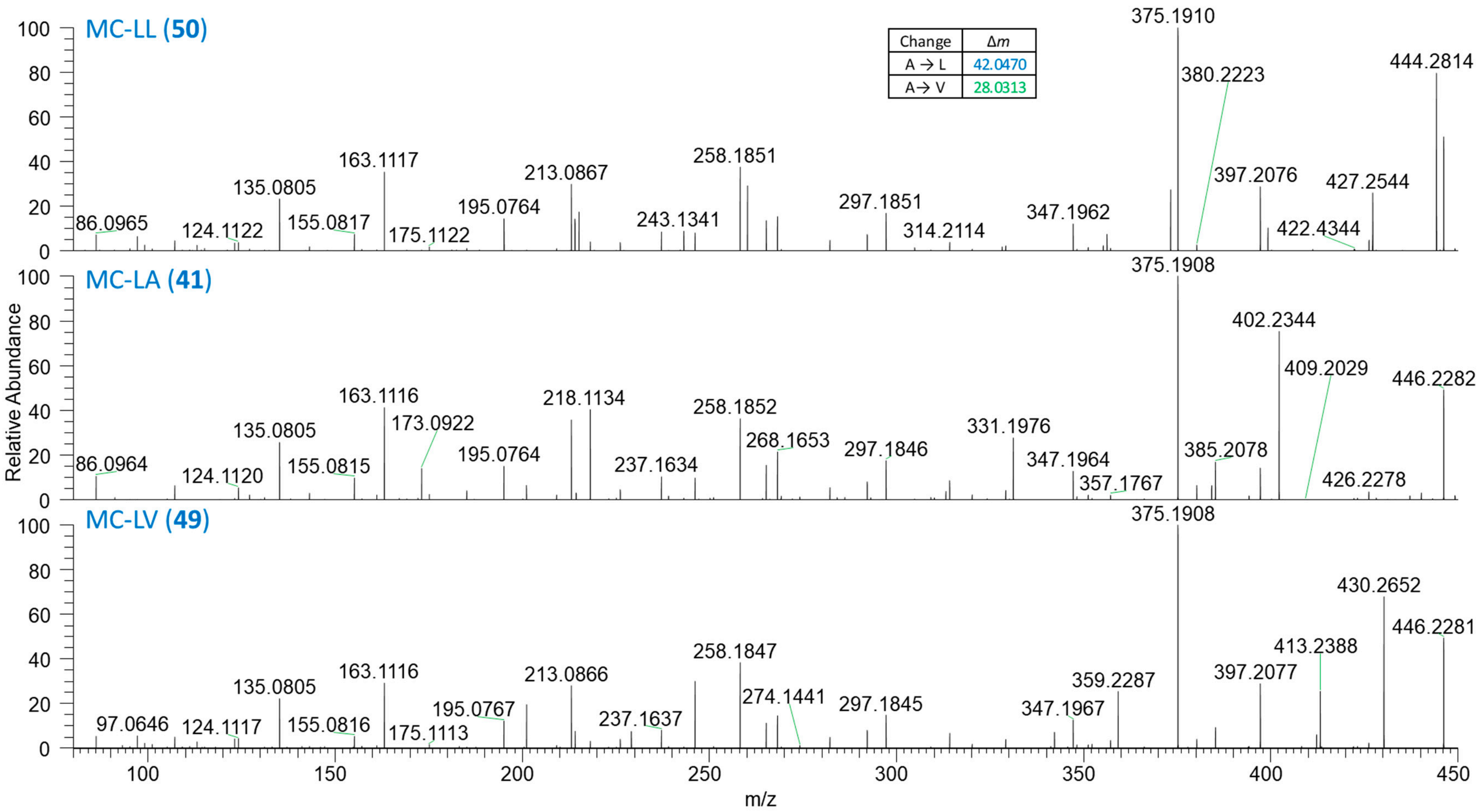

Figure S47. Figure S47. Expansion of LC–HRMS/MS spectra of [M+H]+ of MC-LL (**50**), MC-LA (**41**), and MC-LV (**49**).

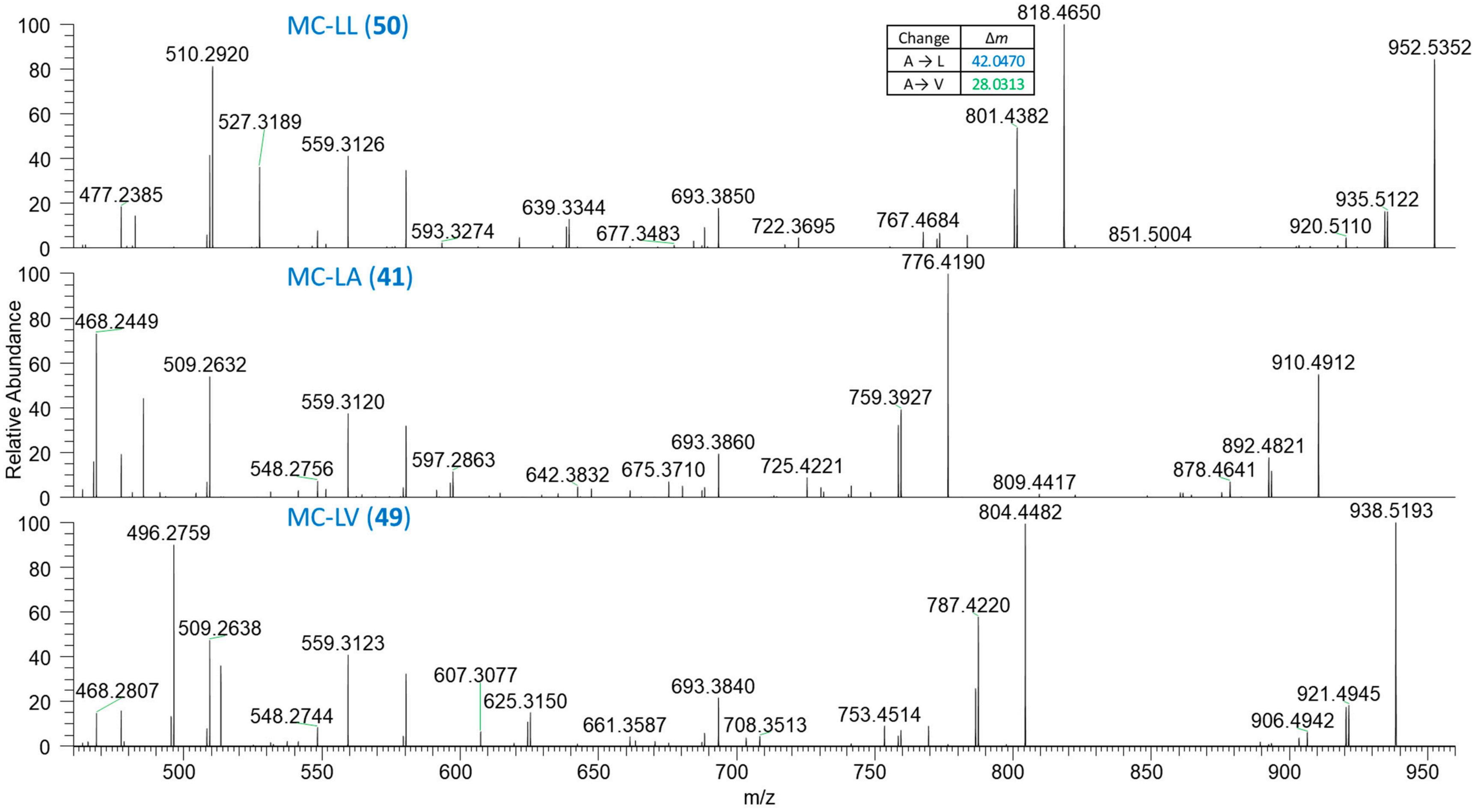

Figure S48. Expansion of LC–HRMS/MS spectra of [M+H]+ of MC-LL (**50**), MC-LA (**41**), and MC-LV (**49**).

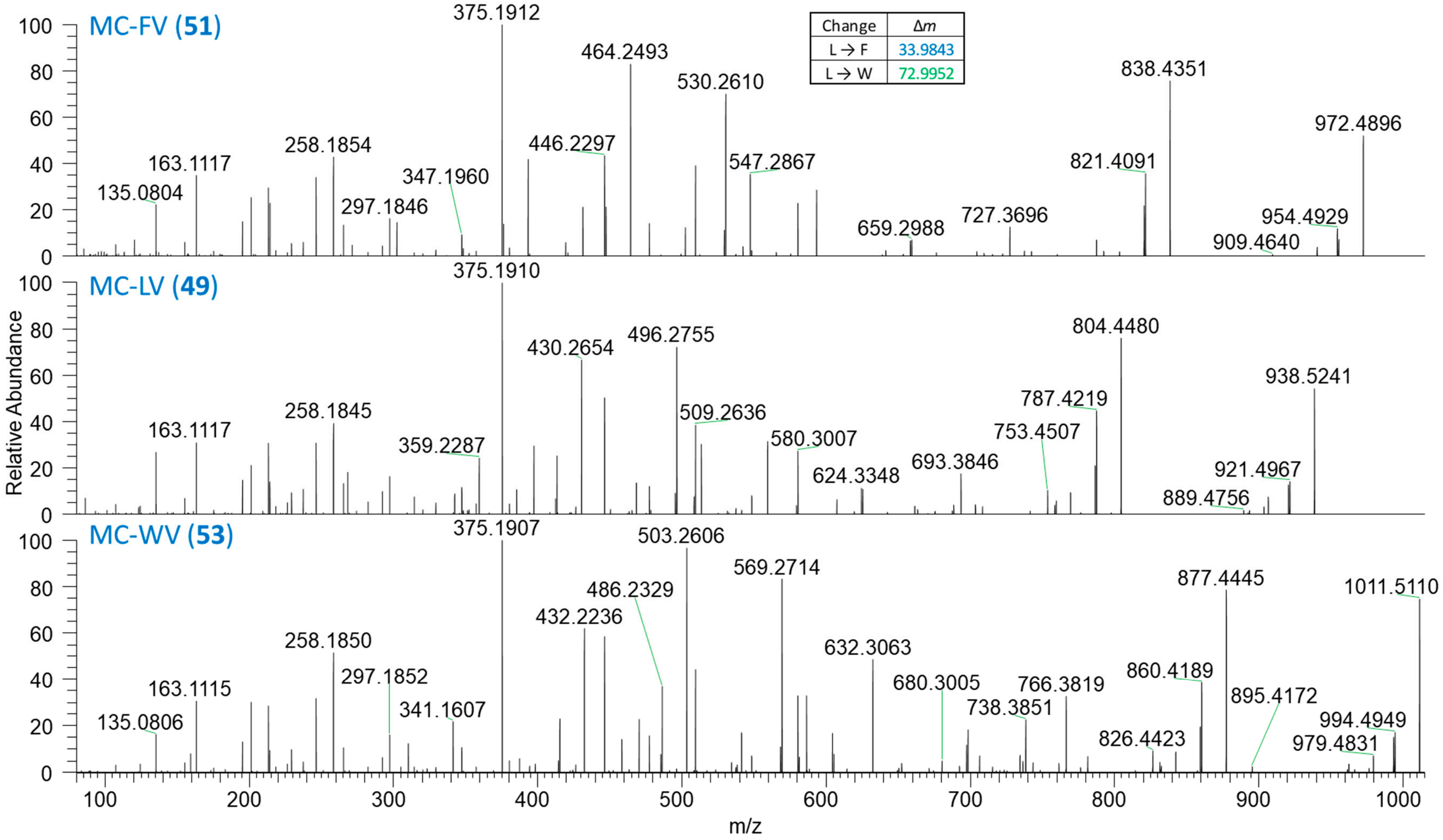

Figure S49. LC–HRMS/MS spectra of [M+H]+ of MC-FV (**51**), MC-LV (**49**), and MC-WV (**53**).

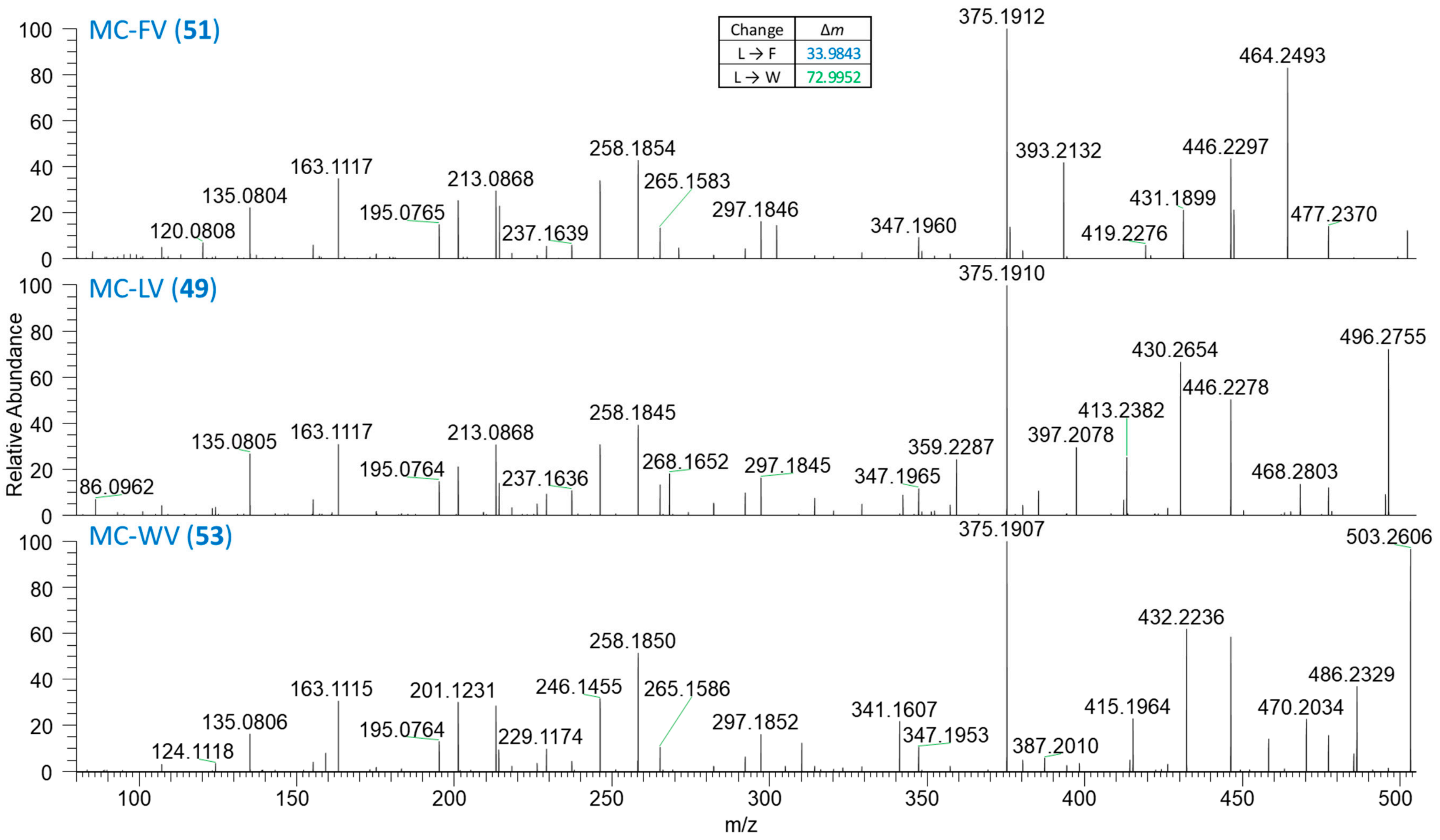

Figure S50. Expansion of LC–HRMS/MS spectra of $[M+H]^+$ of MC-FV (**51**), MC-LV (**49**), and MC-WV (**53**).

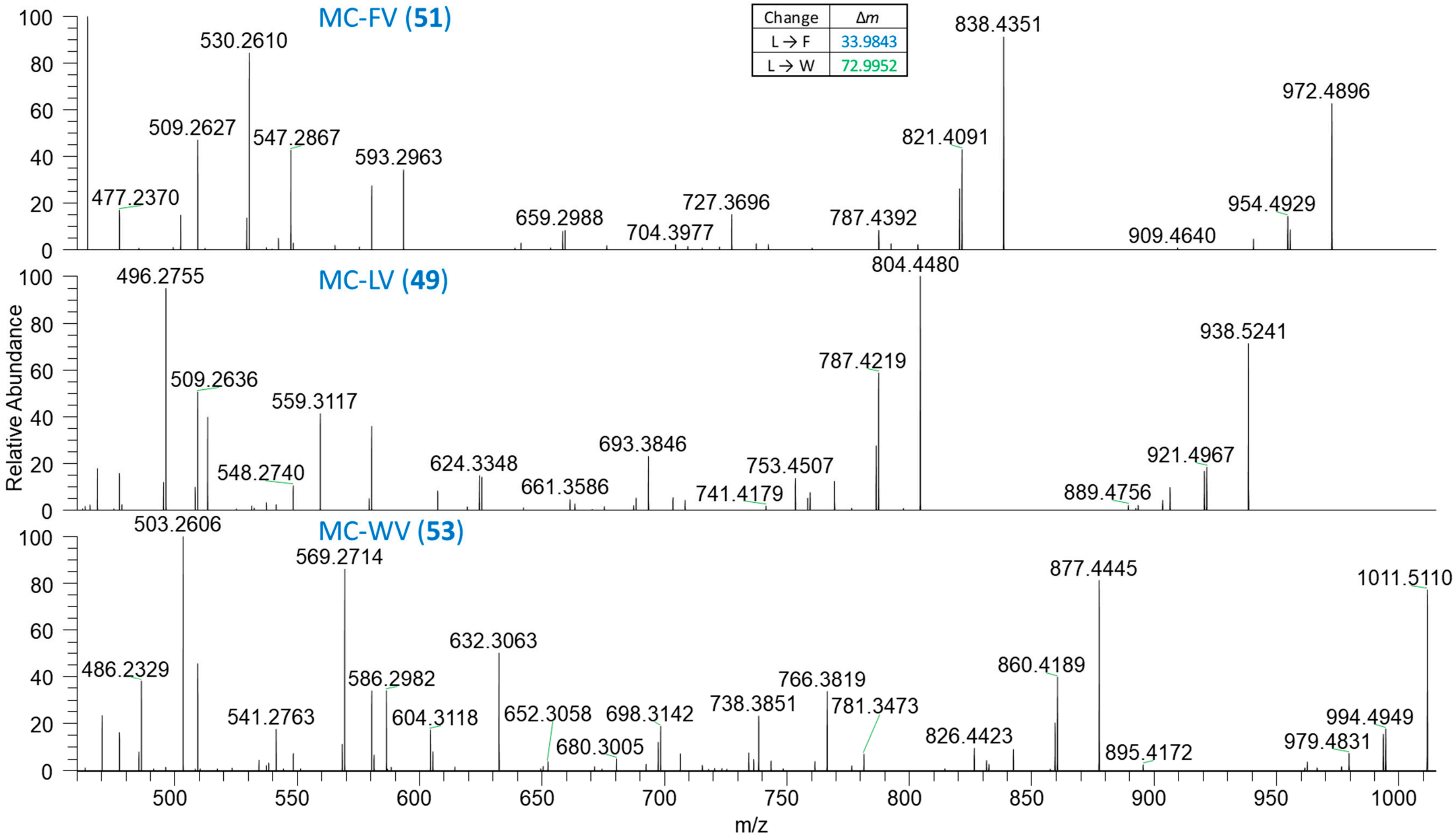

Figure S51. Expansion of LC–HRMS/MS spectra of [M+H]+ of MC-FV (**51**), MC-LV (**49**), and MC-WV (**53**).

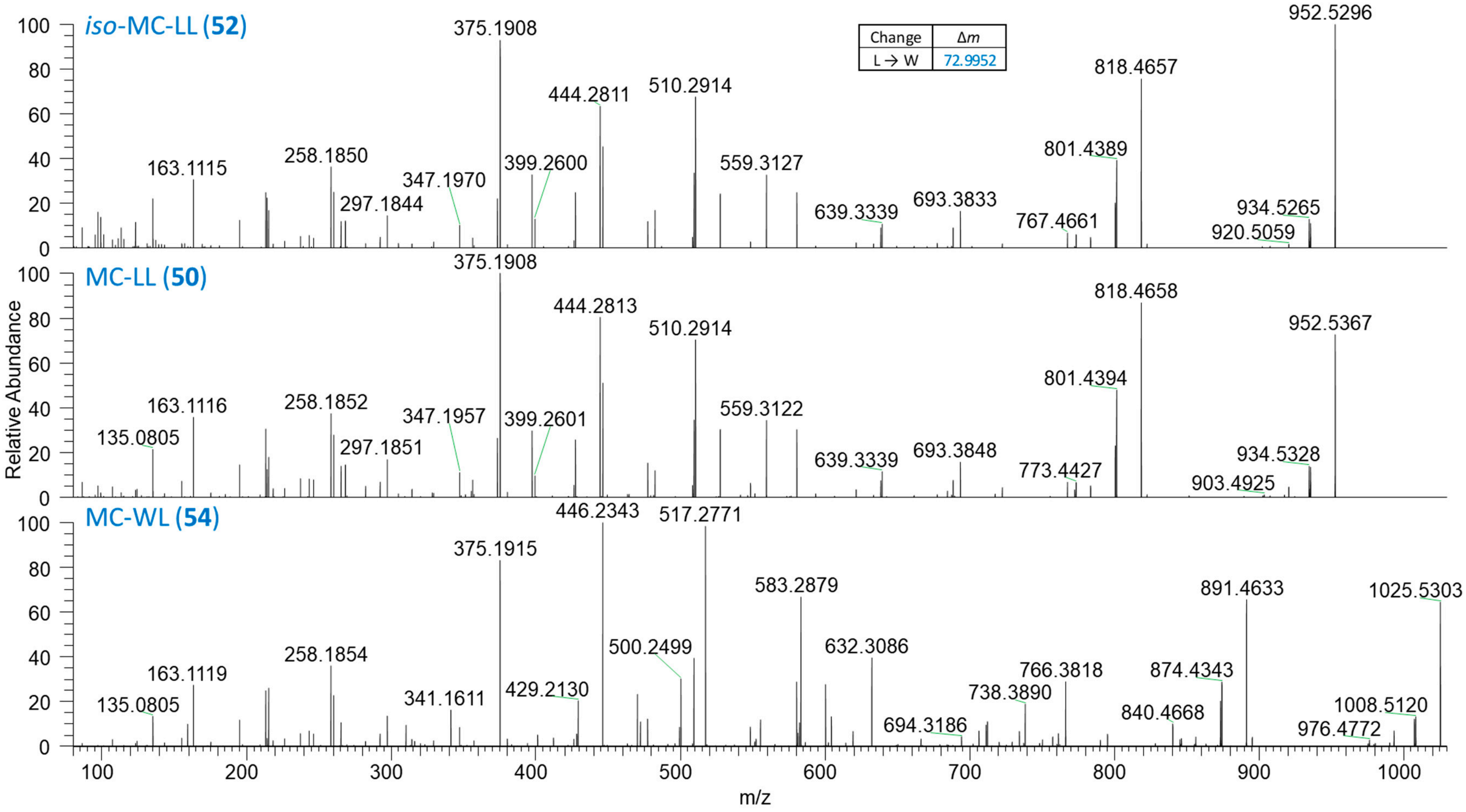

Figure S52. LC–HRMS/MS spectra of [M+H]$^+$ of *iso*-MC-LL (**52**), MC-LL (**50**), and MC-WL (**54**).

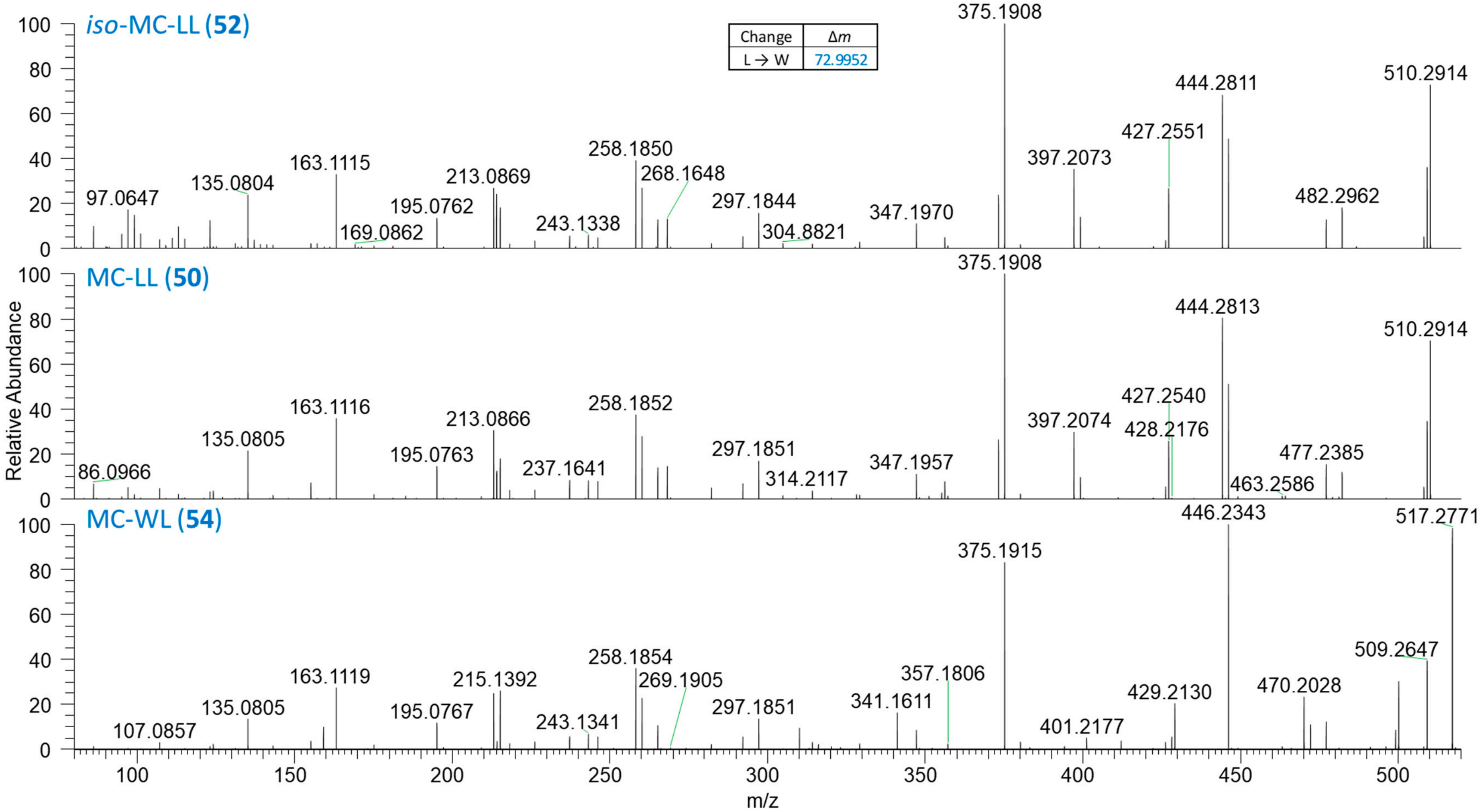

Figure S53. Expansion of LC–HRMS/MS spectra of [M+H]+ of *iso*-MC-LL (**52**), MC-LL (**50**), and MC-WL (**54**).

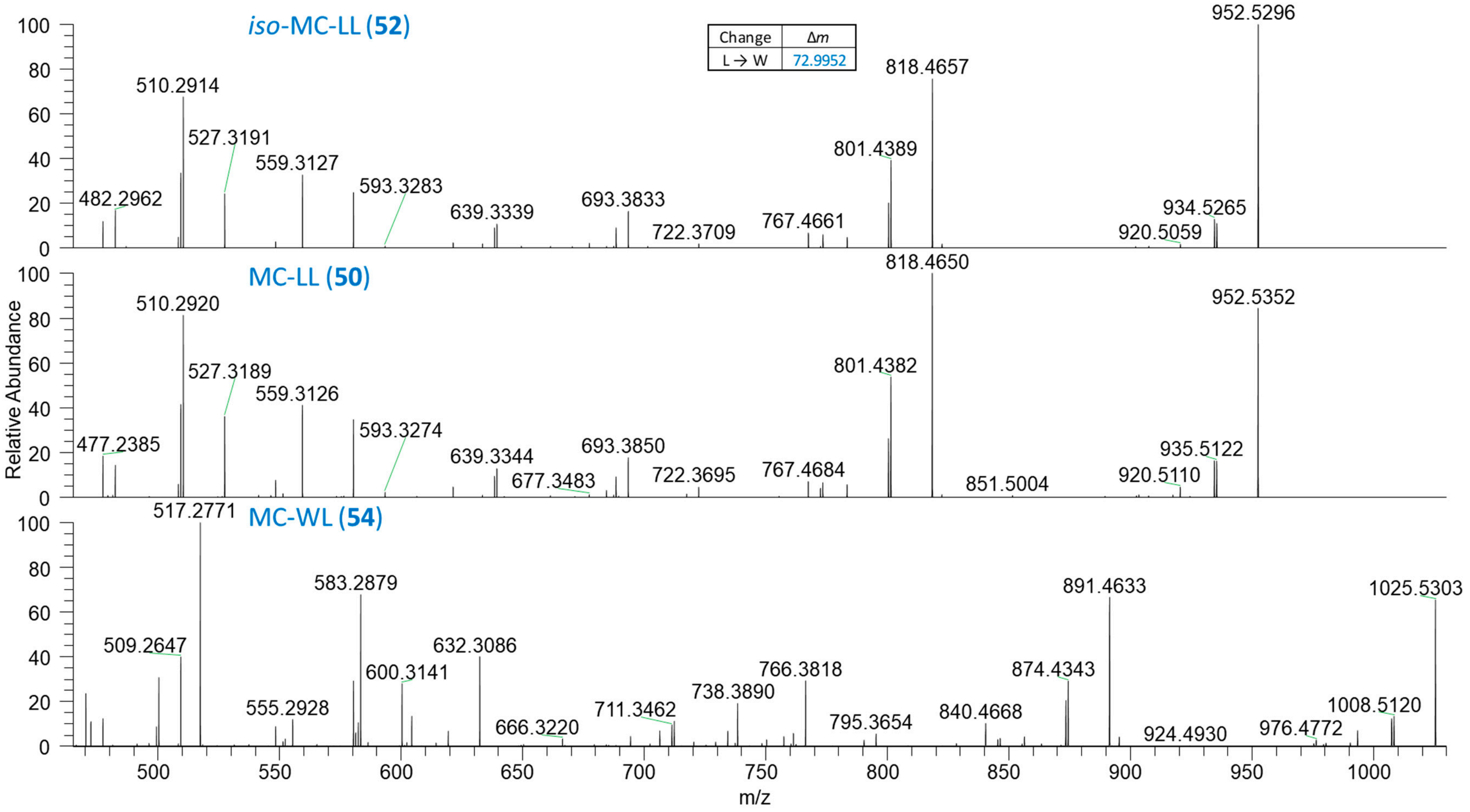

Figure S54. Expansion of LC–HRMS/MS spectra of [M+H]+ of *iso*-MC-LL (**52**), MC-LL (**50**), and MC-WL (**54**).

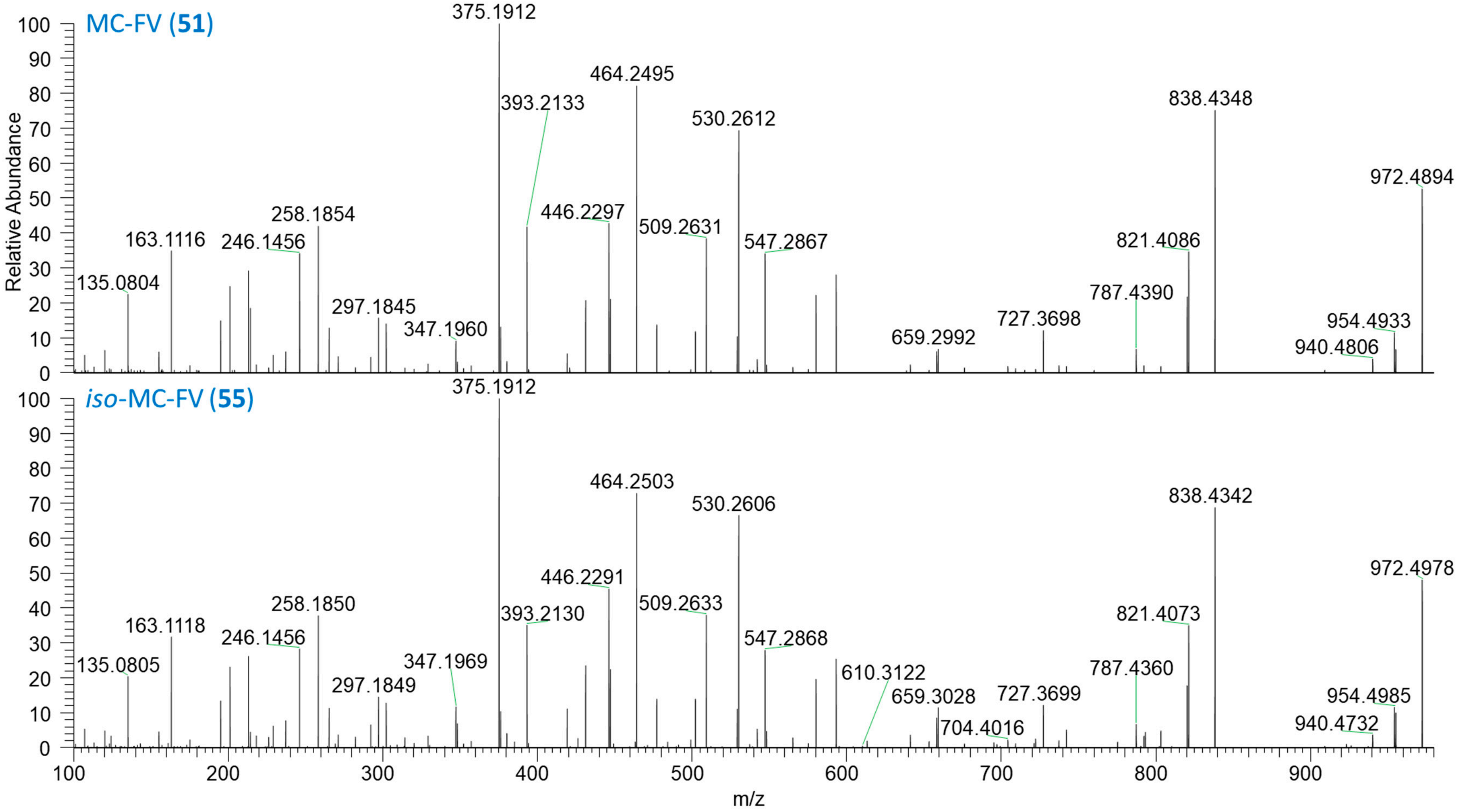

Figure S55. LC–HRMS/MS spectra of $[M+H]^+$ of MC-LL (**51**), and *iso*-MC-FV (**55**).

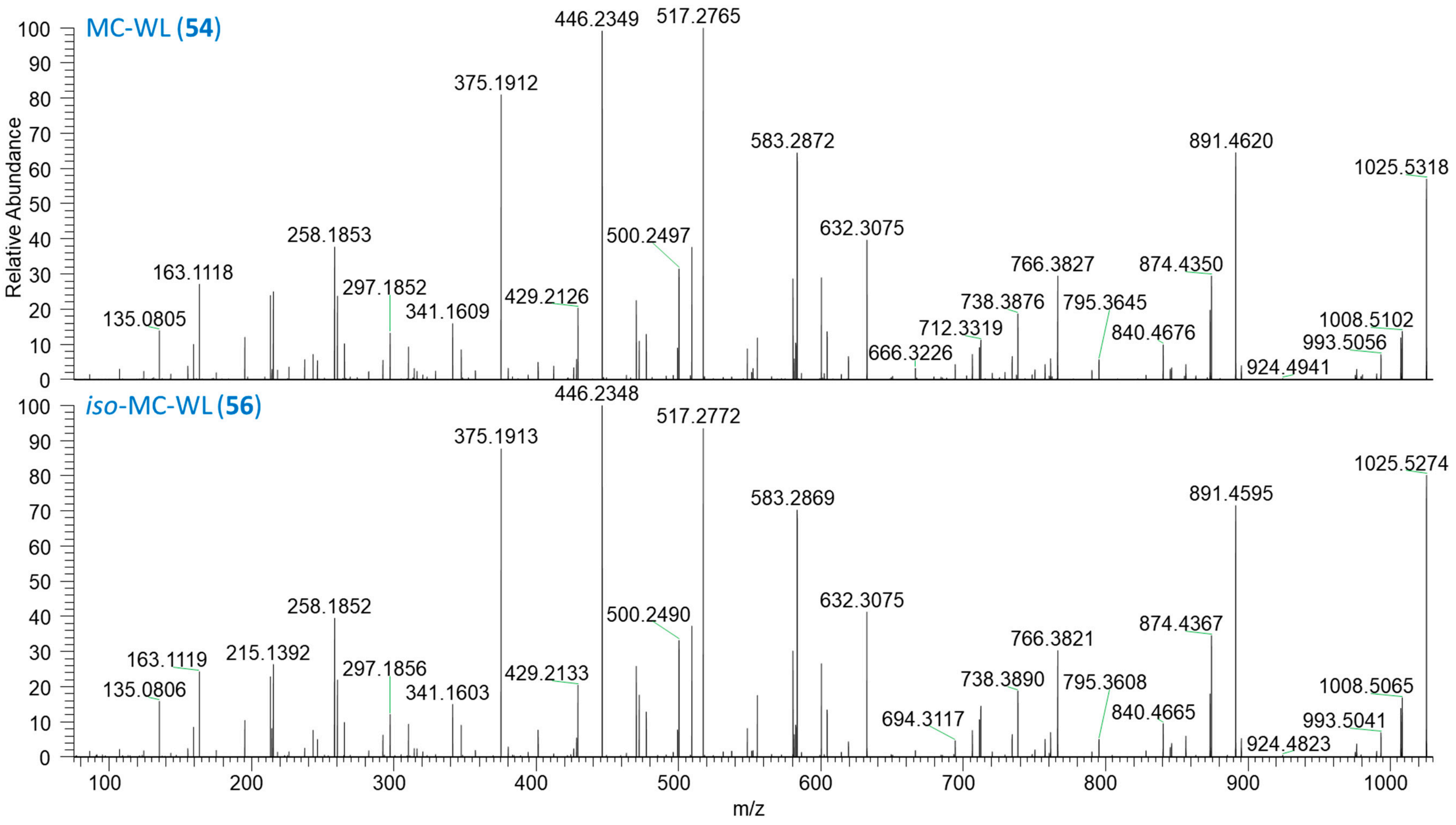

Figure S56. LC–HRMS/MS spectra of [M+H]+ of MC-WL (**54**), and *iso*-MC-WL (**56**).

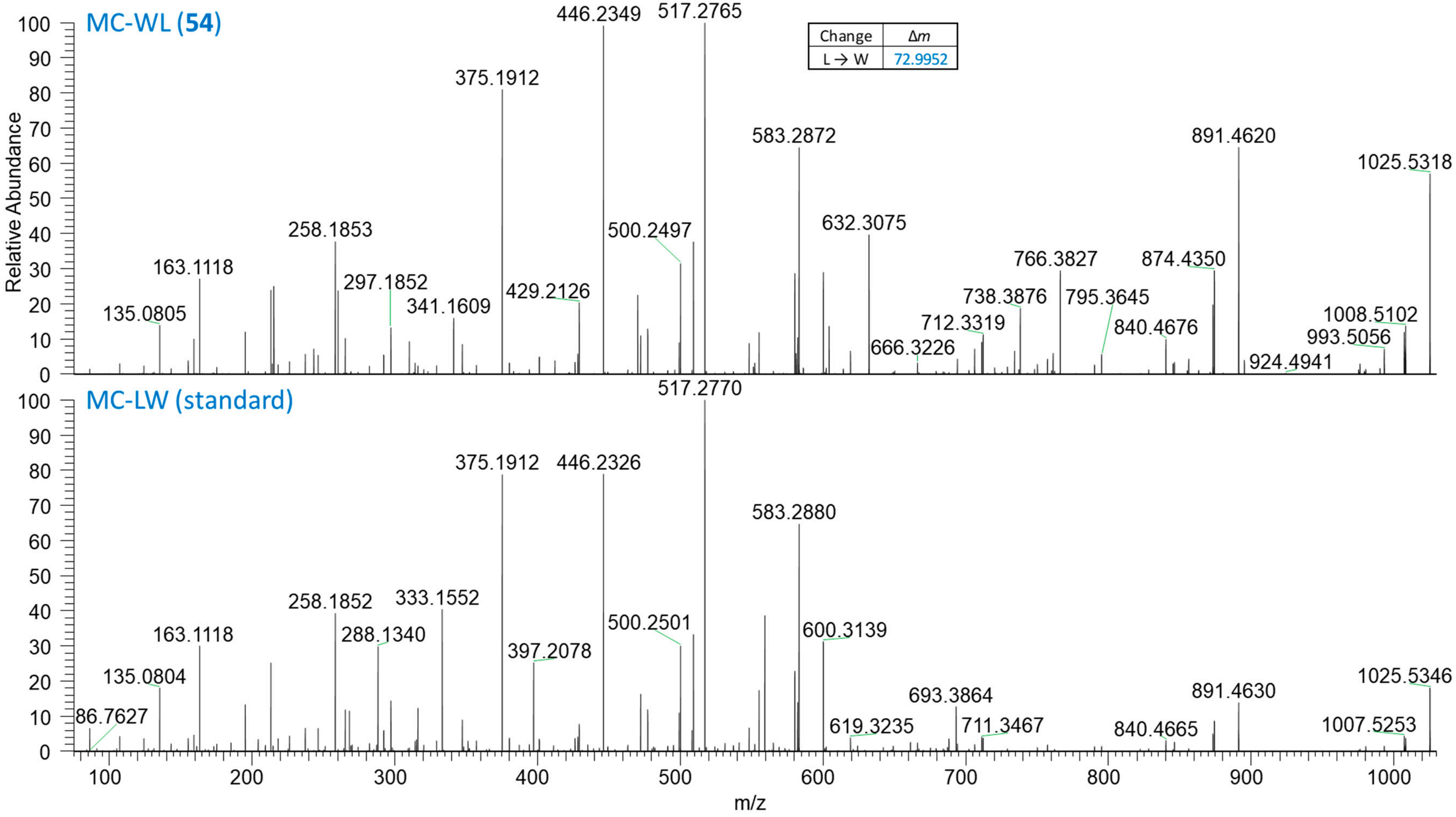

Figure S57. LC–HRMS/MS spectra of $[M+H]^+$ of MC-WL (**54**), and a standard of MC-LW.

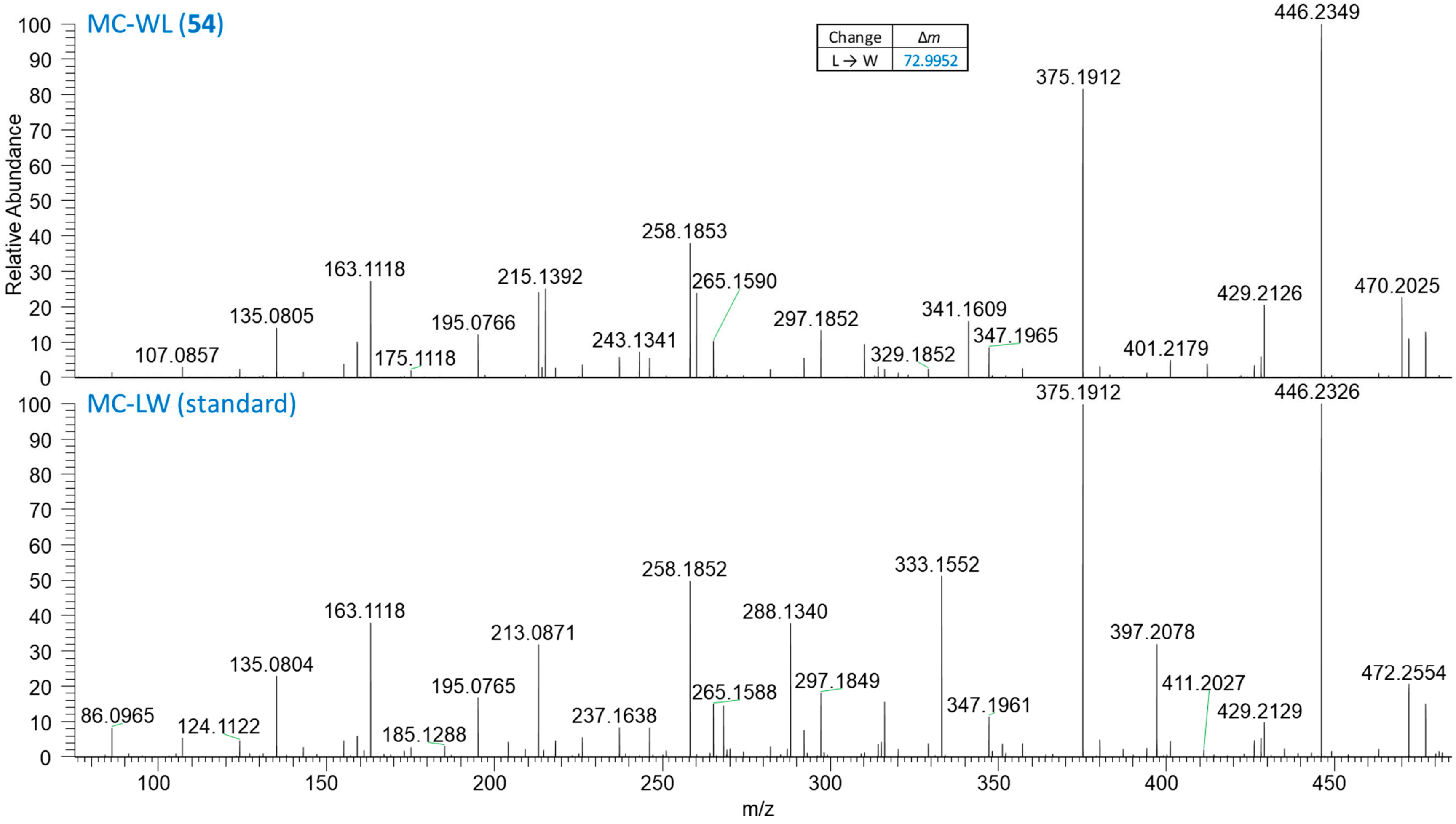

Figure S58. LC–HRMS/MS spectra of $[M+H]^+$ of MC-WL (**54**), and a standard of MC-LW.

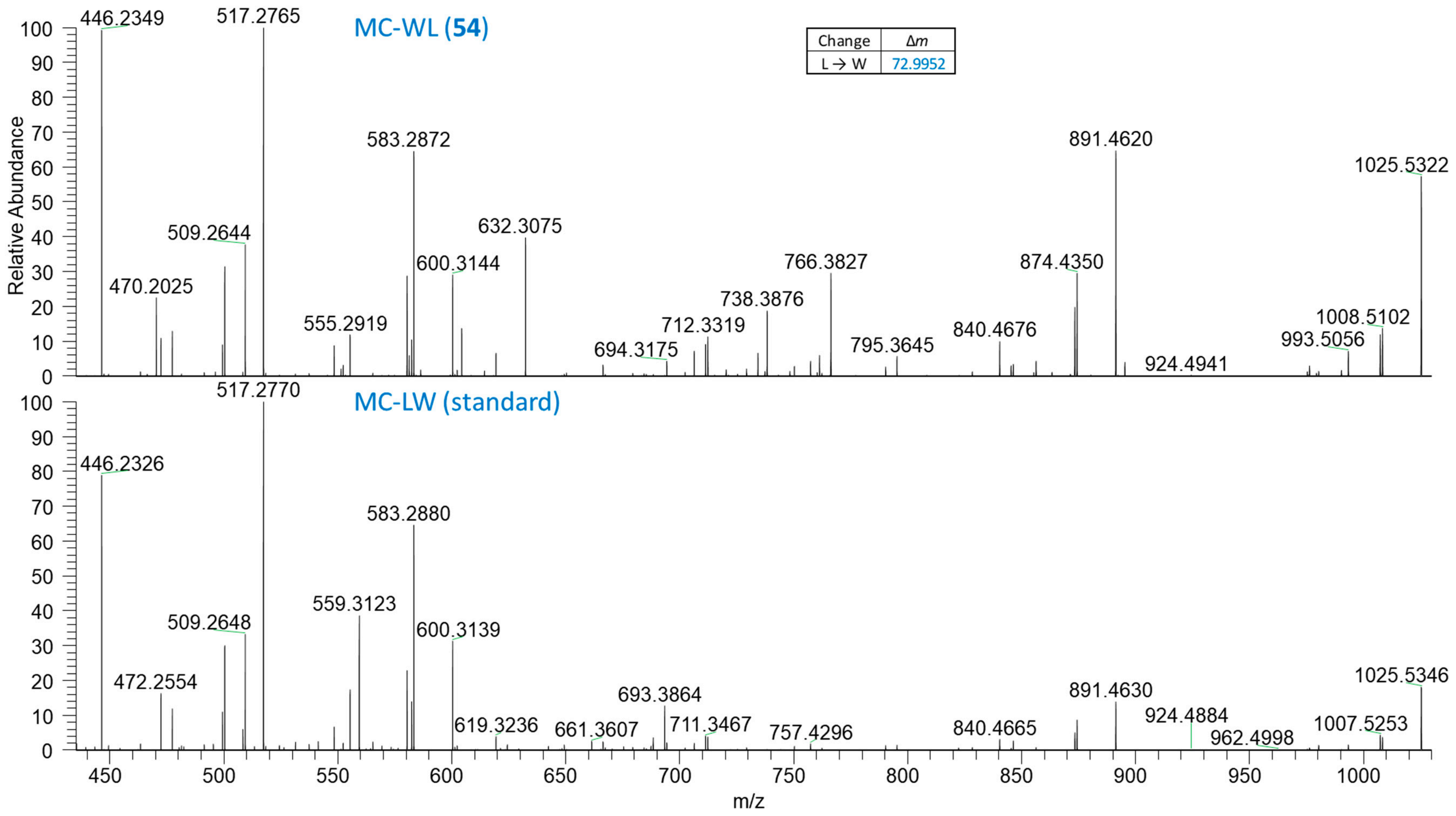

Figure S59. LC–HRMS/MS spectra of $[M+H]^+$ of MC-WL (**54**), and a standard of MC-LW.

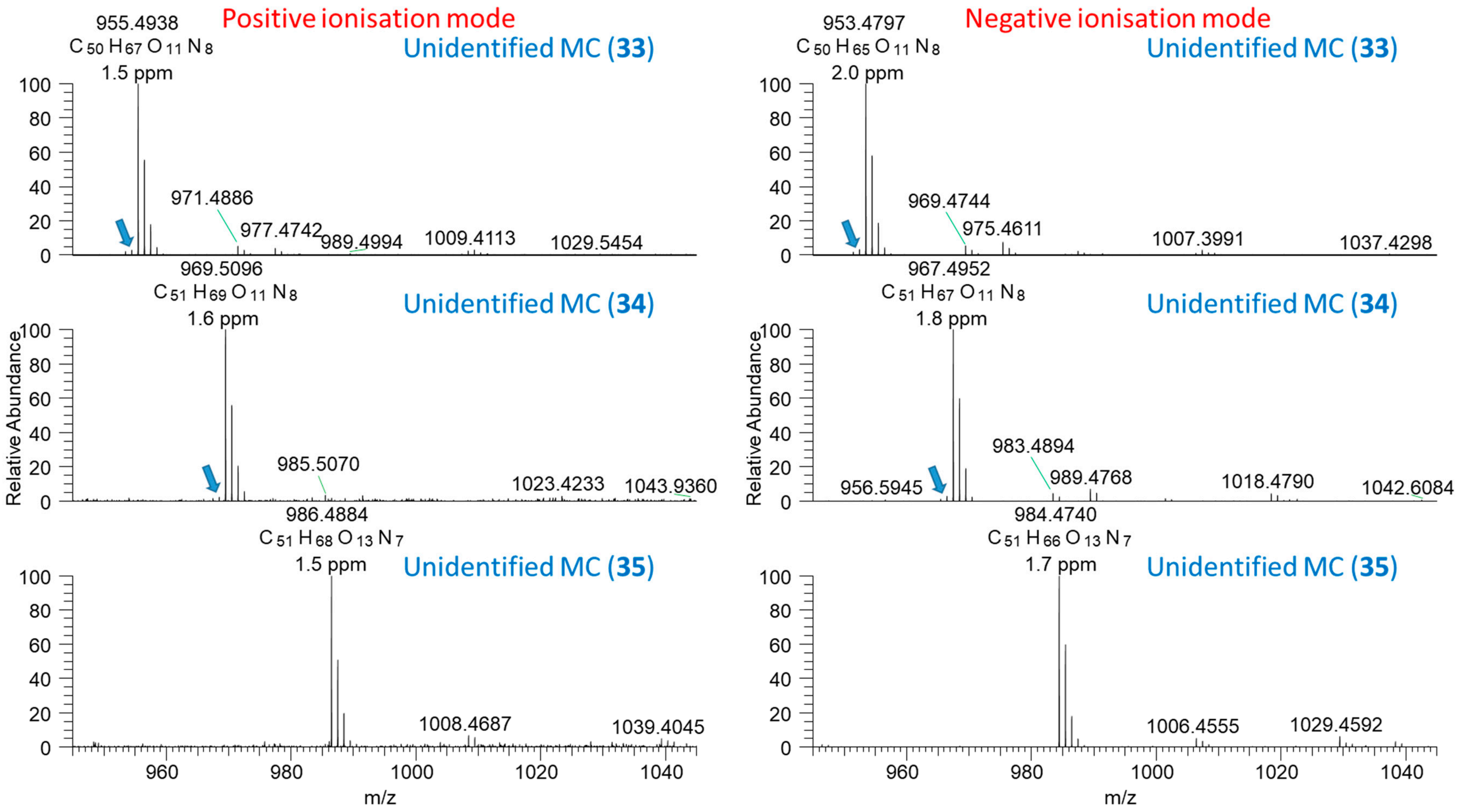

Figure S60. LC–HRMS full scan spectra of [M+H]+ of unidentified microcystins **33**–**35** in positive (left) and negative (right) ionisation modes. Note the peaks to the low-mass side of the main peaks for **33** and **34**, as well as the apparent presence of [M+15.9949], in both ionisation modes.
