# Supplementary material for: Cylindrospermopsin- and Deoxycylindrospermopsin-Producing Raphidiopsis raciborskii and Microcystin-Producing Microcystis spp. in Meiktila Lake, Myanmar
Source: Toxins (Basel). 2020 Apr 7;12(4):232. doi: 10.3390/toxins12040232 (PMC7232193; doi:10.3390/toxins12040232)
Supplement: Supplementary file 1 [file toxins-12-00232-s001.zip › toxins-757884 supp for ms update.pdf]

Supplementary Materials: Cylindrospermopsin- and Deoxycylindrospermopsin-Producing *Raphidiopsis raciborskii* and Microcystin-Producing *Microcystis* spp. in Meiktila Lake, Myanmar

Andreas Ballot, Thida Swe, Marit Mjelde, Leonardo Cerasino, Vladyslava Hostyeva and Christopher O. Miles

Table S1. Identities of microcystins detected by LC-HRMS/MS analysis in *M. aeruginosa* strains AB2017/14 and /15, their retention times (t_R), concentrations, elemental compositions, observed *m*/*z* values in positive and negative ionisation modes, whether they matched retention times in reference samples and whether characteristic microcystin product ions were observed. All compounds are microcystins, the confidence in the identity of the congener varies depending on the availability of standards for retention time reference and on the information obtained from the LC-HRMS analysis and chemical reactivity.[a]

					**Concentration**				**Accurate mass**										
					**AB2017/14**		**AB2017/15**					**Positive**		**Negative**				**MC Frag**	
	**Compound name**	**Confidence**		**t_R (min)**	**µg g⁻¹**	**%**	**µg g⁻¹**	**%**	**Neutral formula**	**RDBE**	***z***	***m*/*z***	**Δ (ppm)**	***m*/*z***	**Δ (ppm)**	**Ref**	**Thiol**	**Pos**	**Neg**
**1**	[D-Asp³]MC-RR	Confirmed	Known	4.18	2.4	0.21	1002.4	7.21	$C_{48}H_{73}N_{13}O_{12}$	19	2	512.7825	0.3	1022.5443	1.4	A	Yes	Yes	Yes
**2**	[Dha⁷]MC-RR	Probable	Known	4.53	0.1	0.01	31.8	0.23	$C_{48}H_{73}N_{13}O_{12}$	19	2	512.7819	−0.9	1022.5454	2.5	B	Yes	Yes	No
**3**	MC-RR	Confirmed	Known	4.55	19.4	1.74	1458.0	10.49	$C_{49}H_{75}N_{13}O_{12}$	19	2	519.7903	0.3	1036.5597	−0.2	A	Yes	Yes	Yes
**4**	[D-Asp³]MC-ER	Probable	Known	6.14	ND	-	119.6	0.86	$C_{47}H_{68}N_{10}O_{14}$	19	1	997.5016	2.7	995.4858	1.4	C	Yes	Yes	Yes
**5**	MC-LR–Cys[b]	Probable	Known	6.16	0.1	0.01	ND	-	$C_{52}H_{81}N_{11}O_{14}S$	18	2	558.7908	−1.3	1114.5657	4.0		No	Yes	Yes
**6**	MC-(H2)YR	Probable	Known	6.18	32.4	2.90	ND	-	$C_{52}H_{74}N_{10}O_{13}$	21	1	1047.5516	0.6	1045.5378	1.3		Yes	Yes	Yes
**7**	[D-Asp³]MC-(H2)YR	Probable	New	6.20	32.9	2.95	ND	-	$C_{51}H_{72}N_{10}O_{13}$	21	1	1033.5370	1.6	1031.5224	1.6		Yes	Yes	Yes
**8**	[D-Asp³]MC-(H4)YR	Probable	Known	6.27	0.6	0.05	148.1	1.07	$C_{51}H_{74}N_{10}O_{13}$	20	1	1035.5525	1.5	1033.5381	1.6	B	Yes	Yes	Yes
**9**	MC-ER	Probable	New	6.55	0.6	0.05	37.8	0.27	$C_{48}H_{70}N_{10}O_{14}$	19	1	1011.5158	1.2	1009.5015	1.5		Yes	Yes	Yes
**10**	[D-Asp³]MC-YR	Probable	Known	6.59	ND	-	144.1	1.04	$C_{51}H_{70}N_{10}O_{13}$	22	1	1031.5204	0.7	1029.5072	−1.2		Yes	Yes	No
**11**	MC-(H4)YR	Probable	Known	6.64	6.4	0.57	310.8	2.24	$C_{52}H_{76}N_{10}O_{13}$	20	1	1049.5683	1.6	1047.5540	1.9	B	Yes	Yes	Yes
**12**	[Mser⁷]MC-LR	Probable	Known	6.80	0.4	0.04	13.3	0.10	$C_{49}H_{76}N_{10}O_{13}$	17	1	1013.5668	0.2	1011.5530	0.9	B	No	Yes	No
**13**	[D-Asp³]MC-LR	Confirmed	Known	6.89	40.8	3.65	1870.3	13.45	$C_{48}H_{72}N_{10}O_{12}$	18	1	981.5416	1.2	979.5273	1.5	A	Yes	Yes	Yes
**14**	MC-YR	Confirmed	Known	6.99	9.1	0.81	317.4	2.28	$C_{52}H_{72}N_{10}O_{13}$	22	1	1045.5369	1.5	1043.5224	2.6	A	Yes	Yes	Yes
**15**	MC-MR[b,c]	Probable	Known	6.99	3.6	0.32	ND	-	$C_{48}H_{72}N_{10}O_{12}S$	18	1	1013.5135	1.0	1011.4999	1.9		Yes	Yes	Yes
**16**	MC-Y(OMe)R	Probable	Known	7.07	7.2	0.64	ND	-	$C_{53}H_{74}N_{10}O_{14}$	22	1	1075.5475	1.5	1073.5329	1.5	B	Yes	Yes	Yes
**17**	MC-LR	Confirmed	Known	7.12	183.0	16.38	3332.5	23.97	$C_{49}H_{74}N_{10}O_{12}$	18	1	995.5568	0.7	993.5435	2.0	A	Yes	Yes	Yes
**18**	[Dha⁷]MC-LR	Confirmed	Known	7.13	0.8	0.07	37.4	0.27	$C_{48}H_{72}N_{10}O_{12}$	18	1	981.5410	0.6	979.5289	3.1	A	Yes	No	No
**19**	Unidentified MC	Unidentified	New	7.15	59.0	5.28	ND	-	$C_{54}H_{71}N_{11}O_{13}$	25	1	1082.5322	1.5	1080.5170	0.9		Yes	Yes	Yes
**20**	MC-KynR	Probable	Known	7.35	4.5	0.40	ND	-	$C_{53}H_{73}N_{11}O_{13}$	23	1	1072.5478	1.5	1070.5332	1.4	B	Yes	No	Yes
**21**	MC-HilR	Confirmed	Known	7.38	41.8	3.74	17.9	0.13	$C_{50}H_{76}N_{10}O_{12}$	18	1	1009.5726	0.9	1007.5582	1.0	A	Yes	Yes	Yes
**22**	Unidentified	Unidentified	New	7.38	59.6	5.34	ND	-	$C_{54}H_{71}N_{11}O_{13}$	25	1	1082.5321	1.4	1080.5169	0.8		Yes	Yes	Yes
**23**	MC-OiaR	Tentative	Known	7.40	102.4	9.17	ND	-	$C_{54}H_{73}N_{11}O_{13}$	24	1	1084.5460	−0.2	1082.5311	−0.5		Yes	Yes	Yes
**24**	[D-Asp³]MC-RE	Probable	New	7.43	ND	-	110.1	0.79	$C_{47}H_{68}N_{10}O_{14}$	19	1	997.5004	1.5	995.4857	1.3		Yes	Yes	Yes
**25**	MC-FR	Confirmed	Known	7.48	255.2	22.85	ND	-	$C_{52}H_{72}N_{10}O_{12}$	22	1	1029.5408	0.4	1027.5269	1.1	A	Yes	Yes	Yes

**26**	MC-WR	Confirmed	Known	7.64	0.7	0.06	ND	-	$C_{54}H_{73}N_{11}O_{12}$	24	1	1068.5517	0.3	1066.5380	1.2	A	Yes	Yes	Yes
**27**	MC-RE	Probable	New	7.79	3.8	0.34	1281.0	9.21	$C_{48}H_{70}N_{10}O_{14}$	19	1	1011.5161	0.4	1009.5015	1.4		Yes	Yes	Yes
**28**	MC-RA	Probable	Known	8.24	13	1.16	ND	-	$C_{46}H_{68}N_{10}O_{12}$	18	1	953.5100	1.0	951.4955	1.0	D	Yes	Yes	Yes
**29**	MC-RAbu	Probable	Known	8.83	7.1	0.64	ND	-	$C_{47}H_{70}N_{10}O_{12}$	18	1	967.5255	0.8	965.5116	1.5	D	Yes	Yes	Yes
**30**	MC-(H2)YG	Probable	New	9.06	5.4	0.48	ND	-	$C_{48}H_{65}N_{7}O_{13}$	20	1	948.4720	0.7	946.4577	1.0		Yes	Yes	Yes
**31**	MC-RM[b]	Probable	New	9.23	0.2	0.02	ND	-	$C_{48}H_{72}N_{10}O_{12}S$	18	1	1013.5095	−3.0	1011.4966	−1.3		Yes	Yes	No
**32**	MC-(H2)YA	Probable	New	9.34	10.1	0.90	ND	-	$C_{49}H_{67}N_{7}O_{13}$	20	1	962.4880	1.1	960.4738	1.4		Yes	Yes	Yes
**33**	Unidentified MC	Unidentified	New	9.50	12	1.07	ND	-	$C_{50}H_{66}N_{8}O_{11}$	22	1	955.4929	0.5	953.4783	0.5		Yes	Yes	Yes
**34**	Unidentified MC	Unidentified	New	9.78	2.3	0.21	ND	-	$C_{51}H_{68}N_{8}O_{11}$	22	1	969.5089	0.9	967.4942	0.7		Yes	Yes	Yes
**35**	Unidentified MC	Unidentified	New	9.96	3.7	0.33	ND	-	$C_{51}H_{67}N_{7}O_{13}$	22	1	986.4877	0.7	984.4735	1.1		Yes	Yes	No
**36**	[D-Asp^3]MC-EE	Probable	Known	11.43	ND	-	403.3	2.90	$C_{46}H_{63}N_{7}O_{16}$	19	1	970.4421	1.8	968.4272	1.4	C	Yes	Yes	Yes
**37**	MC-EE	Probable	New	12.58	0.9	0.08	1533.5	11.03	$C_{47}H_{65}N_{7}O_{16}$	19	1	984.4578	1.8	982.4432	1.7		Yes	Yes	Yes
**38**	[D-Asp^3]MC-LE	Probable	New	12.82	1.2	0.11	358.8	2.58	$C_{47}H_{67}N_{7}O_{14}$	18	1	954.4832	1.4	952.4690	1.8		Yes	Yes	Yes
**39**	[D-Asp^3]MC-LA	Probable	Known	13.32	6.5	0.58	ND	-	$C_{45}H_{65}N_{7}O_{12}$	17	1	896.4770	0.7	894.4631	1.4		Yes	Yes	Yes
**40**	MC-LE	Probable	New	13.69	12.9	1.15	1376.1	9.90	$C_{48}H_{69}N_{7}O_{14}$	18	1	968.4982	0.7	966.4849	2.0		Yes	Yes	Yes
**41**	MC-LA	Confirmed	Known	14.87	44.8	4.01	ND	-	$C_{46}H_{67}N_{7}O_{12}$	17	1	910.4920	−0.1	908.4788	1.4	A	Yes	Yes	Yes
**42**	MC-HilA	Probable	New	15.29	5.9	0.53	ND	-	$C_{47}H_{69}N_{7}O_{12}$	17	1	924.5076	-0.1	922.4942	1.1		Yes	Yes	Yes
**43**	MC-WA	Probable	Known	15.88	4.4	0.39	ND	-	$C_{51}H_{66}N_{8}O_{12}$	23	1	983.4874	0.1	981.4737	1.0		Yes	Yes	Yes
**44**	MC-FA	Probable	Known	15.95	42.3	3.79	ND	-	$C_{49}H_{65}N_{7}O_{12}$	21	1	944.4767	0.4	942.4629	1.1		Yes	Yes	Yes
**45**	MC-LAbu	Probable	Known	16.09	16.6	1.49	ND	-	$C_{47}H_{69}N_{7}O_{12}$	17	1	924.5078	−1.1	922.4943	1.2	D	Yes	Yes	Yes
**46**	MC-HilAbu	Probable	New	16.55	2.2	0.20	ND	-	$C_{48}H_{71}N_{7}O_{12}$	17	1	938.5233	0.0	936.5110	2.3		Yes	Yes	Yes
**47**	MC-WAbu	Probable	Known	17.20	13.4	1.20	ND	-	$C_{52}H_{68}N_{8}O_{12}$	23	1	997.5030	0.0	995.4898	1.4		Yes	Yes	Yes
**48**	MC-FAbu	Probable	Known	17.39	26.4	2.36	ND	-	$C_{50}H_{67}N_{7}O_{12}$	21	1	958.4921	0.0	956.4784	0.9		Yes	Yes	Yes
**49**	MC-LV	Probable	Known	17.62	3.6	0.32	ND	-	$C_{48}H_{71}N_{7}O_{12}$	17	1	938.5228	−0.6	936.5106	1.9		Yes	Yes	Yes
**50**	MC-LL	Probable	Known	17.93	3.9	0.35	ND	-	$C_{49}H_{73}N_{7}O_{12}$	17	1	952.5388	−0.2	950.5254	1.0	D	Yes	Yes	Yes
**51**	MC-FV	Probable	New	18.40	0.7	0.06	ND	-	$C_{51}H_{69}N_{7}O_{12}$	21	1	972.5072	−0.5	970.4944	1.3		Yes	Yes	No
**52**	*iso*-MC-LL	Tentative	New	18.49	1.2	0.11	ND	-	$C_{49}H_{73}N_{7}O_{12}$	17	1	952.5388	−0.2	950.5262	1.8		Yes	Yes	Yes
**53**	MC-WV	Probable	New	18.54	2.9	0.26	ND	-	$C_{53}H_{70}N_{8}O_{12}$	23	1	1011.5166	−1.9	1009.5041	0.0		Yes	Yes	Yes
**54**	MC-WL	Probable	Known	18.74	2.8	0.25	ND	-	$C_{54}H_{72}N_{8}O_{12}$	23	1	1025.5337	−0.6	1023.5210	1.3		Yes	Yes	Yes
**55**	*iso*-MC-FV	Tentative	New	18.79	2.1	0.19	ND	-	$C_{51}H_{69}N_{7}O_{12}$	21	1	972.5073	−0.5	970.4942	1.1		Yes	Yes	Yes
**56**	*iso*-MC-WL	Tentative	New	19.16	1.3	0.12	ND	-	$C_{54}H_{72}N_{8}O_{12}$	23	1	1025.5344	0.2	1023.5216	1.9		Yes	Yes	Yes
				Total:	1117	100.0	13904	100.0											

[a] Concentration expressed per weight of biomass and as a percentage of total microcystins detected in each culture; Proposed elemental composition and calculated number of rings plus double-bond equivalents (RDBE); Accurate mass measured by LC-HRMS, with observed mass errors and charge z in positive mode (all negative ions were singly-charged); Ref, reference; A, standard, or minor analogue in a standard; B, samples from Yilmaz et al.[1]; C, *Planktothrix prolifica* from Mallia et al.[2]; D, Bloom samples from L. Victoria[3]; Thiol, reacted with mercaptoethanol indicating presence of Mdha[7] or Dha[7] (Miles et al.[4]); MC Frags, characteristic microcystin fragments (Figure 2) observed in either positive or negative ionization modes; [b] Reacted with Oxone/DMSO, confirming the presence of an oxidisable sulfide moiety.[5]; [c] A good quality confirmatory MS/MS spectrum of the Oxone/DMSO oxidation product, MC-M(O)R, was obtained showing the characteristic product ions for this compound.[5].

References:

1. Yilmaz, Y.; Foss, A. J.; Miles, C. O.; Özen, M.; Demir, N.; Balci, M.; Beach, D. G., Comprehensive multi-technique approach reveals the high diversity of microcystins in field collections and an associated isolate of *Microcystis aeruginosa* from a Turkish lake. *Toxicon* **2019,** *167*, 87-100.
2. Mallia, V.; Uhlig, S.; Rafuse, C.; Meija, J.; Miles, C. O., Novel microcystins from *Planktothrix prolifica* NIVA-CYA 544 identified by LC-MS/MS, functional group derivatization and ^{15}N-labeling. *Mar. Drugs* **2019,** *17* (11), 643.
3. Miles, C. O.; Sandvik, M.; Nonga, H. E.; Rundberget, T.; Wilkins, A. L.; Rise, F.; Ballot, A., Identification of microcystins in a Lake Victoria cyanobacterial bloom using LC-MS with thiol derivatization. *Toxicon* **2013,** *70*, 21-31.
4. Miles, C. O.; Sandvik, M.; Haande, S.; Nonga, H.; Ballot, A., LC-MS analysis with thiol derivatization to differentiate [Dhb^7]- from [Mdha^7]-microcystins: analysis of cyanobacterial blooms, *Planktothrix* cultures and European crayfish from Lake Steinsfjorden, Norway. *Environ. Sci. Technol.* **2013,** *47* (9), 4080-4087.
5. Miles, C. O.; Melanson, J. E.; Ballot, A., Sulfide oxidations for LC-MS analysis of methionine-containing microcystins in *Dolichospermum flos-aquae* NIVA-CYA 656. *Environ. Sci. Technol.* **2014,** *48*, 13307−13315.
